# Reactivity of Imidazole‐ and Pyridine‐Carboxaldehydes for *Gem*‐Diol and Hemiacetal Generation: Theoretical and Experimental Insights

**DOI:** 10.1002/open.202400411

**Published:** 2025-01-05

**Authors:** Ayelén F. Crespi, Emiliano Barrionuevo, Gabriel Jasinski, Albertina G. Moglioni, Daniel Vega, Juan M. Lázaro‐Martínez

**Affiliations:** ^1^ Universidad de Buenos Aires Facultad de Farmacia y Bioquímica Department of Chemistry Ciudad Autónoma de Buenos Aires 1113 Argentina; ^2^ CONICET – Universidad de Buenos Aires Instituto de Química y Metabolismo del Fármaco (IQUIMEFA-UBA-CONICET) Ciudad Autónoma de Buenos Aires 1113 Argentina; ^3^ Universidad de Buenos Aires Facultad de Farmacia y Bioquímica Department of Pharmacology Ciudad Autónoma de Buenos Aires 1113 Argentina; ^4^ Department of Condensed Matter Physics Comisión Nacional de Energía Atómica San Martín, Buenos Aires 1650 Argentina

**Keywords:** Pyridinecarboxaldehydes, Imidazolecarboxaldehydes, NMR, *Gem*-diol, Theoretical calculations

## Abstract

*Gem*‐diols are defined as organic molecules carrying two hydroxyl groups at the same carbon atom, which is the result of the nucleophilic addition of water to a carbonyl group. In this work, the generation of the hydrated or hemiacetal forms using pyridine‐ and imidazole‐carboxaldehyde isomers in different chemical environments was studied by Nuclear Magnetic Resonance (NMR) recorded in different media and combined with theoretical calculations. The change in the position of aldehyde group in either the pyridine or the imidazole ring had a clear effect in the course of the hydration/hemiacetal generation reaction, which was favored in protic solvents mainly in the presence of methanol. For pyridinecarboxaldehydes, the acidity/basicity degree of the reaction medium influenced not only the generation of the *gem*‐diol or hemiacetal forms but also the oxidation to the corresponding carboxylic acid. However, imidazolecarboxaldehyde was found to be less reactive to the nucleophilic addition of water and methanol than the other compounds in all the environments evaluated. Furthermore, both the *gem*‐diol/hemiacetal generation and the Cannizzaro reaction products were studied in alkaline medium.

## Graphical abstract

The generation of the gem‐diol and hemiacetal forms using pyridine‐ and imidazole‐carboxaldehyde isomers in different chemical environments was studied by NMR spectra and combined with theoretical calculations. Unlike aprotic solvents, protic ones, as water and methanol, favored the generation of the gem‐diol and hemiacetal structures.

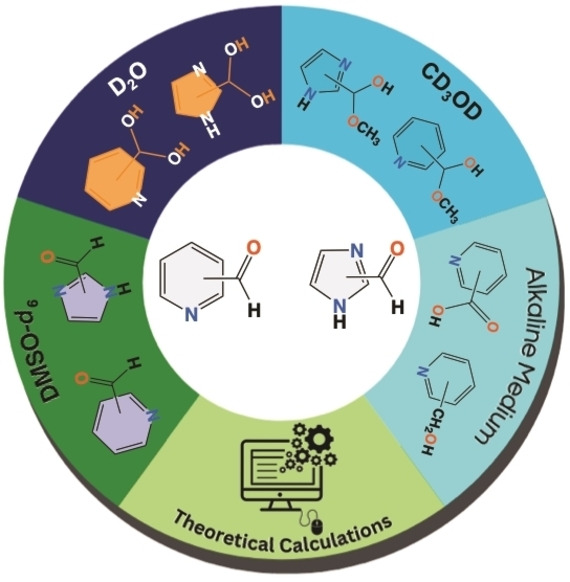

## Introduction

Nucleophilic addition reactions to carbonyl groups (of ketones and aldehydes)^[1]^ have been intensively studied because of their importance in the obtention of a wide variety of products such as *gem*‐diols (or geminal diols), alcohols, hemiacetals, imines, semicarbazones and α‐hydroxysilanes.[^2^, ^3^, ^4^] These compounds are key building blocks in the synthesis of pharmaceutical drugs, biologically active molecules, metal complexes and supramolecular structures.[^5^, ^6^, ^7^, ^8^, ^9^, ^10^] Particularly, *gem‐*diols and hemiacetals, which are obtained by the nucleophilic addition of water and alcohol molecules to a carbonyl group, respectively, are unstable and difficult to isolate because they easily revert to the original carbonyl compound.[^11^, ^12^, ^13^, ^14^, ^15^, ^16^, ^17^, ^18^, ^19^] In general, they are found as intermediates in key chemical reactions, such as those taking place at the active site of an enzyme[^20^, ^21^] or in the obtention of a product of interest such as the critical *gem*‐diol intermediate for anodic hydrogen production using 5‐hydroxymethylfurfural.^[22]^

**Scheme 1:**
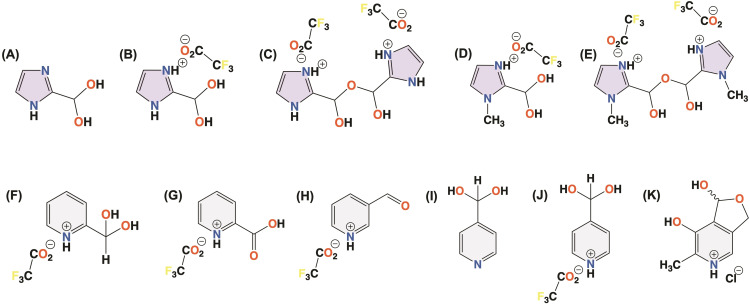
Neutral (A) and the trifluoroacetate derivatives for the *gem*‐diol (B) and the bis‐hemiacetal forms (C) of 2‐imidazolecarboxaldehyde. Trifluoroacetate derivatives of the *gem*‐diol (D) and the bis‐hemiacetal forms (E) of the *N*‐methyl‐2‐imidazolecarboxaldehyde. Trifluoroacetate derivatives of the *gem*‐diol (F), carboxylic acid (G) and aldehyde forms (H) for the 2‐pyridinecarboxaldehyde. Neutral *gem*‐diol (I) and trifluoroacetate derivatives of the *gem*‐diol form (J) of the 4‐pyridinecarboxaldehyde. Cyclic hemiacetal form (K) of the pyridoxal hydrochloride.

As benzaldehydes, pyridine‐ and imidazole‐carboxaldehydes undergo addition reactions with a wide variety of nucleophiles. These compounds and their derivatives have aroused great interest due to their many applications in organic synthesis, coordination chemistry, polymeric chemistry, and catalysis; due to the reactivity of both the carbonyl group and the nitrogen heteroatom.[^17^, ^23^, ^24^, ^25^, ^26^] For heterocyclic compounds, the electronic structure of the ring significantly influences the reactivity of the carbonyl group present at different positions. In pyridines, positions 2, 4, and 6 exhibit lower electron density due to the electron‐withdrawing effect of the nitrogen atom. In imidazoles, this effect is predominantly observed at position 2, which is between a pyridine‐ and a pyrrolic‐like nitrogens. In a previous work, the susceptibility of pyridinecarboxaldehydes to the nucleophilic addition of water and water‐trifluoroacetic acid (TFA) was analyzed in solution and *solid‐state* NMR, and single‐crystal X‐ray diffraction techniques. The hydration studies indicated that the position of the carbonyl group was crucial to stabilize the *gem*‐diol form, therefore allowing its isolation. The hydration of pyridinecarboxaldehyde compounds was favored by the electron‐withdrawing character of the aromatic system at positions 2 and 4, where the *gem*‐diol moiety was particularly stable.^[27]^ In particular, the *gem*‐diol form could be isolated as a trifluoroacetate derivative in those positions. However, 3‐pyridinecarboxaldehyde only rendered the carbonyl form in the solid state even as a trifluoroacetate. Particularly, the neutral solid *gem*‐diol form for the 4‐pyridinecarboxaldehyde could be directly isolated by reaction with water, during which the *gem*‐diol precipitates. Moreover, the isomers substituted with electron withdrawing groups increased the hydration degree when compared to those with electron donor substituents. As for imidazolecarboxaldehyde, the same analyses demonstrated a major reactivity at the position 2 in comparison with position 4.[^28^, ^29^] Scheme 1 shows some of the *gem*‐diol and bis‐hemiacetal forms generated from 2‐imidazolecarboxaldehyde,[^28^, ^29^] *N*‐methyl‐2‐imidazolecarboxaldehyde,^[28]^ pyridinecarboxaldehyde and the pyridoxal molecules.[^27^, ^30^] Furthermore, the chemical conversion of the carbonyl group in 4‐pyridinecarboxaldehyde or *N*‐methylimidazole‐2‐carboxaldehyde ligands in copper(II) complexes to the corresponding *ortho* ester and hemiacetal moieties^[24]^ or *gem*‐diol forms,^[25]^ respectively were demonstrated in the crystalline state.

This work analyzes the influence of the chemical environment, mainly as regards the type of solvent (protic or aprotic) and the medium acidity, in the nucleophilic addition of water or methanol molecules to pyridinecarboxaldehyde and imidazolecarboxaldehyde isomers. The study aims to study the susceptibility for the generation of the *gem*‐diol or hemiacetal forms of the 4‐, 3‐, and 2‐pyridinecarboxaldehydes; 4‐, 2‐imidazolecarboxaldehydes, and *N*‐methyl‐2‐imidazolecarboxaldehyde using NMR and theoretical calculations.

## Results and Discussion

### *Gem*‐Diol and Hemiacetal Generation in Pyridinecarboxaldehydes

The three pyridine isomers, 2‐pyridinecarboxaldehyde (**A_2_**), 3‐pyridinecarboxaldehyde (**A_3_**) and 4‐pyridinecarboxaldehyde (**A_4_**) were compared for the hydrate formation in D_2_O and DMSO‐*d*_6_, and in presence of TFA in a relation of 99 : 1 (solvent: TFA). The equilibrium of the *gem*‐diol and carbonyl form in solution was studied directly by NMR. Also, the hemiacetal formation was conducted in CD_3_OD under different conditions. Particularly, the integration of each ^1^H‐NMR signal for the *gem*‐diol/hemiacetal (δ^1^H=5.0–6.50 ppm) and/or the aldehyde groups (δ^1^H=9.10–10.50 ppm) allowed obtaining the relative content in terms of each functional moiety in the different media. Besides, the carboxylic acid and/or hemiacetal forms can be identified from the unequivocal assignment of the NMR signals using 2D‐HSQC and 2D‐HMBC experiments as described.[^27^, ^30^] Noteworthy, these hydration studies for the *gem*‐diol generation in the different carbonyl compounds were done using D_2_O or the remaining water content present in the DMSO‐*d*_6_ NMR solvent. The observed δ^1^H was dependent on the position of the aldehyde group in the pyridine ring (Supporting Information).

**Figure 1:**
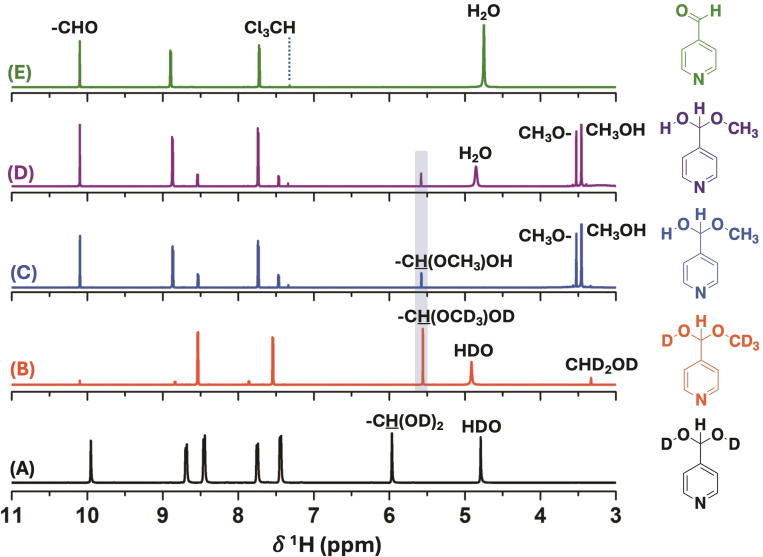
^1^H‐NMR spectra for **A_4_** in the presence of D_2_O (A), CD_3_OD (B), CDCl_3_ (with 20 μL of CH_3_OH) (C), CDCl_3_ (with 20 μL of CH_3_OH and 20 μL of H_2_O) (D) and CDCl_3_ (with 20 μL of H_2_O) (E). The final volume in all the NMR tubes was 500 μL.

The *gem*‐diol was only observed in D_2_O solution in equilibrium with the aldehyde form (Figure 1A and Table 1). The addition of water to the aldehyde group was not observed in the ^1^H‐NMR experiments in CD_3_OD due to the low amount of water present in the 4‐pyridinecarboxaldehyde reagent (∼10 mg H_2_O per 500 μL of aldehyde), and the higher nucleophilicity of methanol in comparison with water (Figure 1B).^[31]^ Special attention must be paid to the correct assignment of the *gem*‐diol against the hemiacetal form. Potential misassignments may occur due to the use of perdeuterated methanol as a solvent, which hinders the detection of the ‐OCHD_2_ group in both ^1^H‐ and ^13^C‐NMR spectra. Also, different NMR experiments were conducted for **A_4_** to evidence the formation of the hemiacetal derivative with methanol in CDCl_3_ (Figure 1). The ^1^H‐NMR spectrum of **A_4_** in CDCl_3_ with the addition of water (4 %) did not show the *gem*‐diol generation (Figure 1E), however, the hemiacetal compound was confirmed after the addition of methanol (4 %) in CDCl_3_ (Figure 1C). Furthermore, the addition of water to this last NMR experiment did not affect the hemiacetal content (30 %) nor showed the evidence of *gem*‐diol generation (Figure 1D). Remarkably, the hemiacetal content of **A_4_** in CD_3_OD and TFA:CD_3_OD was 95 % (with 5 % of aldehyde form) and 97 % (with 3 % of carboxylic acid form), respectively, showing the higher reactivity of the aldehyde group in methanol than in water (Figure 1, S5 and Table 1).

**Table 1:** Relative percentages of *gem‐*diol (GD), aldehyde (AL), hemiacetal (HA) and carboxylic acid (CA) forms of each pyridinecarboxaldehyde compound obtained from the ^1^H‐NMR spectra under different experimental conditions.

^1^H‐NMR												
	A_2_				A_3_				A_4_			
Experimental condition	GD	AL	HA	CA	GD	AL	HA	CA	GD	AL	HA	CA
D_2_O	30	61	–	9	10	90	–	–	48	52	–	–
D_2_O/TFA	100	–	–	–	85	15	–	–	99	1	–	–
CD_3_OD	–	19	68	13	–	27^[a]^	72^[a]^	–	–	5	95	–
CD_3_OD/TFA	–	2^[b]^	85^[b]^	13^[b]^	–	31	68	1	–	3	97	–
DMSO‐*d*_6_	*nd* ^[b]^				–	100	–	–	–	100	–	–
DMSO‐*d*_6_/TFA	*nd* ^[b]^				3	97	–	–	1	99	–	–

[a] 1 % acetal. [b] Particularly, it is very difficult to analyze this sample due to its decomposition and/or chemical reactions with TFA.^[30]^

Table 1 shows a comparison between the hydrate/hemiacetal formation and the remaining carbonyl form for each compound. It is expected that the carbonyl group at position 3 will be less activated for the nucleophilic addition, as compared to positions 2 and 4. For the pyridinecarboxaldehyde isomers, the ^1^H‐NMR spectra obtained under neutral conditions showed the presence of the *gem*‐diol form only in D_2_O and the hemiacetal in CD_3_OD, while in DMSO‐*d_6_* the isomers were only in the carbonyl form. Particularly, the percentage of hemiacetal generation in CD_3_OD was much higher when compared to that obtained for the *gem*‐diol in water (Table 1). The difference was particularly notable in the case of compound **A_3_**, considering that positions 2 and 4 of the pyridine ring are electro‐deficient, as compared to position 3 due to the electron‐withdrawing effect of the nitrogen atom.

**A_2_** and **A_4_** compounds led to the formation of *gem*‐diol in D_2_O, but in CD_3_OD an increase in the formation of pyridinecarboxylic acid was detected for **A_2_**. This denotes the high reactivity of this position since the hemiacetal form usually undergoes oxidization to the acid form.^[32]^ Different single‐crystals were isolated for 2‐pyridinecarboxylic acid when 2‐pyridinecarboxaldehyde molecules were incubated in acidic solutions (TFA or HCl solutions) for the isolation of the *gem*‐diol forms, as reported previously.^[30]^ However, it is worth mentioning that the bulk solid materials based on both crystalline and amorphous forms were analyzed by NMR in D_2_O. In this experiment, a mixture of the *gem*‐diol, the hemiacetal, and the carboxylic acid molecules was detected. Notably, the incubation of the trifluoroacetate of **A_3_** in the presence of ethanol, allowed obtaining the carboxylic acid form of **A_3_** (**CA_3_**) in the solid state, which was suitable for its study by single‐crystal X‐ray crystallography (CCDC 236209^[33]^ and Figures S65–S66). The results of the crystallographic study showed the evolution of the aldehyde group to the carboxylic acid moiety, which was indicative of the low stability of the generated hemiacetal form, as the trifluoroacetate derivative which evolved to the corresponding carboxylic acid forms in ethanol. This single‐crystal structure has previously been reported, but in that case, the trifluoroacetate derivative has been synthesized from the corresponding nicotinic acid and TFA (1 : 1).^[34]^

**Scheme 2:**
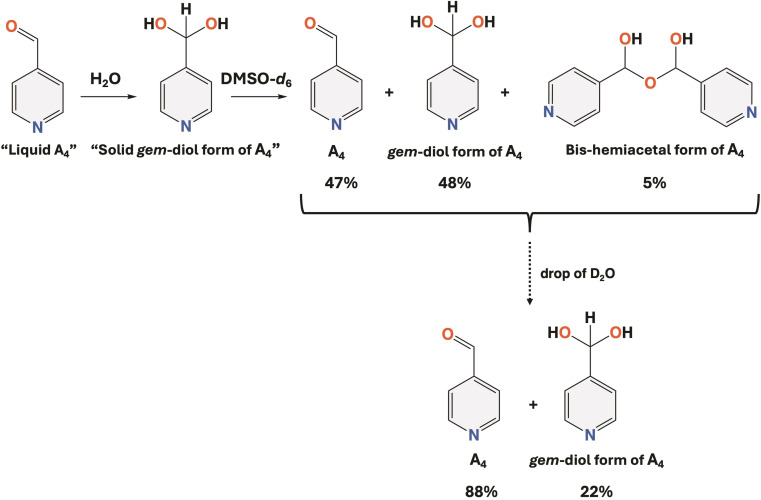
Chemical conversion of the solid *gem*‐diol form of **A_4_** in DMSO‐*d*_6_.

Furthermore, the solid sample of the neutral *gem*‐diol of **A_4_** (synthesized from **A_4_** in water)[^27^, ^35^] was dissolved in DMSO‐*d*_6_, showing that 47 % of the mixture remained as the hydrated form, while the rest either reverted to the parent aldehyde (49 %) or evolved to the bis‐hemiacetal structures (4 %) (Scheme 2). By adding a drop of D_2_O to the NMR tube, the bis‐hemiacetal content was depleted, and the *gem*‐diol increased its percentage to 53 % (Scheme 2 and Figure S13). These results evidenced that the stability of the *gem*‐diol or hemiacetal form is extremely dependent on the solvent used.

The results obtained at low pH in the presence of TFA indicated a high content of the *gem‐*diol/hemiacetal forms in the corresponding solvents (Table 1), evidencing that the protonation of the pyridine ring increased both the electro‐attracting effect of the nitrogen atom and the reactivity of the aldehyde group, in parallel with the transient protonation of the carbonyl group.^[36]^

When aprotic solvents, as DMSO‐*d*_6_, were used, the hydrated form was either observed in a very low proportion or was not observed at all, even in the presence of TFA due to the low water content (Table 1).[^37^, ^38^]

**Figure 2:**
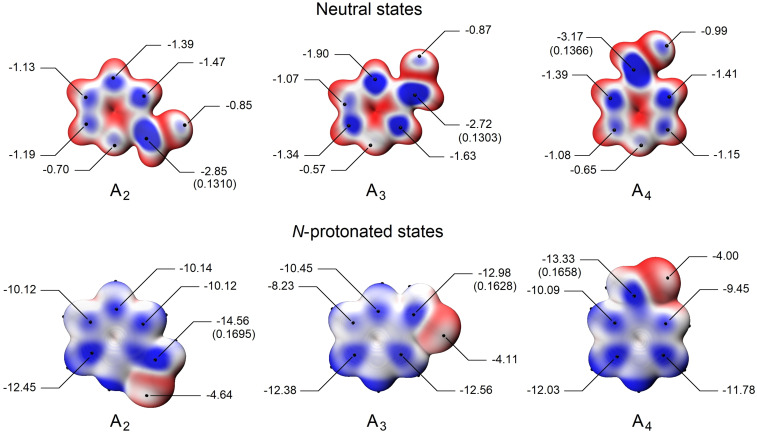
Computed E_S_(r) values for compounds **A_2_**, **A_3_** and **A_4_** at the 0.004 au isodensity surface for neutral (upper panel) and *N*‐protonated (lower panel) species, considered *in vacuo*. Images coloured according to a red‐to‐blue scale, being the blue regions the ones with the lowest E_S_(r) values. The points indicated by lines represent the lowest values on the isodensity surface (E_S,min_), expressed in eV units. The values indicated in brackets correspond to computed Hirshfeld partial charges on the respective reactive center.

To further study the reactivity profile of pyridinecarboxaldehydes against weak nucleophiles such as water or methanol, we used molecular modelling techniques based on Density Functional Theory (DFT) to model the structure and molecular properties of compounds **A_2_**, **A_3_** and **A_4_**. In this context, each compound was initially considered to be in a neutral state and was modelled as a conformational ensemble by applying the CREST+CENSO strategy. Next, taking the lowest energy conformer as a representative structure of each cluster, two independent computational simulation methodologies were applied for the estimation of the relative electrophilicity of each carbonyl group: the Hirshfeld charge distribution and the local electron attachment energy (LEAE).^[39]^ When the Hirshfeld charges were calculated *in vacuo*, the carbon atom of the carbonyl group at positions 2 and 4 of the pyridine ring showed the highest positive charge densities. In the absence of net charge on the pyridine ring, the order of reactivity based on the calculated charge distribution was **A_4_** >**A_2_** >**A_3_** (Figure 2). According to the computed Hirshfeld charges, however, the protonation of the pyridine nitrogen changed the predicted order of reactivity to **A_2_** >**A_4_** >**A_3_**. Since these results only explain the experimental profile observed in neutral conditions, the calculations were repeated with the inclusion of implicit solvents. Regardless of the solvent used (water, methanol, or DMSO), the calculated Hirshfeld charge distribution predicted the reactivity of the carbonyl group of the neutral species as **A_4_** >**A_3_** >**A_2_**. The protonation of the pyridine nitrogen atom in the three isomers changed this order to **A_2_** >**A_4_** >**A_3_**.

**Figure 3:**
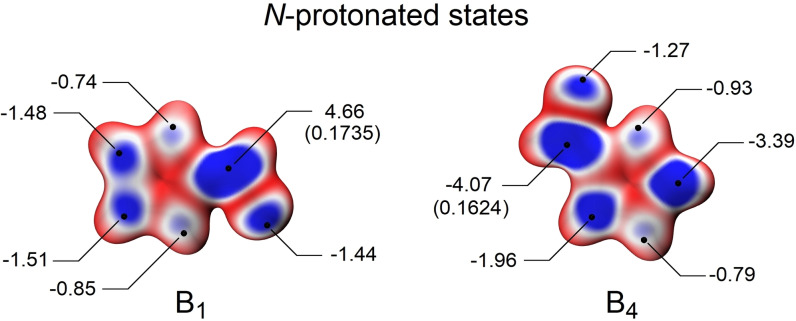
Computed E_S_(r) values for compounds **B_1_** and **B_4_** at the 0.004 au isodensity surface for *N*‐protonated species, considered in methanol media. Images coloured according to a red‐to‐blue scale, being the blue regions the ones with the lowest E_S_(r) values. The points indicated by lines represent the lowest values on the isodensity surface (E_S,min_), expressed in eV units. The values indicated in brackets correspond to computed Hirshfeld partial charges on the respective reactive center.

Therefore, *in vacuo* calculations were suitable to predict the order of reactivity in neutral conditions for the three compounds, and the *N*‐protonation observed in an acidic medium. In this context, the LEAE methodology,^[39]^ which is recommended for the study of nucleophilic reactions, was applied in further theoretical calculations. This methodology allows predicting the reactivity of electrophilic centers for the nucleophilic attack based on the value adopted by the electronic descriptor E_S,min_. The latter parameter indicates that the lower the value reached, the higher the electrophilic character of the reactive center, and therefore, the higher susceptibility to nucleophilic attack. In the absence of net charge on the pyridine ring, the order of reactivity of the carbonyl carbon in the nucleophilic reaction, based on the E_S,min_ values calculated *in vacuo*, was **A_4_** >**A_2_** >**A_3_** (Figure 3). However, the protonation of the nitrogen atom changed the order of reactivity to **A_2_** >**A_4_** >**A_3_**, thus repeating the profile predicted by the Hirshfeld charges. The E_S,min_ calculated in the presence of any of the three solvents for the neutral forms was **A_4_** ≥**A_2_** >**A_3_**. The calculated E_S,min_ values for species that are protonated in the nitrogen atom allowed assigning the following order of reactivity with either any solvent or *in vacuo* as **A_2_** >**A_4_** >**A_3_**, which is the same order as that obtained through the calculations of the Hirshfeld charges.

Both methodologies predicted the order of experimental reactivity *in vacuo* under neutral conditions. However, the LEAE methodology also achieved accurate predictions in the presence of different solvents. Under acidic conditions, both computational methods predicted the order of reactivity observed for the *gem*‐diol and hemiacetal generation when the species considered were *N*‐protonated and *in vacuo* or in the presence of the indicated solvents.

### *Gem*‐Diol and Hemiacetal Generation in Imidazolecarboxaldehydes

The ^1^H‐NMR spectra of the three imidazolecarboxaldehyde derivatives, 2‐imidazolecarboxaldehyde (**B_1_**), *N*‐methyl‐2‐imidazolecarboxaldehyde (**B_2_**) and 4‐imidazolecarboxaldehyde (**B_4_**), under neutral conditions, indicated that the hydrate form is not generally present in D_2_O and that the hemiacetal form is generated in CD_3_OD (Table 2). The three compounds exhibited a higher susceptibility to the nucleophilic addition of CD_3_OD molecules, as compared to D_2_O, a solvent where only **B_2_** shows 2 % of *gem*‐diol content. Moreover, the ^1^H‐NMR spectra of the isomers in DMSO‐*d*_6_ only showed the presence of the aldehyde form (Table 2).

**Table 2:** Relative percentages of *gem*‐diol (GD), aldehyde (AL) and hemiacetal (HA) forms of each imidazolecarboxaldehyde obtained from the ^1^H‐NMR spectra under different experimental conditions.

^1^H‐NMR									
	B_1_			B_2_			B_4_		
Experimental condition	GD	AL	HA	GD	AL	HA	GD	AL	HA
D_2_O	–	100^[29]^	–	2^[28]^	98^[28]^	–	–	100	–
D_2_O/TFA	100^[29]^	–	–	100^[28]^	–	–	36	64	–
CD_3_OD	–	55	45	–	64	36	–	96	4
CD_3_OD/TFA	–	–	100	–	34	66	–	15	85
DMSO‐*d*_6_	–	100	–	–	100	–	–	100	–
DMSO‐*d*_6_/TFA	55	45	–	33	67	–	–	100	–

The ^1^H‐NMR spectra of **B_1_**, **B_2_** and **B_4_** dissolved in D_2_O or CD_3_OD with 1 % of TFA evidenced an increase in the reactivity towards the nucleophilic addition of water or methanol molecules. In previous reports, the dissolution of compound **B_2_** in D_2_O/TFA induced the generation of both the *gem*‐diol and the bis‐hemiacetal molecules.^[28]^ Furthermore, the formation of the hemiacetal of the corresponding imidazolecarboxaldehyde was enhanced in CD_3_OD under acidic conditions (Table 2).

Although one of the nitrogen atoms of the imidazole ring has pyridine characteristics, the behavior of these heterocycles differs from that of the pyridinecarboxaldehydes in solution.^[29]^ On the other hand, as with pyridinecarboxaldehydes, the presence of the acid medium favors the addition of weak nucleophiles. This could be partially explained in terms of the Hirshfeld charges (Figure 3), because these cannot explain the observed reactivity order when **B_2_** is considered. Nevertheless, computed charges only predict the higher reactivity of **B_1_** relative to **B_4_**, in acidic medium for all the solvents studied, as well as *in vacuo*, when the mono protonated species are having into account.

The LEAE methodology applied to imidazole compounds as neutral forms rendered E_S,min_ values that predicted a reactivity that was higher for **B_1_** than for **B_4_**, regardless of the reaction medium. However, the E_S,min_ value could not explain the reactivity order corresponding to **B_2_**. Using an implicit solvent for protonated imidazole derivatives, the experimental order of reactivity observed for compounds **B_1_** and **B_4_** was consistent with the calculated E_Smin_ (Figure 3).

### Pyridine‐ and Imidazole‐Carboxaldehydes in Alkaline Medium

The reactivity of pyridine‐ and imidazole‐carboxaldehydes was evaluated in alkaline medium using the same solvents and the results are summarized in Tables 3 and 4. For imidazole derivatives (**B_1_**, **B_2_**, and **B_4_**) in 0.1 M NaOH in D_2_O or DMSO‐*d*_6_, the aldehyde group was predominantly observed as it was reported by Grimmet.^[40]^ An exception occurred in 0.1 M NaOH in methanol, where **B_4_** was converted to the hydroxymethyl and the sodium carboxylate moieties, products of the Cannizzaro reaction,[^11^, ^41^] in which two molecules of a nonenolizable aldehyde are disproportionated by a base to produce a carboxylic acid and a primary alcohol. In this case, the high content of carboxylic acid may be due to some degree of oxidation of the hemiacetal derivatives in methanol (Table 2). Likewise, **B_4_** showed a minor content of the hemiacetal form in 0.1 M NaOH in methanol. Particularly, those isomers that do not have the methyl group in the pyrrolic nitrogen remain in the carbonyl form in a basic medium.

**Table 3:** Relative percentages of *gem‐*diol (GD), aldehyde (AL), hemiacetal (HA), sodium carboxylate (SC) and hydroxymethyl (ALC) forms of each imidazolecarboxaldehyde compound obtained from the ^1^H‐NMR spectra under different experimental conditions.

	^1^H‐NMR									
	B_1_		B_2_					B_4_		
Experimental condition	AL	HA	GD	AL	HA	SC	ALC	GD	AL	HA
0.1 M NaOH in D_2_O	100^[29]^	–	–	100	–	–	–	–	100	–
0.1 M NaOH in CD_3_OD	100	–	–	4	–	60	36	–	96	4
0.1 M NaOH in DMSO‐*d*_6_	100	–	–	100	–	–	–	–	100	–

**Table 4:** Relative percentages of *gem‐*diol (GD), aldehyde (AL), hemiacetal (HA), sodium carboxylate (SC) and hydroxymethyl (ALC) forms of each pyridinecarboxaldehyde compound obtained from the ^1^H‐NMR spectra under different experimental conditions.

	^1^H‐NMR														
	A_2_					A_3_					A_4_				
Experimental condition	GD	AL	HA	SC	ALC	GD	AL	HA	SC	ALC	GD	AL	HA	SC	ALC
0.1 M NaOH in D_2_O	21	48	–	21	10	70	12	–	9	9	47	43	‐	5	5
0.4 M NaOH in D_2_O	*nd* ^[a]^					–	–	–	51	49	7	7	‐	43	43
0.1 M NaOH in CD_3_OD	*nd* ^[a]^					–	26	74	–	–	–	4	96	–	–
0.4 M NaOH in CD_3_OD	*nd* ^[a]^					*nd* ^[a]^					–	–	81	14	5
0.1 M NaOH in DMSO‐*d*_6_	*nd* ^[a]^					–	100	–	–	–	18	82	–	–	–
0.4 M NaOH in DMSO‐*d*_6_	*nd* ^[a]^					*nd* ^[a]^					40	60	–	–	–

[a] Particularly, it is very difficult to analyze this sample due to its decomposition and chemical reactions.

On the other hand, the pyridinecarboxaldehyde isomers displayed higher reactivity in the Cannizzaro reaction than the imidazolecarboxaldehydes, influenced by the position of the aldehyde group and the solvent. In this sense, 0.1 or 0.4 M NaOH solutions were studied in the different solvents (Table 4).

For **A_3_**, the Cannizzaro reaction products were favored at both 0.1 and 0.4 M NaOH solutions in D_2_O. However, 70 % of the *gem*‐diol for **A_3_** was obtained at 0.1 M NaOH. With 0.4 M NaOH concentration the reaction was quantitative with the hydroxymethyl and sodium carboxylate derivatives as the main products. Furthermore, in the last condition the aldehyde or *gem*‐diol forms were not observed. In 0.1 M NaOH in CD_3_OD, only the hemiacetal and aldehyde forms were detected with no acetal or Cannizzaro products. In DMSO‐*d*_6_, **A_3_** remained solely in its aldehyde form (Table 4).

Besides, **A_4_** shows greater susceptibility to the Cannizzaro reaction in both water and methanol solvents, being quantitative higher in water. In methanol, the reaction condition requires higher NaOH concentrations. In DMSO‐*d*_6_, only the *gem*‐diol and aldehyde forms were detected. Remarkably, 47 % of *gem*‐diol form was generated with 0.1 M NaOH in D_2_O with a significant reduction to 7 % with 0.4 M NaOH in D_2_O.

Finally, **A_2_** displays the products of the Cannizzaro in a similar content than **A_3_** and **A_4_** in 0.1 M NaOH in D_2_O, but with a lower evolution to the *gem*‐diol generation. Also, some degree of oxidation to the carboxylic acid form is observed (Table 4). Particularly, no other NMR studies were conducted for **A_2_** due to its higher tendency to render degradation products, hindering the assignment of the NMR signals. In this sense, the analysis of **A_3_** at 0.4 M NaOH in methanol or DMSO‐*d*_6_ was also intricated due to multiple side products.

Regarding the *gem*‐diol generation, **A_3_** shows the higher reactivity to the hydrate formation in comparison with **A_4_** and **A_2_** in alkaline medium (Table 4). This higher percentage of hydration for **A_3_** was not expected in the context of this study, where both **A_2_** and **A_4_** compounds showed similar percentages of hydration than in D_2_O (Table 1).

## Conclusions

This work assessed the generation of the *gem*‐diol or hemiacetal forms derived from pyridine‐ and imidazolecarboxaldehyde derivatives, as well as its stability in various chemical environments in solution. The solution‐state NMR studies indicated that the position of the formyl group was essential to stabilize the *gem*‐diol or hemiacetal form.

Pyridinecarboxaldehydes showed a great dependance on protic solvents for the *gem*‐diol/hemiacetal generation as well as on the protonation state of pyridine ring.

The computed Hirshfeld charge distribution for the studied pyridinecarboxaldehydes was a good predictor of the order of experimental reactivity *in vacuo* under neutral conditions. The LEAE methodology was equally useful to predict reactivities in the presence of the different solvents. Both methodologies predicted the order observed when the species considered were *N*‐protonated in the presence of each solvent.

Particularly, the three pyridinecarboxaldehyde isomers showed greater reactivity to the methanol molecule addition in CD_3_OD in neutral conditions, relative to the behavior observed in water.

On the other hand, imidazolecarboxaldehydes showed much more dependance on the protonation state on the pyridine‐type nitrogen for the nucleophilic reaction. Notably, the hemiacetal generation was increased in CD_3_OD, even when the three compounds remained practically in the aldehyde form in D_2_O.

Computed Hirshfeld charges and E_S,min_ provided a limited explanation about the order of reactivity in acidic media for compounds **B_1_** and **B_4_** and the reactivity of compound **B_2_** could not be explained by the calculated parameters.

As for *gem*‐diol generation in alkaline medium, **A_3_** showed the highest reactivity to the hydrate formation compared with **A_4_** and **A_2_**. This unexpected result allows us to continue studying the behavior of this type of aldehyde derivatives in different aromatic heterocyclic compounds through theoretical calculations and experimental analysis.

Regarding the Cannizzaro reaction, the pyridinecarboxaldehydes were highly reactive than the imidazolecarboxaldehydes, influenced by the position of the aldehyde group and the solvent, being **A_3_** quantitative converted to the carboxylic acid and alcohol moieties in 0.4 M NaOH in D_2_O. For **A_4_** the reaction also took place, but the *gem*‐diol and aldehyde forms remained in low content.

Finally, the NMR spectroscopy was useful to analyze the evolution of the aldehyde to the *gem*‐diol/hemiacetal forms in a fast and simple way while allowing the evaluation of a wide range of solvents and acidity degree of the medium. Thus, this method is a very useful tool to evaluate the chemical composition in cases where the hydroxymethyl, carbonyl, *gem*‐diol, hemiacetal, acetal and oxidized forms coexist for a particular carbonyl compound with electron withdrawing character.

## Conflict of Interests

The authors declare no conflict of interest.

## Supporting information

As a service to our authors and readers, this journal provides supporting information supplied by the authors. Such materials are peer reviewed and may be re‐organized for online delivery, but are not copy‐edited or typeset. Technical support issues arising from supporting information (other than missing files) should be addressed to the authors.

Supporting Information

## Data Availability

The data that support the findings of this study are available in the supplementary material of this article.
