# Supplementary material for: Reactivity of Imidazole‐ and Pyridine‐Carboxaldehydes for Gem‐Diol and Hemiacetal Generation: Theoretical and Experimental Insights
Source: ChemistryOpen. 2025 Jan 5;14(8):e202400411. doi: 10.1002/open.202400411 (PMC12368889; doi:10.1002/open.202400411)
Supplement: Supplementary file 1 — Supporting Information [file OPEN-14-e202400411-s001.pdf]

ChemistryOpen

Supporting Information

Reactivity of Imidazole- and Pyridine-Carboxaldehydes for *Gem*-Diol and Hemiacetal Generation: Theoretical and Experimental Insights

Ayelén F. Crespi, Emiliano Barrionuevo, Gabriel Jasinski, Albertina G. Moglioni, Daniel Vega, and Juan M. Lázaro-Martínez*

Table of contents

Content	Page
1. Experimental Section	2
2. Solution-state NMR (Figures S1-S64)	3-50
3. Single-crystal X-ray diffraction results (Figures S65-S66; Tables S1-S4)	51-56

Experimental Section

Materials. 4-pyridinecarboxaldehyde (**A_4**, 98%), 3-pyridinecarboxaldehyde (**A_3**, 98%), 2-pyridinecarboxaldehyde (**A_2**, 98%), 4-imidazolecarboxaldehyde (**B_4**, 98%), *N*-methyl-2-imidazolecarboxaldehyde (**B_2**, 98%), trifluoroacetic acid (TFA, 99%), sodium hydroxide (NaOH, $\geq$ 97%), deuterium oxide (D_2O, 99.9 atom %D), methanol-*d_4* (CD_3OD, > 99.8 atom %D), and dimethylsulfoxide-*d_6* (DMSO-*d_6*, 99.8 atom %D) were purchased from Sigma Aldrich and used without further purification. The 2-imidazolecarboxaldehyde (**B_1**) compound was synthesized as in previous reports.[29,43]

NMR experiments. Nuclear Magnetic Resonance data was acquired with a Bruker Avance-III HD spectrometer equipped with a 14.1 T narrow bore magnet operating at Larmor frequencies of 600.09 MHz and 150.91 MHz for ^1H and ^{13}C, respectively. To determine the hydration of the carbonyl group, ~10 mg of each compound was dissolved in D_2O, CD_3OD or DMSO-*d_6*. Moreover, the compounds were dissolved in a solution of each solvent containing 1% of TFA or 1% of NaOH, respectively.

Theoretical calculations. All the pyridine- and imidazole-carboxaldehyde isomers, in the protonation states and the respective *gem*-diols and cationic homoconjugates, were constructed using GaussView 6.0[44] and initially optimized in Gaussian 16 A03[45] using a PM6 semi-empirical method. After a semi-empirical re-optimization of the geometries using the method GFN-xTB[46], each structure was processed using a workflow named CREST+CENSO[47,48] with the aim to model a Boltzmann conformer distribution at 298.15 K. The first step of this workflow is a conformational sampling using an iterative metadynamics algorithm (iMTD-GC) implemented in CREST using a threshold of 15 kcal mol^{-1} for the rejection of the high energy conformers (the rest of the options were set as default). Then the set of conformers obtained were evaluated in the three-stage procedure implemented in CENSO. The calculations implemented in CENSO stages were developed using ORCA 5.0.3.[49] and the energy threshold for this step was set to 12 kcal mol^{-1} for the rejection of the high energy conformations. In the first stage of CENSO, the electronic energies were estimated using a single point with a B97-D3(0)/def2-SV(P)+gCP DFT method. In the second stage, the molecular free energies were estimated using r^2-SCAN-3c/def2-mTZVPP for the estimation of the electronic contribution and hessian GFN2-xTB for the estimation of the respective thermostatistical contribution. In the final stage, the molecular geometries of each conformer were re-optimized using the double-hybrid functional DSD-BLYP-D3 with a def2-TZVPP basis set, also the free energy values (ΔG_{vacuum}) were computed, retaining only those conformations whose contribution was significant for the Boltzmann distribution (Boltzmann sum threshold et to 99.9%). The final geometries were used to calculate the free energy of solvation ($\Delta G_{solvation}$) using the M062X/6-31G(d) level of theory and the SMD solvation model.[50] Finally, the free energies for each conformer in solution were obtained by the sum of each free energy values computed in vacuum and each solvation energy:

$$\Delta G_{Solution} = \Delta G_{Vacuum} + \Delta G_{Solvation}$$

The values reported correspond to a Boltzmann-weighted sum of each the conformers obtained by this procedure. The conformations obtained after CREST+CENSO workflow for the aldehydes in neutral, N-protonated and N,O-diprotonated states were used for the calculation of the Hirschfeld charges and $E_{s\ min}$ values. These, were computed using Multiwfn 3.8.[51] The level of theory used for these calculations was B3LYP/6-311+G(d,p). The calculation of Hirshfeld charges and $E_{s\ min}$ values using implicit solvent conditions (water, methanol and DMSO) were implemented using CPCM solvation model.

Single-crystal X-ray crystallography studies. Single crystals (CCDC 2362091) were isolated by slow evaporation of ethanolic solution containing the trifluoroacetate derivative of 3-pyrdidinecarboxaldehyde as described previously.[27-29,35] Single-crystal X-ray diffraction data were collected at 294 K, using a Quest ECO diffractometer.

Solution-state NMR Experiments

The unequivocal ^{1}H-NMR spectra assignment of the compounds dissolved in D_2O and D_2O/TFA were previously reported.[27-29] In case of the experiments carried out in CD_3OD, DMSO-d_6 and basic media, the assignment was carried out employing ^{13}C-NMR, HSQC and HMBC experiments.

Figure S1. ^{1}H-NMR spectra of **A₄** in D_2O: 52% aldehyde and 48% *gem*-diol.

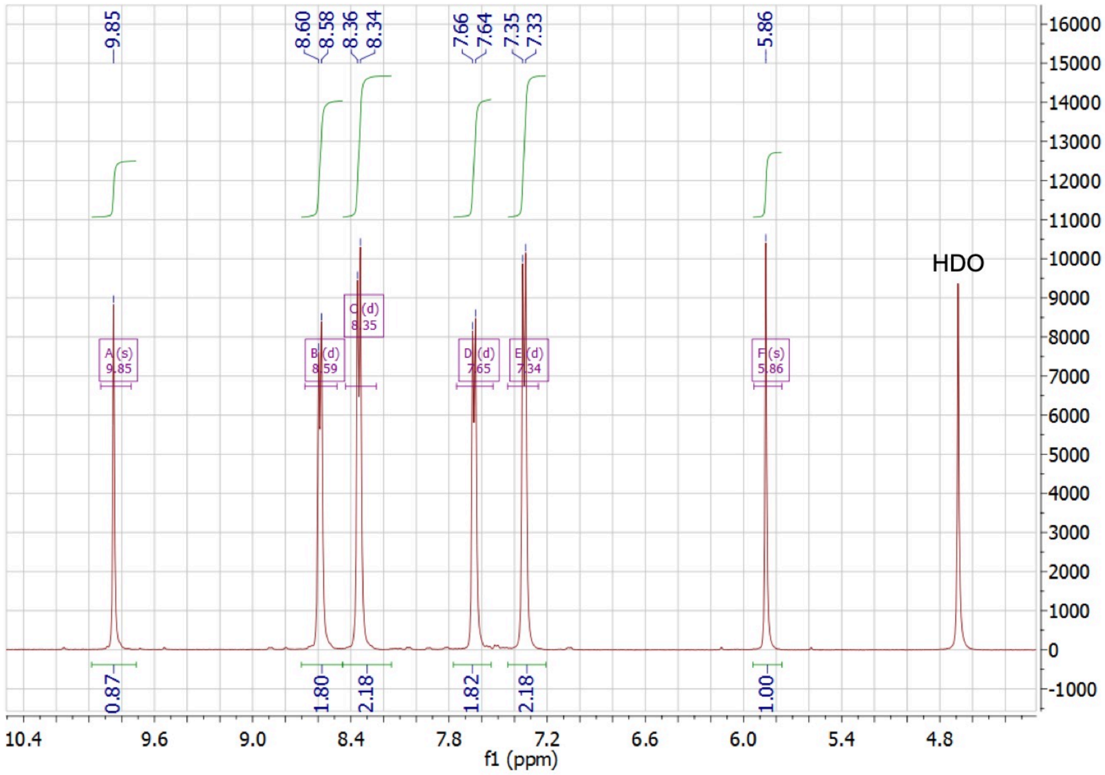

Aldehyde: δ 9.85 (s, -C**H**O), 8.59 (d, J = 5.3 Hz, $\mathbf{H_{2,6}}$), 7.65 (d, J = 5.4 Hz, $\mathbf{H_{3,5}}$). *gem*-diol: δ 8.35 (d, J = 5.5 Hz, $\mathbf{H_{2,6}}$), 7.34 (d, J = 5.4 Hz, $\mathbf{H_{3,5}}$), 5.86 (s, -C**H**(OH)$_2$).

Figure S2. ^{1}H-NMR spectra of **A_4** D_2O/TFA: 1% aldehyde and 99% *gem*-diol.

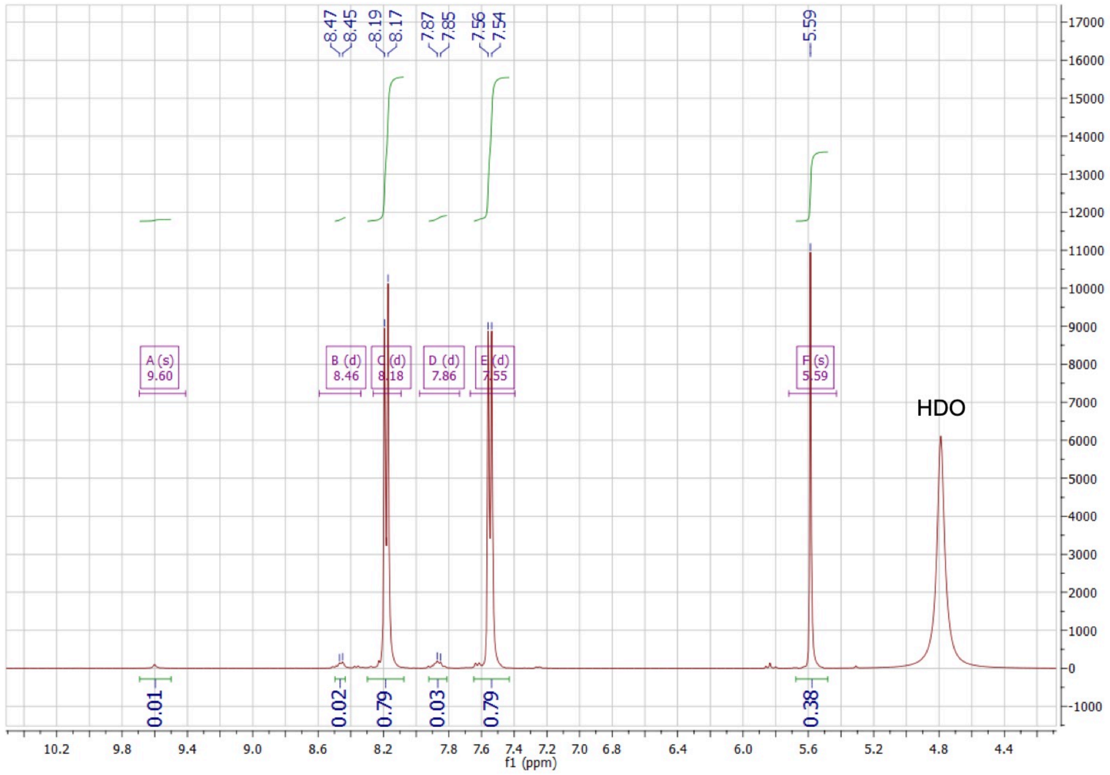

Aldehyde: δ 9.60 (s, -C**H**O), 8.46 (d, J = 5.3 Hz, **$H_{2,6}$**), 7.86 (d, J = 5.4 Hz, **$H_{3,5}$**); *gem*-diol: δ 8.18 (d, J = 5.5 Hz, **$H_{2,6}$**), 7.55 (d, J = 5.4 Hz, **$H_{3,5}$**), 5.59 (s, -C**H**$(OH)_2$).

Figure S3. ^{1}H-NMR spectra of **A_4** in D_2O/NaOH (0.1M): aldehyde 43%, *gem*-diol 47%, carboxylic acid 5% and Hydroxymethyl 5%.

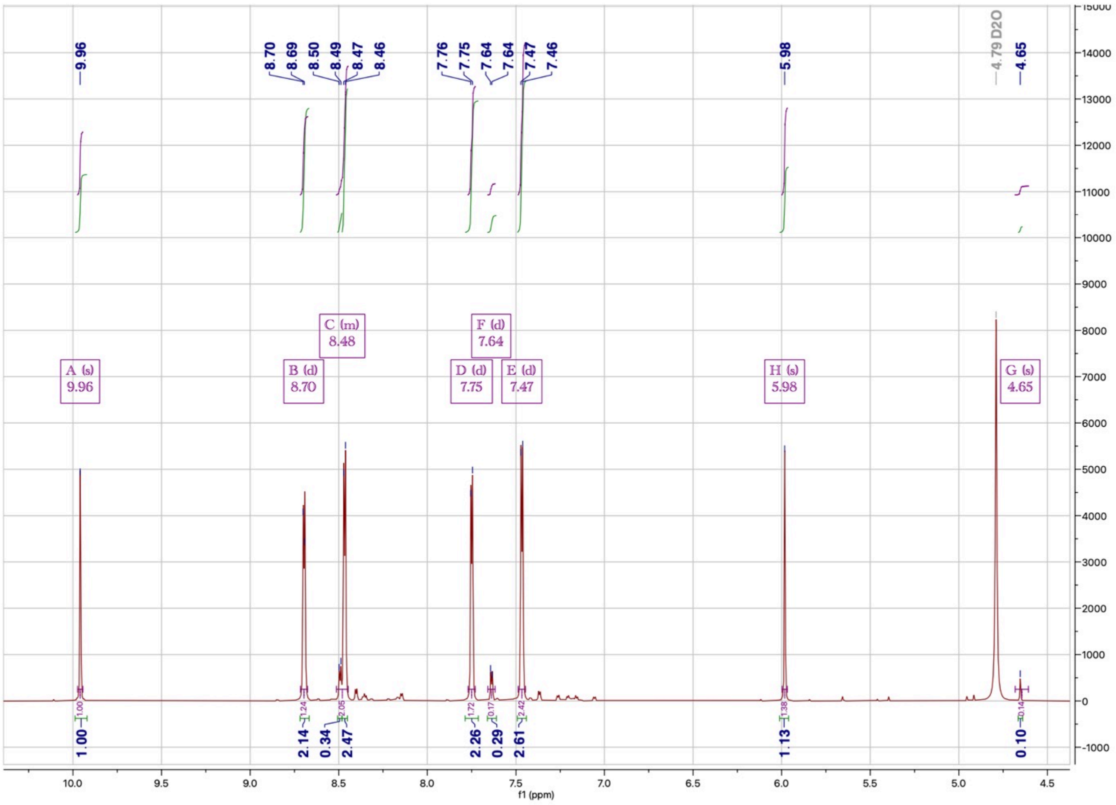

Aldehyde: δ 9.96 (s, -C**H**O), 8.70 (d, J = 5.3 Hz, **$H_{2,6}$**), 7.75 (d, J = 5.4 Hz, **$H_{3,5}$**). *gem*-diol: δ 8.51 – 8.45 (d, J = 5.5 Hz, **$H_{2,6}$**), 7.47 (d, J = 5.4 Hz, **$H_{3,5}$**), 5.59 (s, -C**H**$(OH)_2$).Carboxylic acid: 8.51 – 8.45 (m, **$H_{2,6}$**), 7.47 (d, *J* = 6.4 Hz, **$H_{3,5}$**), Hydroxymethyl: 8.51 – 8.45 (m, **$H_{2,6}$**), 7.64 (d, *J* = 4.5 Hz, **$H_{3,5}$**), 4.65 (s, -C**H_2**OH).

Figure S4. ^{13}C-NMR spectra of $\mathbf{A_4}$ in D_2O/NaOH (0.1M): aldehyde 43%, *gem*-diol 47%, carboxylic acid 5% and Hydroxymethyl 5%.

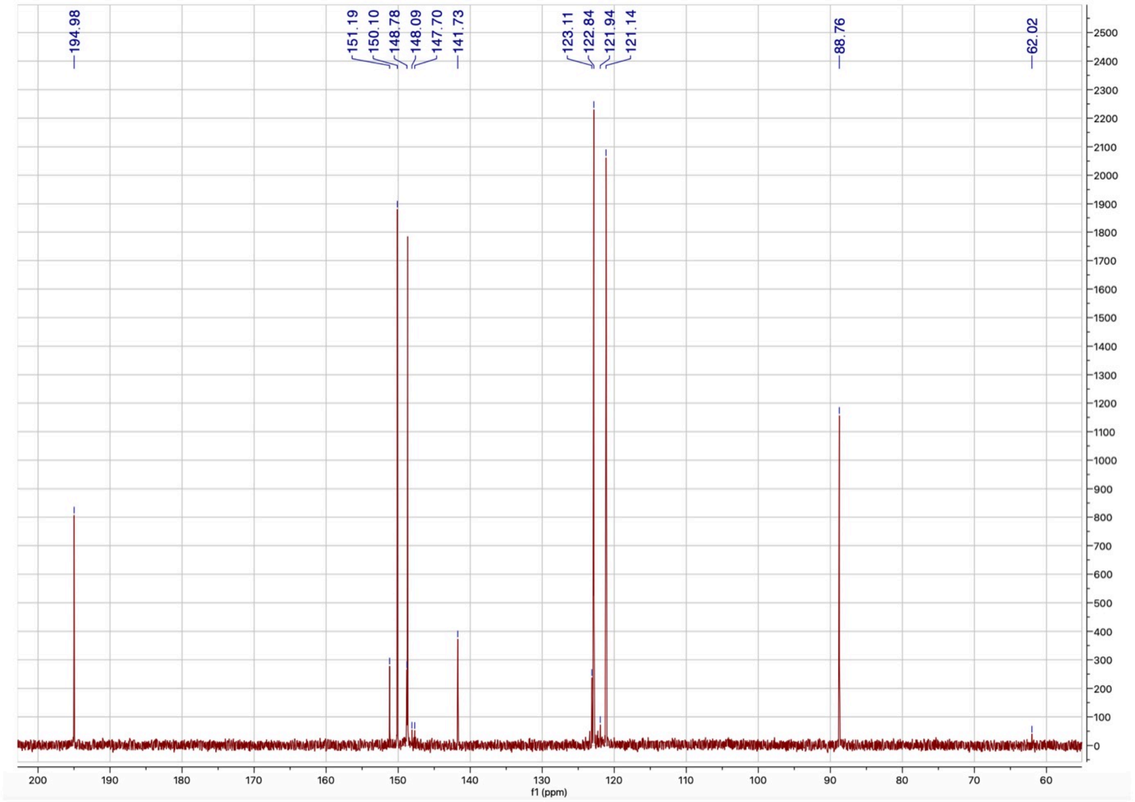

Aldehyde: δ 194.98 (-**C**HO), 151.19 ($\mathbf{C_4}$), 148.78 ($\mathbf{C_{2,6}}$), 121.14 ($\mathbf{C_{3,5}}$) *gem*-diol:150.10 ($\mathbf{C_{2,6}}$), 141.73 ($\mathbf{C_4}$), 122.84 ($\mathbf{C_{3,5}}$), 88.76 (-**C**H(OH)$_2$); carboxylic acid: 148.09 ($\mathbf{C_{2,6}}$); Hydroxymethyl: 147.70 ($\mathbf{C_{2,6}}$), 123.11 ($\mathbf{C_4}$), 121.94 ($\mathbf{C_{3,5}}$), 62.02 (-**C**H$_2$OH).

Figure S5. ^{1}H-NMR spectra of **A4** in CD_3OD: 5% aldehyde and 95% hemiacetal.

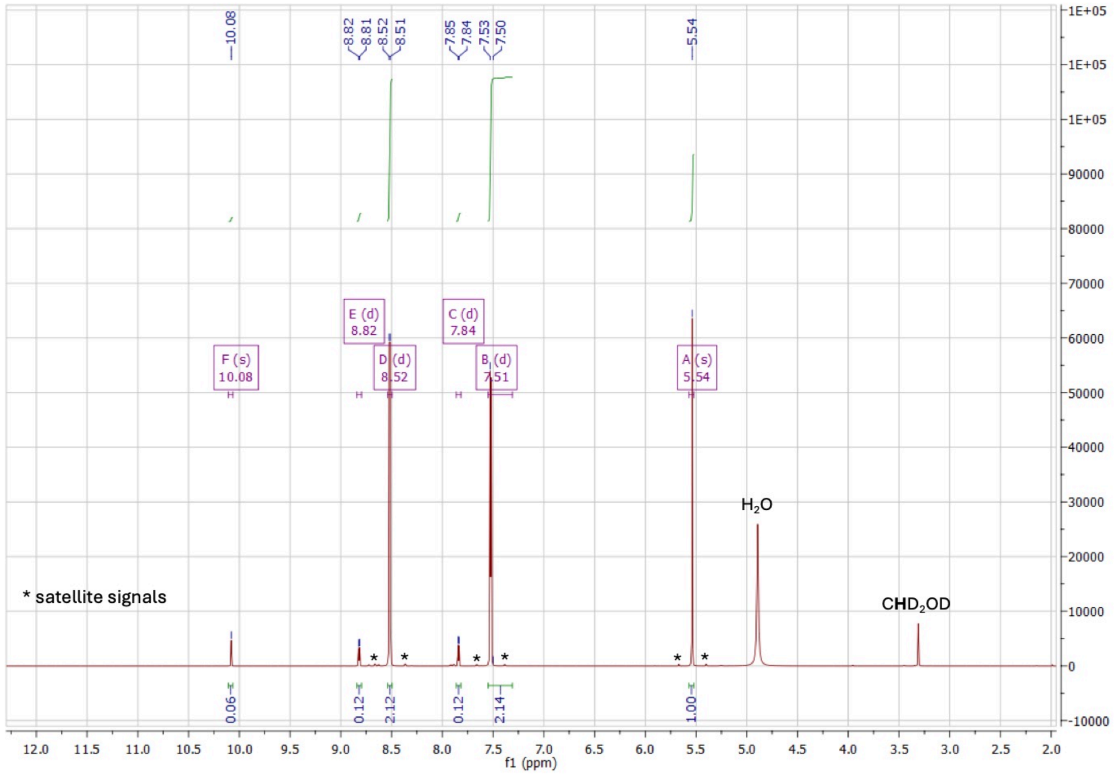

Aldehyde: δ 10.08 (s, -C**H**O), 8.82 (d, J = 5.9 Hz, **H**$_{2,6}$), 7.84 (d, J = 6.0 Hz, **H**$_{3,5}$); hemiacetal: 8.52 (d, J = 6.1 Hz, **H**$_{2,6}$), 7.51 (d, J = 6.1 Hz, **H**$_{3,5}$), 5.54 (s, -C**H**(OH)(OCD_3)).

Figure S6. ^{13}C-NMR spectra of **A4** in CD_3OD: 5% aldehyde and 95% hemiacetal.

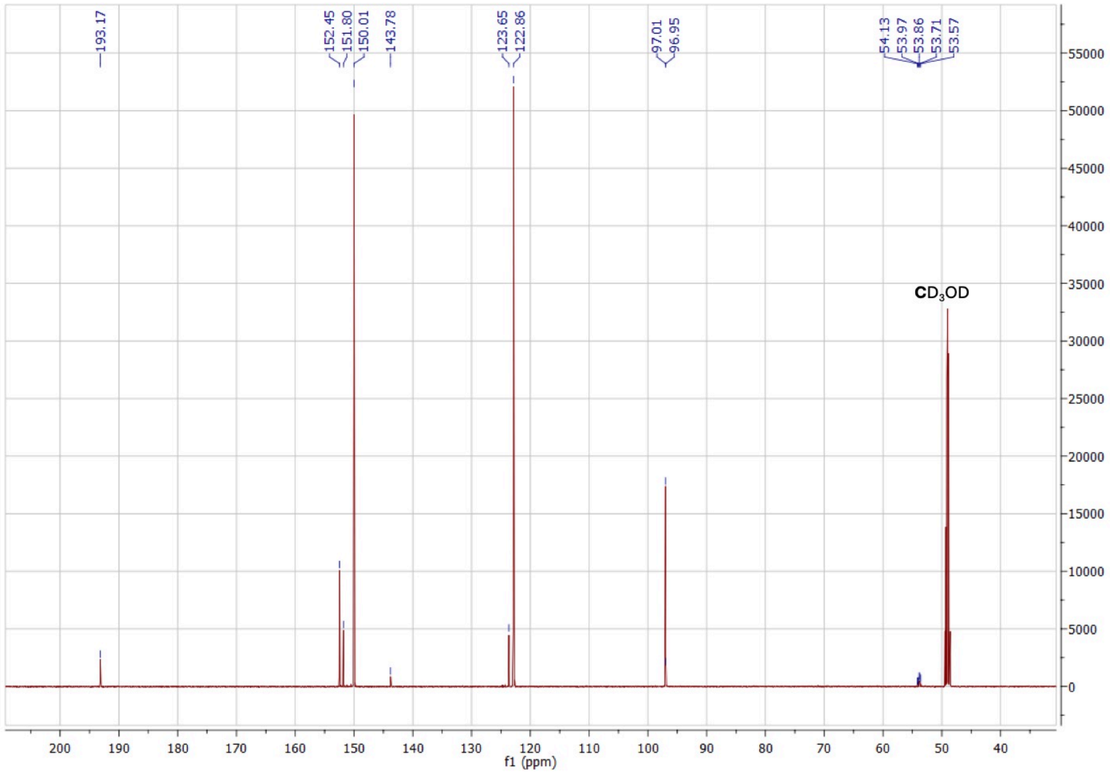

Aldehyde: δ 193.17 (s, -**C**HO), 151.80 (**C**$_{2,6}$), 143.78 (**C**$_4$), 123.65 (**C**$_{3,5}$); hemiacetal: 152.45 (**C**$_4$), 150.01 (**C**$_{2,6}$), 122.86 (**C**$_{3,5}$), 97.20 (-**C**H(OH)(OCD_3), 54.13-53.57 (-O**C**D_3).

Figure S7. ^{1}H-NMR spectra of **A_4** in CD_3OD/TFA: 97% hemiacetal and 3% aldehyde.

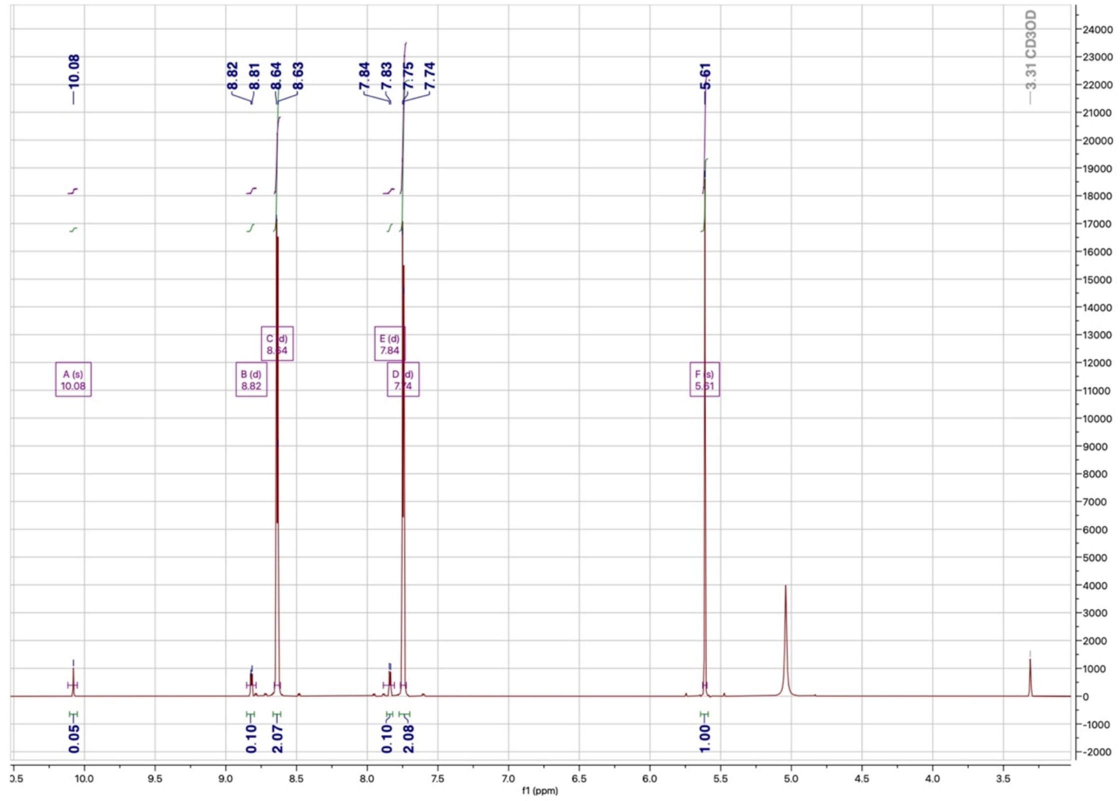

Aldehyde: δ 10.08 (s, -C**H**O), 8.82 (d, *J* = 4.5 Hz, **$H_{2,6}$**), 7.84 (d, *J* = 6.0 Hz, **$H_{3,5}$**); hemiacetal: 8.64 (d, *J* = 7.7 Hz, **$H_{2,6}$**), 7.74 (d, *J* = 6.8 Hz, **$H_{3,5}$**), 5.61 (s, -C**H**(OH)(OCD_3)).

Figure S8. ^{1}H-NMR spectra of **A_4** in CD_3OD/NaOH (0.1M): 96% hemiacetal and 4% aldehyde.

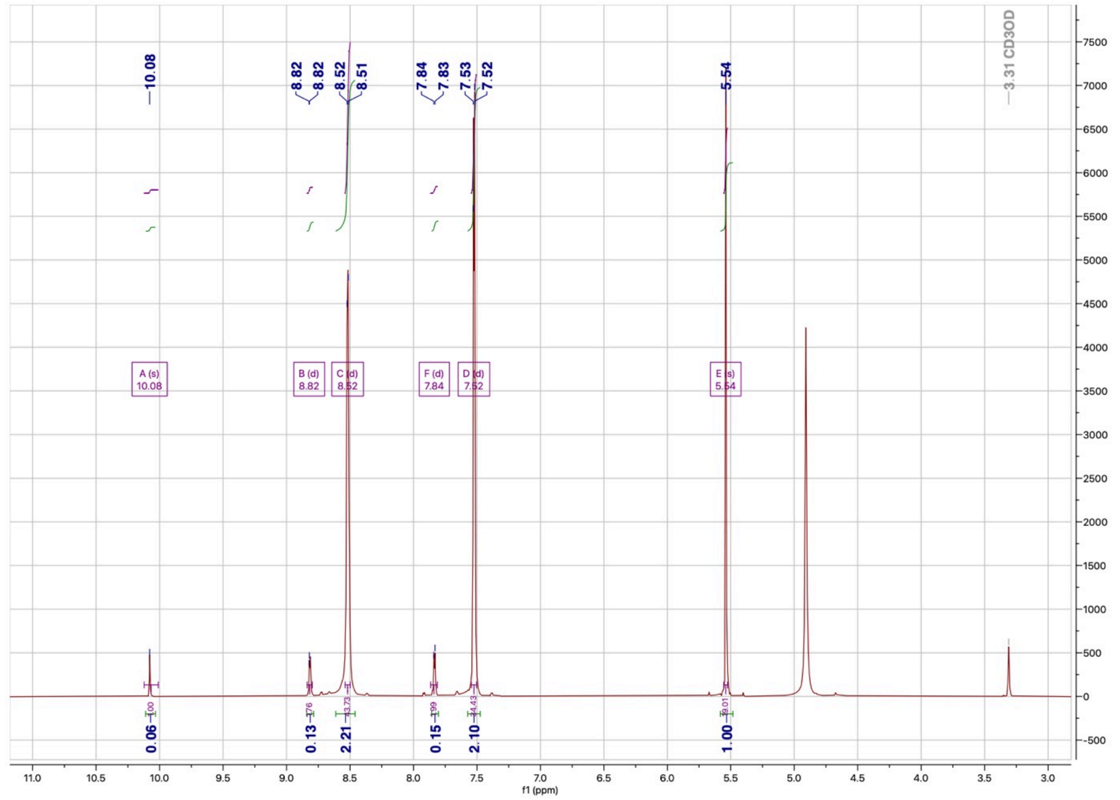

Aldehyde: δ 10.08 (s, -C**H**O), 8.82 (d, *J* = 1.6 Hz, **$H_{2,6}$**), 7.84 (d, *J* = 6.1 Hz, **$H_{3,5}$**); hemiacetal: 8.52 (d, *J* = 5.7 Hz, **$H_{2,6}$**), 7.52 (d, *J* = 6.3 Hz, **$H_{3,5}$**), 5.54 (s, -C**H**(OH)(OCD_3)).

Figure S9. ^{13}C-NMR spectra of **A$_4$** in CD_3OD/NaOH (0.1M): 96% hemiacetal and 4% aldehyde.

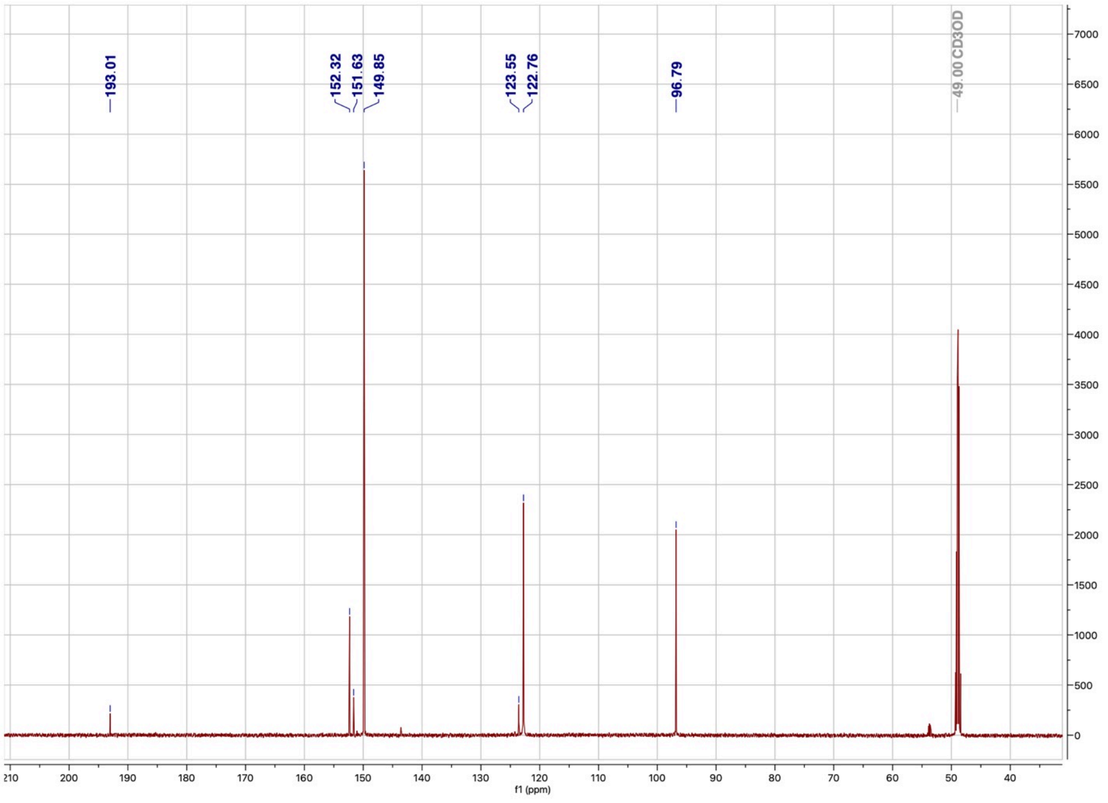

Aldehyde: δ 191.80 (-**C**HO), 151.63 (-**C$_{2,6}$**), 123.55 (-**C$_{3,5}$**); hemiacetal: 152.32 (**C$_4$**), 149.85 (-**C$_{2,6}$**), 122.76 (-**C$_{3,5}$**), 96.79 (-**C**H(OH)(OCD_3)).

Figure S10. ^{1}H-NMR spectra of **A$_4$** in DMSO-*d*$_6$: 100% aldehyde.

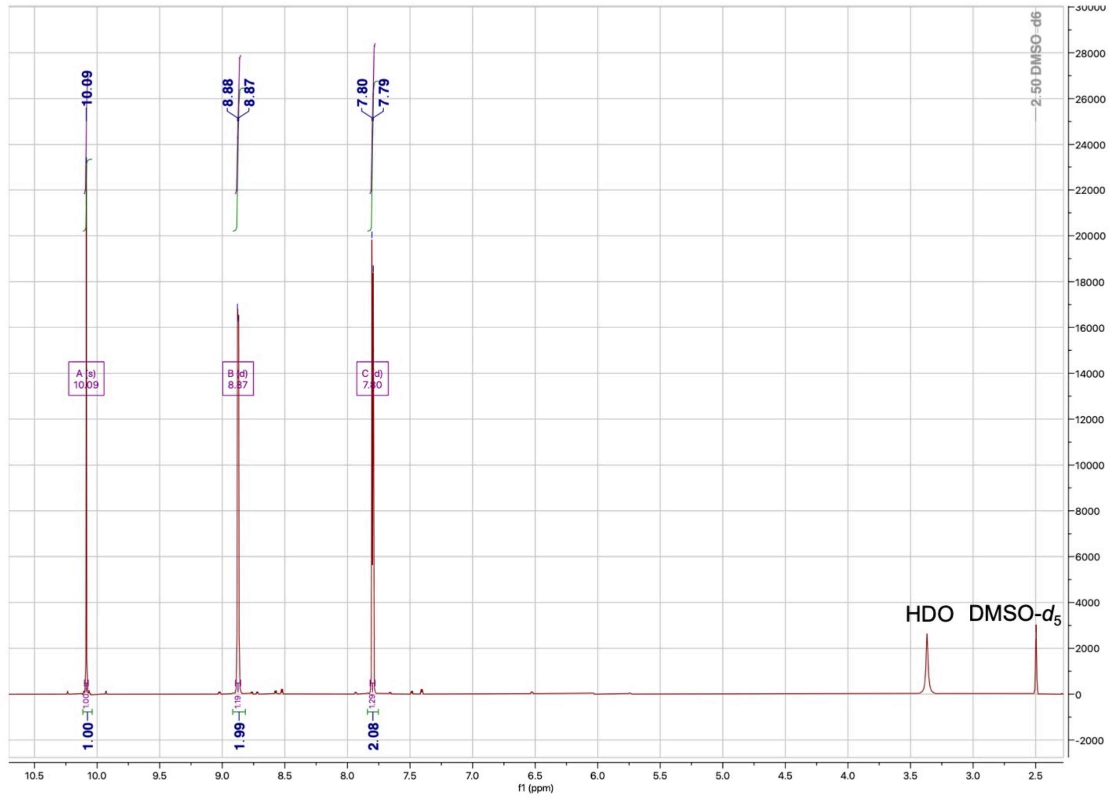

Aldehyde: δ 10.09 (s, -C**H**O), 8.87 (d, *J* = 6.0 Hz, **H$_{2,4}$**), 7.80 (d, *J* = 6.0 Hz, **H$_{3,5}$**). Unassigned peaks correspond to satellite signals and impurities that represent less than 1% in the sample.

Figure S11. ^{1}H-NMR spectra of **A_4** in DMSO-d_6/TFA: 99% aldehyde and 1% *gem*-diol.

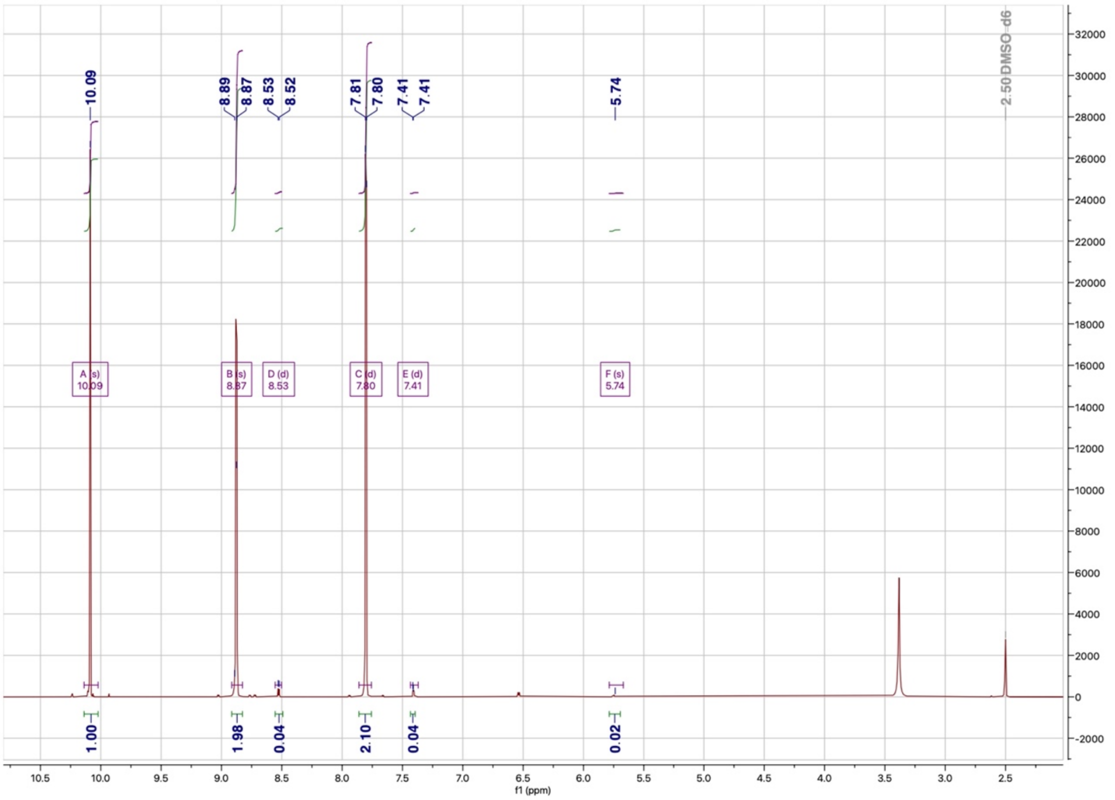

Aldehyde: δ 10.09 (s, -C**H**O), 8.88 (d, J = 4.7 Hz, **$H_{2,6}$**), 7.80 (d, *J* = 5.9 Hz, **$H_{3,5}$**); *gem*-diol: 8.53 (d, *J* = 6.0 Hz, **$H_{2,6}$**), 7.41 (d, *J* = 2.3 Hz, **$H_{3,5}$**), 5.74 (s, -C**H**$(OH)_2$). The resonance signal at 6.58 ppm belongs to the OH group.

Figure S12. ^{1}H-NMR spectra of **A_4** in DMSO-d_6/NaOH (0.4M): 82% aldehyde and 18% *gem*-diol.

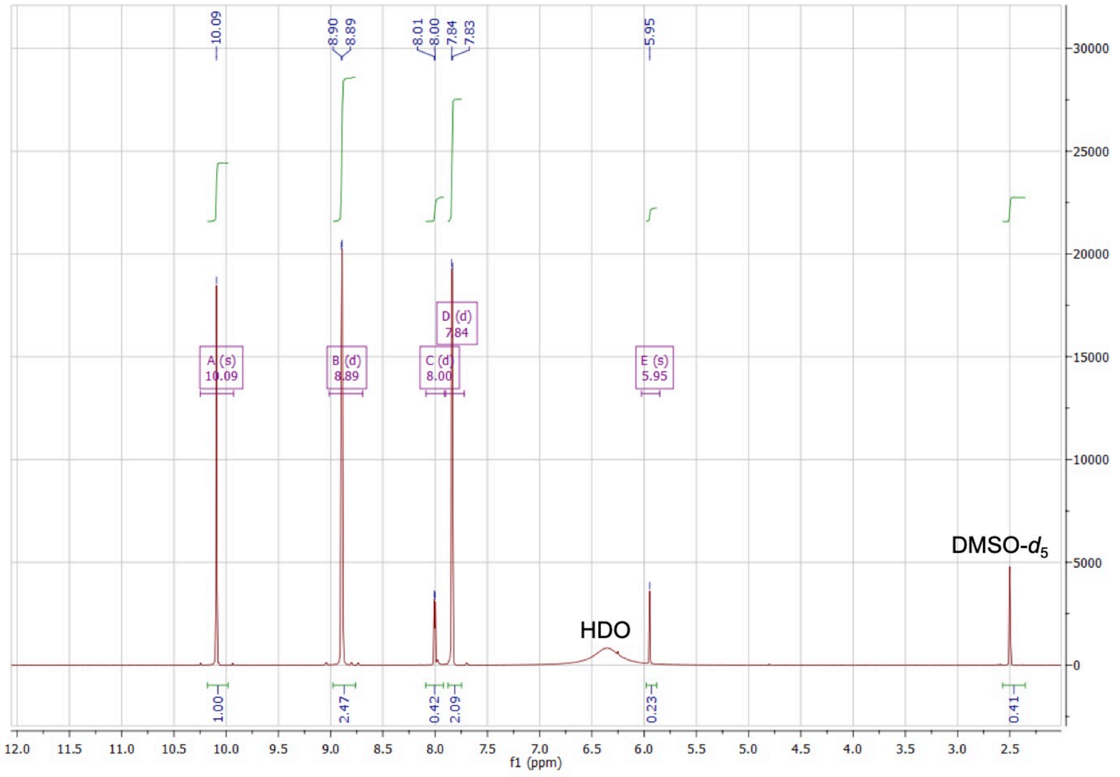

Aldehyde: δ 10.09 (s, -C**H**O), 7.84 (d, J = 4.9 Hz, **$H_{3,5}$**); *gem*-diol: 8.89 (d, J = 4.6 Hz, $H_{2,6}$), 8.00 (d, J = 5.7 Hz, **$H_{3,5}$**), 5.95 (s, -C**H**$(OH)_2$).

Figure S13. ^{1}H-NMR spectra of solid crystals of **A**$_4$ dissolved in DMSO-*d*$_6$: 47% aldehyde, 48% *gem*-diol, 5% and hemiacetal.

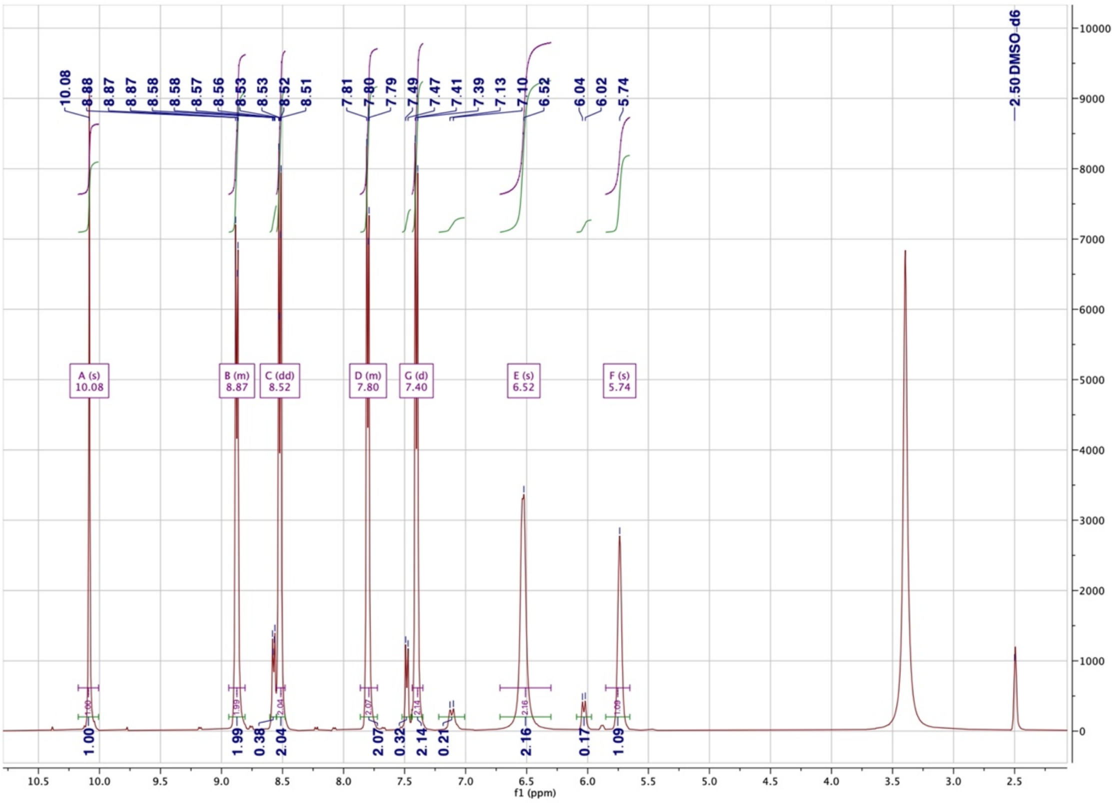

Aldehyde: δ 10.08 (s, -C**H**O), 8.87-8.58 (m, **H**$_{2,6}$), *gem*-diol: 8.52 (dd, J = 4.5, 1.4 Hz, **H**$_{2,6}$), 7.86 – 7.72 (m, H$_{3,5}$), 7.40 (d, J = 5.9 Hz, 2H), 6.52 (s, -C**H**(OH)$_2$); Hemiacetal: 8.87-8.58 (m, **H**$_{2,6}$), 7.86 – 7.72 (m, **H**$_{3,5}$), 5.74 (s, -C**H**(OH)(OR)).

Figure S14. ^{1}H-NMR spectra of solid crystals of **A_4** dissolved in DMSO-d_6 and a drop of D_2O: 88% aldehyde and 22% *gem*-diol.

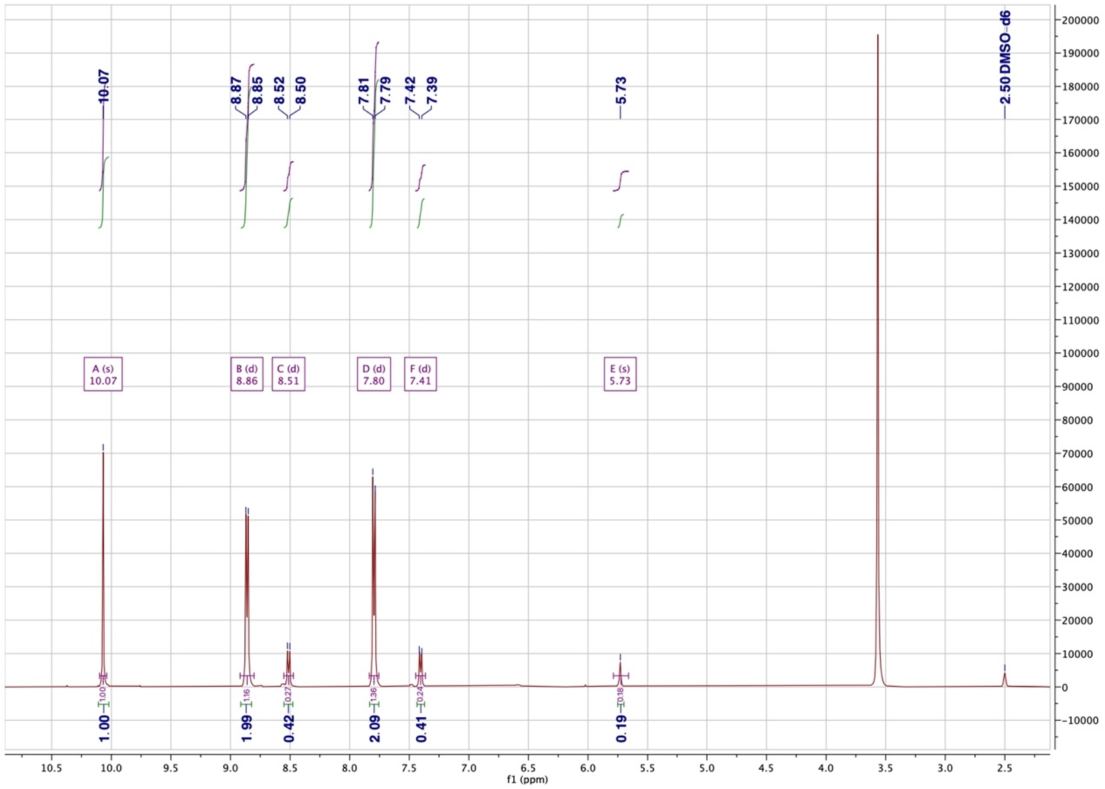

Aldehyde: δ 10.07 (s, -C**H**O), 8.86 (d, J = 6.0 Hz, **$H_{2,6}$**), 7.80 (d, J = 6.0 Hz, **$H_{3,5}$**). *gem*-diol: 8.51 (d, J = 6.0 Hz, **$H_{2,6}$**), 7.41 (d, J = 6.6 Hz, **$H_{3,5}$**), 5.73 (s, -C**H**$(OH)_2$).

Figure S15. ^{1}H-NMR spectra of $\mathbf{A_2}$ in D_2O: 61% aldehyde, 30% *gem*-diol and 9% carboxylic acid.

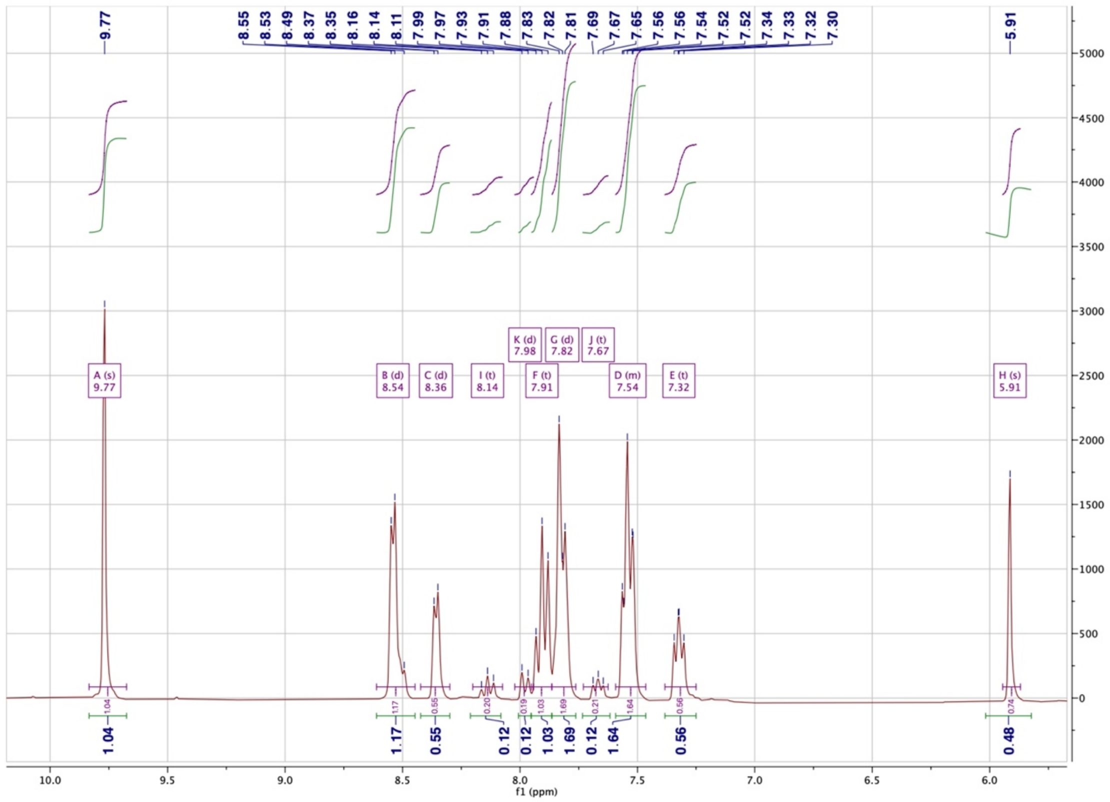

Aldehyde: δ 9.77 (s, -C**H**O), 8.54 (d, *J* = 4.7 Hz, $\mathbf{H_6}$), 7.91 (t, *J* = 7.6 Hz, $\mathbf{H_4}$), 7.85-7.79 (m, $\mathbf{H_3}$), 7.60-7.47 (m, $\mathbf{H_5}$). *Gem*-diol: δ 8.36 (d, *J* = 4.9 Hz, $\mathbf{H_6}$), 7.85-7.79 (m, $\mathbf{H_4}$), 7.60-7.47 (m, $\mathbf{H_3}$), 7.32 (dd, *J* = 6.7, 5.8 Hz, $\mathbf{H_5}$), 5.91 (s, -C**H**$(OH)_2$). Carboxylic acid: δ 8.55-8.49 (m, $\mathbf{H_6}$), 8.14 (t, *J* = 7.7 Hz, $\mathbf{H_4}$), 7.98 (d, *J* = 7.8 Hz, $\mathbf{H_3}$), 7.69-7.63 (m, $\mathbf{H_5}$).

Figure S16. ^{1}H-NMR spectra of $\mathbf{A_2}$ in D_2O/TFA: 100% *gem*-diol.

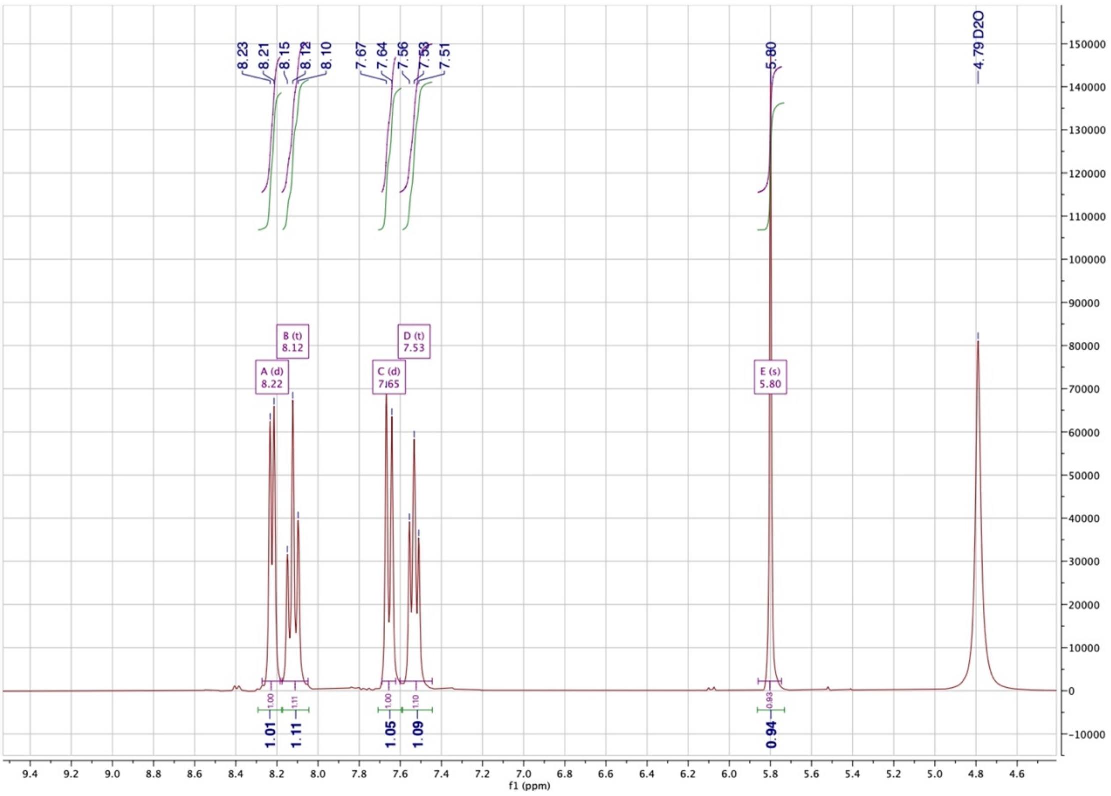

gem-diol: δ 8.21 (d, J = 5.7 Hz, $\mathbf{H_6}$), 8.11 (t, J = 7.9 Hz, $\mathbf{H_4}$), 7.64 (d, J = 8.1 Hz, $\mathbf{H_3}$), 7.52 (t, J = 6.8 Hz, $\mathbf{H_5}$), 5.80 (s, 1H, -C**H**$(OH)_2$). Unassigned peaks correspond to satellite signals and impurities that represent less than 1% in the sample.

Figure S17. ^{1}H-NMR spectra of **A$_2$** in D_2O/NaOH (0.1M): 48% Aldehyde, 21% *gem*-diol, 21% carboxylic acid and 10% Hydroxymethyl. Considering the complexity of the NMR spectra, only the signals corresponding to the aldehyde, *gem*-diol, sodium carboxylate and hydroxymethyl moieties were used. The unequivocal assignment of the NMR signals has been done from the 2D-NMR spectra.

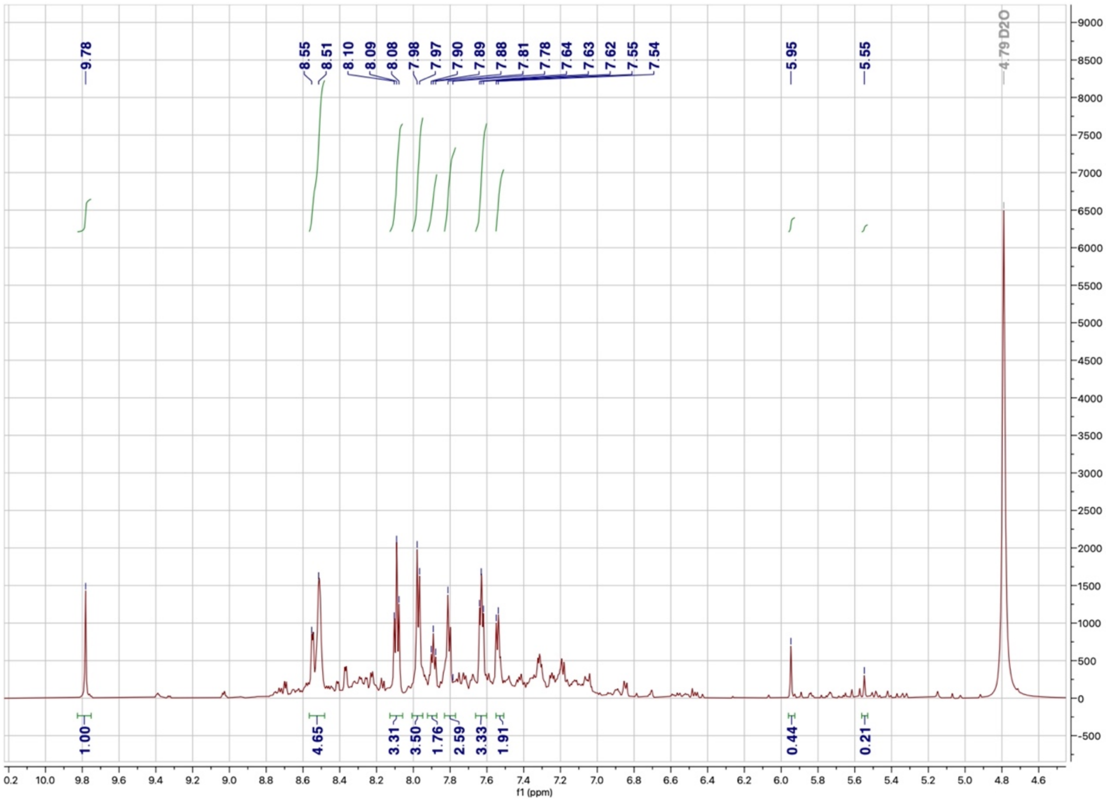

Aldehyde: δ 9.78 (s, -C**H**O), 8.54 (d, *J* = 4.7 Hz, **H$_6$**), 7.91 (t, *J* = 7.6 Hz, **H$_4$**), 7.85-7.79 (m, **H$_3$**), 7.60-7.47 (m, **H$_5$**). *Gem*-diol: δ 8.09 (d, *J* = 4.9 Hz, **H$_6$**), 7.85-7.79 (m, **H$_4$**), 7.60-7.47 (m, **H$_3$**), 5.95 (s, -C**H**(OH)$_2$). Carboxylic acid: 7.98 (d, *J* = 7.8 Hz, **H$_3$**); Hydroxymethyl: 5.65 (s, -C**H$_2$**OH).

Figure S17a. ^{13}C-NMR spectra of $\mathbf{A_2}$ in D_2O/NaOH (0.1M): 48% aldehyde, 21% *gem*-diol, 21% Carboxylic acid, 10% Hydroxymethyl. Considering the complexity of the NMR spectra, only the signals corresponding to the aldehyde, *gem*-diol, sodium carboxylate and hydroxymethyl moieties were used. The unequivocal assignment of the NMR signals has been done from the 2D-NMR spectra.

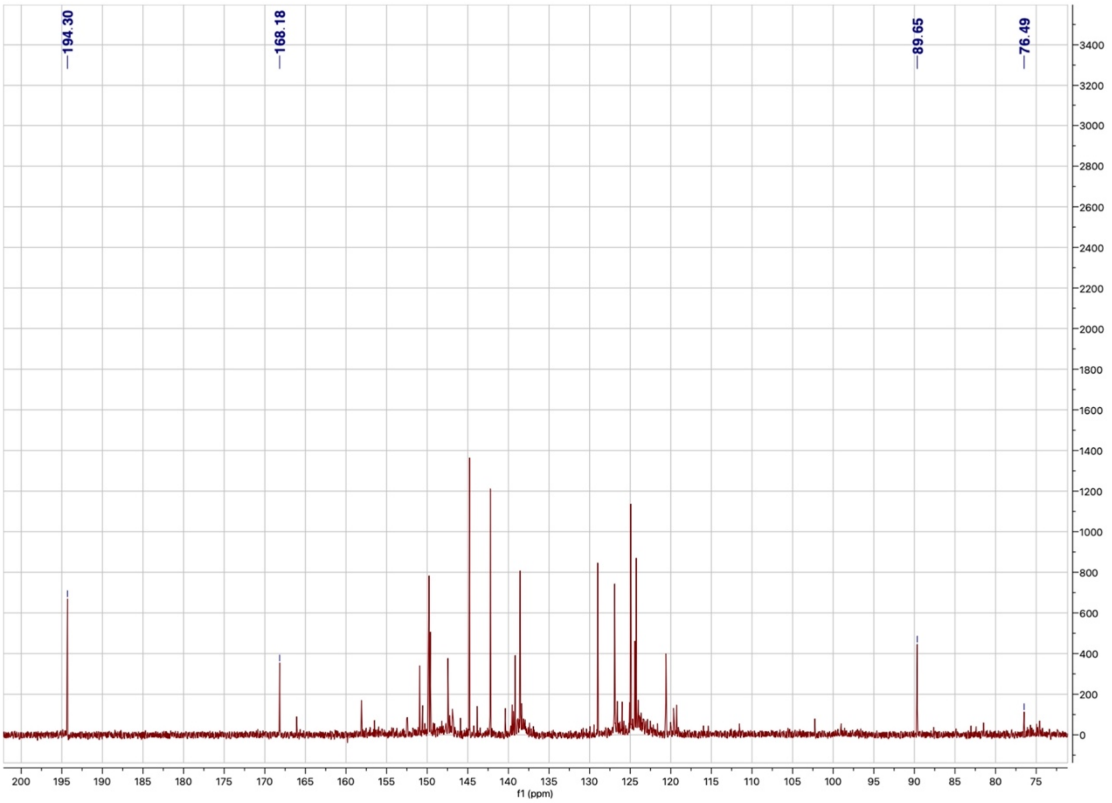

Aldehyde: δ 194.30 (-**C**HO); Carboxylic acid: 168.18 (-**C**OOH); *gem*-diol: 89.65 (-**C**H(OH)$_2$); Hydroxymethyl: 76.49 (-**C**H$_2$OH).

Figure S18. ^{1}H-NMR spectra of **A_2** in CD_3OD: 68% hemiacetal, 19% aldehyde and 13% carboxylic acid.

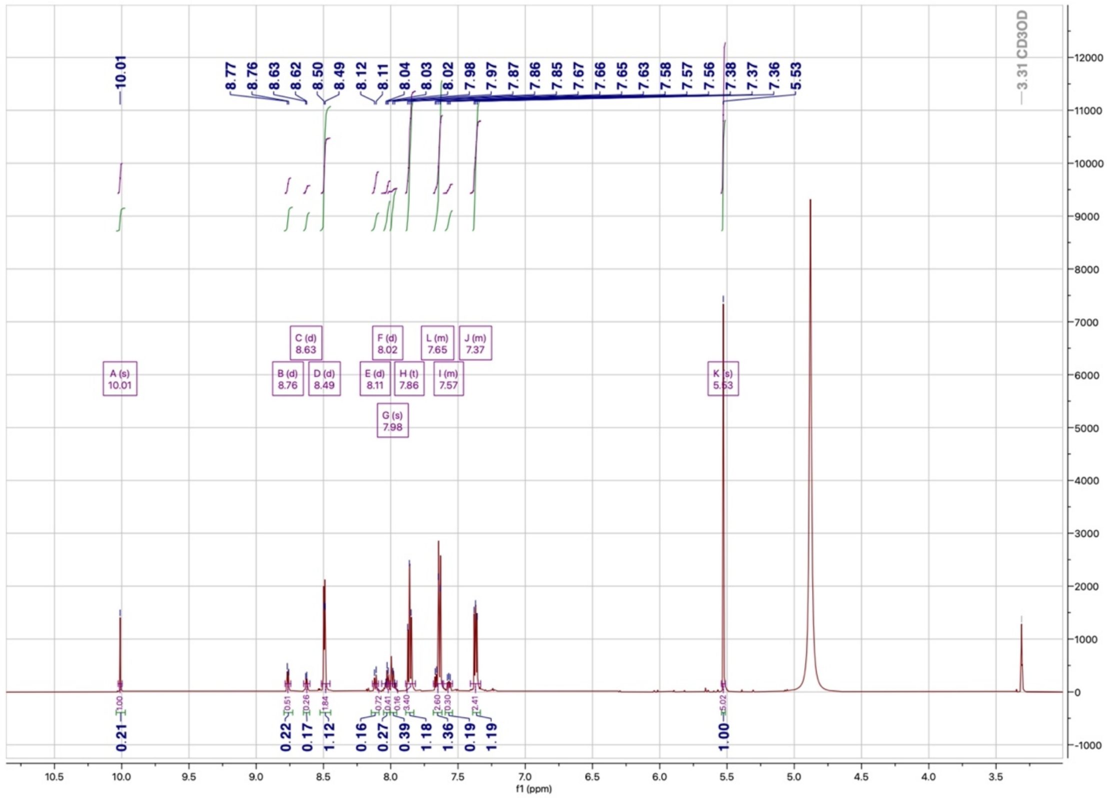

Aldehyde: δ 10.01 (s, -C**H**O), 8.76 (d, *J* = 6.3 Hz, **H_6**), 8.02 (d, *J* = 5.7 Hz, **H_4**), 7.68 – 7.62 (m, **H_5**); Hemiacetal: 8.49 (d, *J* = 4.9 Hz, **H_6**), 7.86 (t, *J* = 7.7 Hz, **H_5**), 7.41 – 7.33 (m, **H_3**), 5.53 (s, -C**H**(OH)(OCD_3)). Carboxylic acid: 8.63 (d, *J* = 4.8 Hz, **H_6**), 8.11 (d, *J* = 7.8 Hz, **H_4**), 7.98 (s, **H_3**), 7.61 – 7.54 (m, **H_5**).

Figure S19. ^{13}C-NMR spectra of **A$_2$** in CD_3OD: 68% hemiacetal, 19% aldehyde and 13% carboxylic acid.

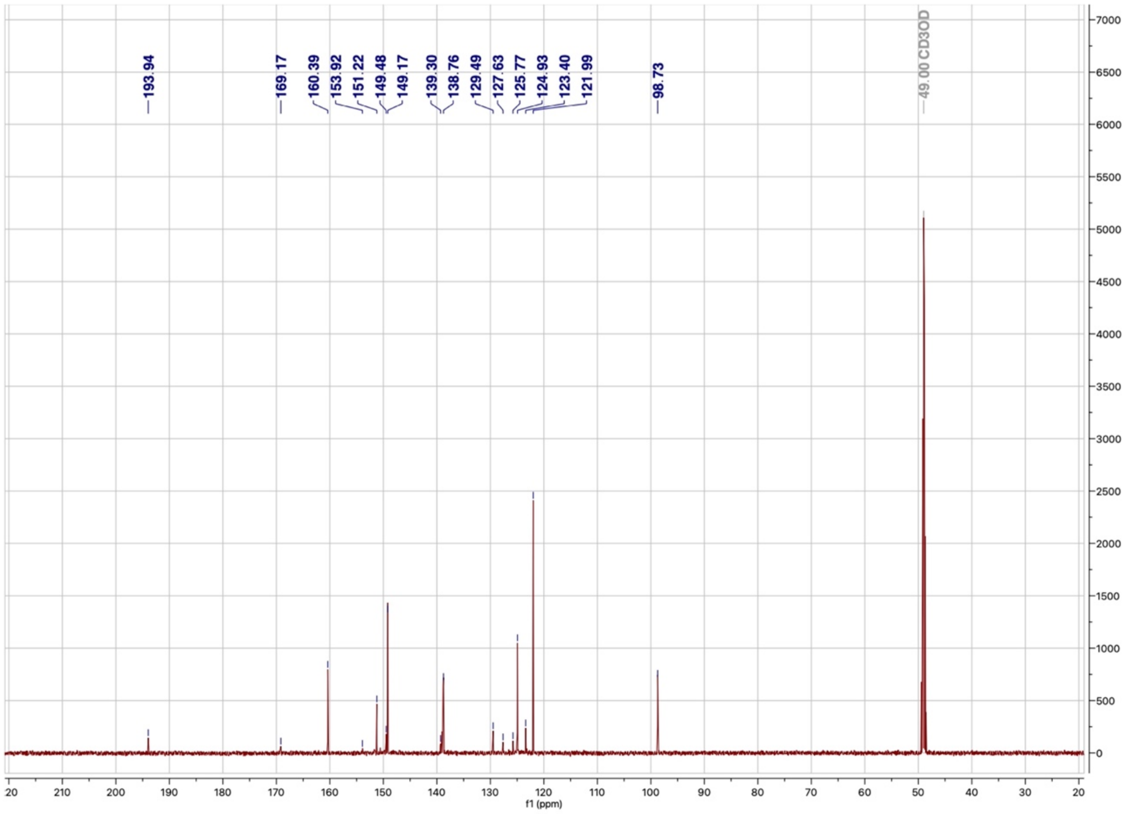

Aldehyde: δ 193.94 (-**C**HO); hemiacetal: 98.73 (-**C**H(OH)(OCD_3); Carboxylic acid: 169.17 (-**C**OOH).

Figure S20. ^{1}H-NMR spectra of **A_2** in CD_3OD/NaOH (0.1M): 68% hemiacetal, 19% aldehyde and 13% sodium carboxylate.

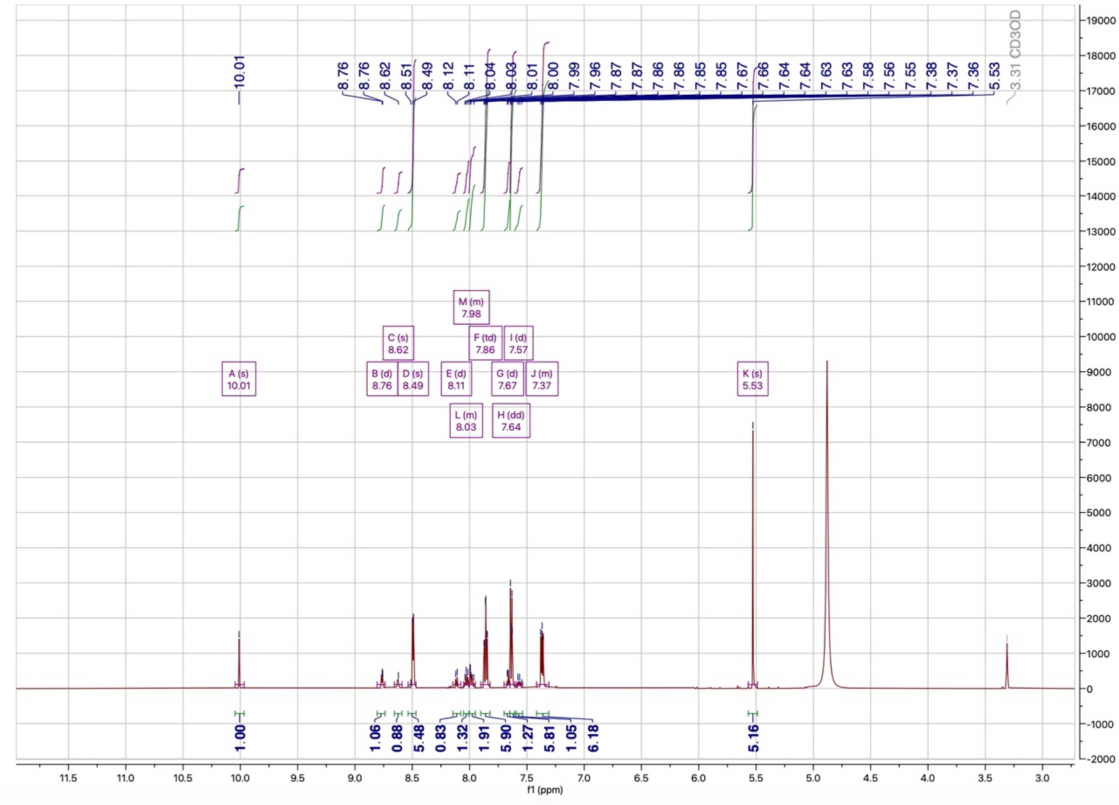

Aldehyde: δ 10.01 (s, -C**H**O), 8.76 (d, *J* = 6.3 Hz, **H_6**), 8.02 (d, *J* = 5.7 Hz, **H_4**), 7.68 – 7.62 (m, **H_5**); Hemiacetal: 8.49 (d, *J* = 4.9 Hz, **H_6**), 7.86 (t, *J* = 7.7 Hz, **H_5**), 7.41 – 7.33 (m, **H_3**), 5.53 (s, -C**H**(OH)(OCD_3)). Carboxylic acid: 8.62 (d, *J* = 4.8 Hz, **H_6**), 8.11 (d, *J* = 7.8 Hz, **H_4**), 7.98 (s, **H_3**), 7.61 – 7.54 (m, **H_5**).

Figure S21. ^{13}C-NMR spectra of **A_2** in CD_3OD/NaOH (0.1M): 68% hemiacetal, 19% aldehyde and 13% sodium carboxylate.

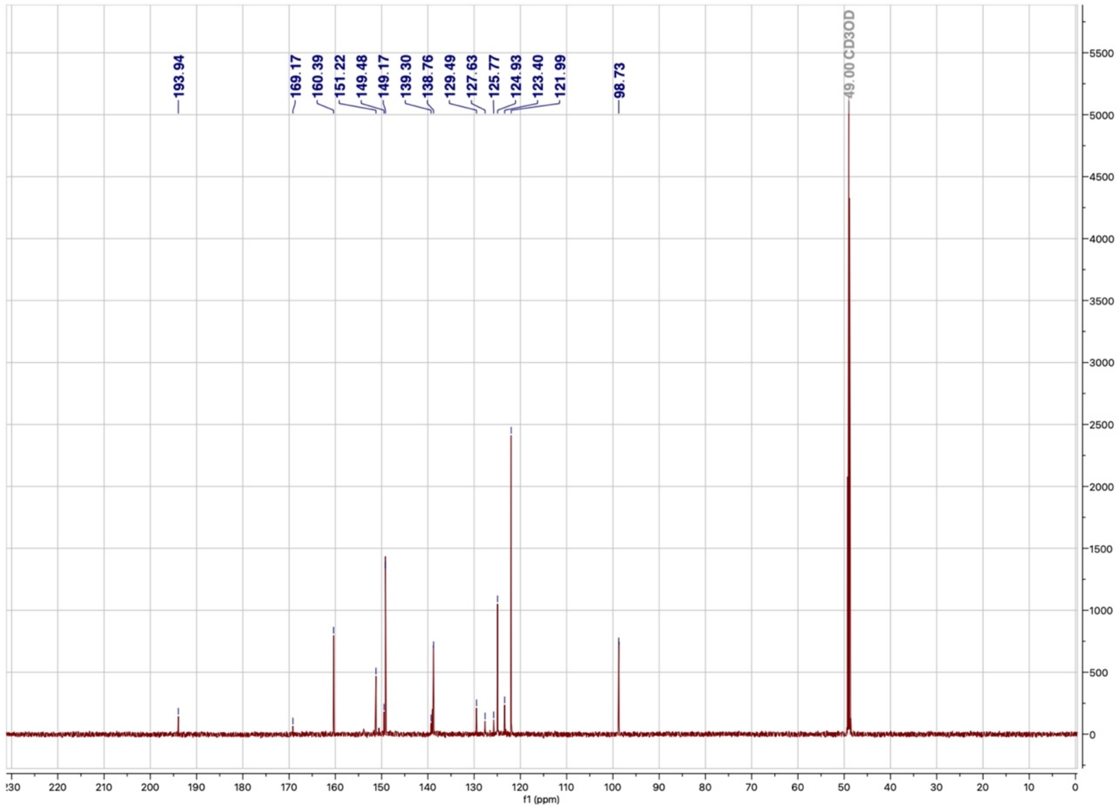

Aldehyde: δ 193.94 (-CHO), 151.22 (C_2), 129.49 (C_3), 123.40 (C_5),; hemiacetal: 160.39 (C_6), 149.48 (C_4), 138.76 (C_2), 124.93 (C_3), 121.99 (C_5), 98.73 (-**C**H(OH)(OCD_3); sodium carboxylate: 169.17 (-COONa), 139.30 (C_4), 127.63 (C_3), 125.77 (C_5),

Figure S22. ^{1}H-NMR spectra of **A₂** in CD_3OD/TFA: 13% Carboxylic acid, 85% hemiacetal and 2% aldehyde.

Considering the complexity of the NMR spectra, only the signals corresponding to the aldehyde, hemiacetal and acetal were used. The unequivocal assignment of the NMR signals has been done from the 2D-NMR spectra. The amount of carboxylic acid was estimated from the ^{1}H-NMR spectrum of **A₂** in CD_3OD.

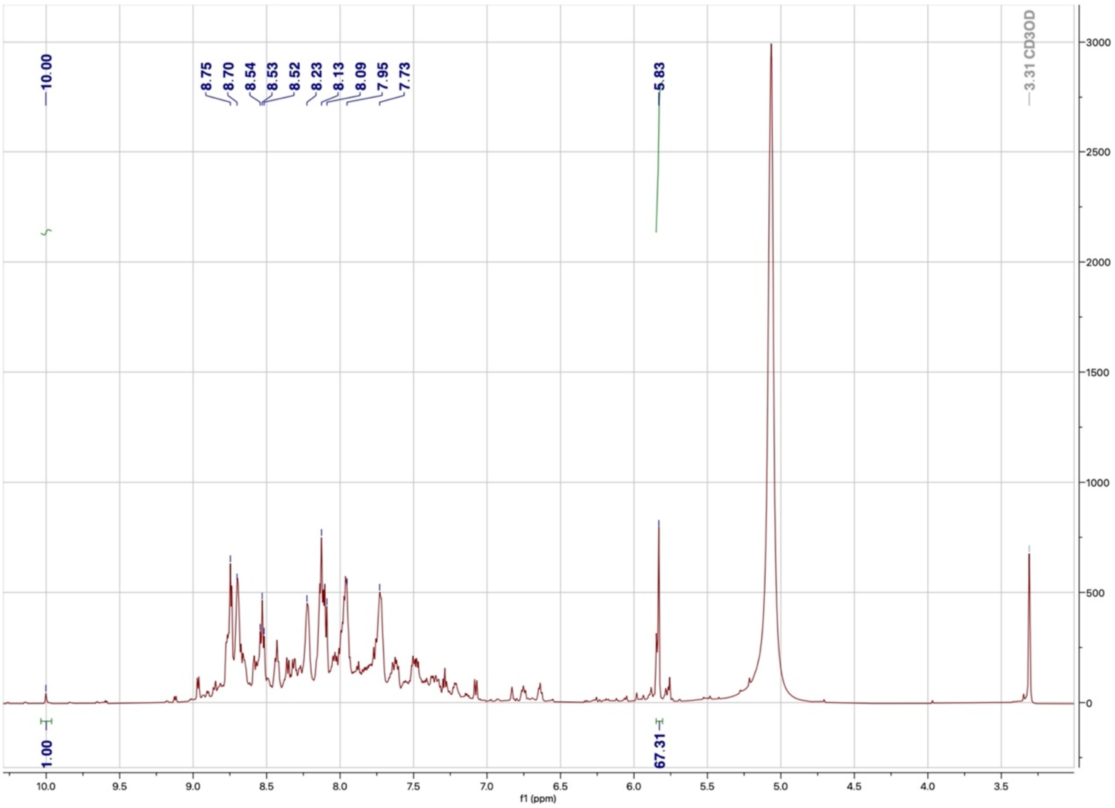

Figure S23. ^{1}H-NMR spectra of $\mathbf{A_2}$ in DMSO-d_6, DMSO-d_6/NaOH and DMSO-d_6/TFA:

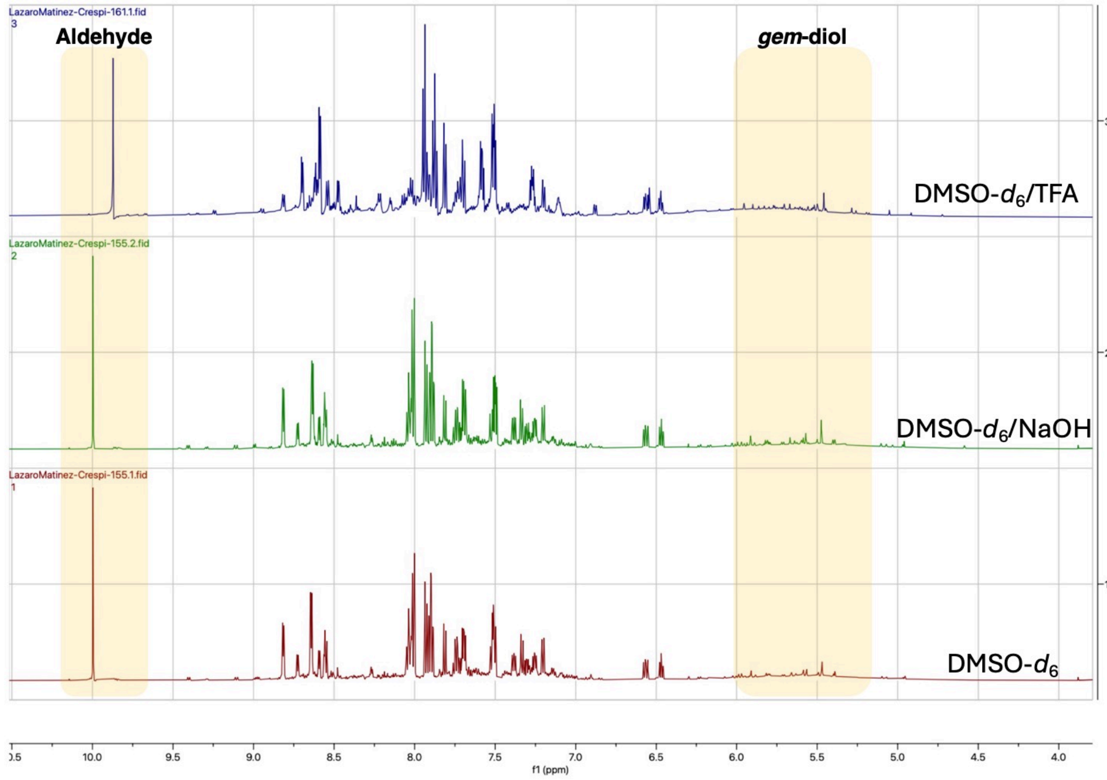

Figure S24. ^{1}H-NMR spectra of $\mathbf{A_3}$ in D_2O: 90% aldehyde and 10% *gem*-diol.

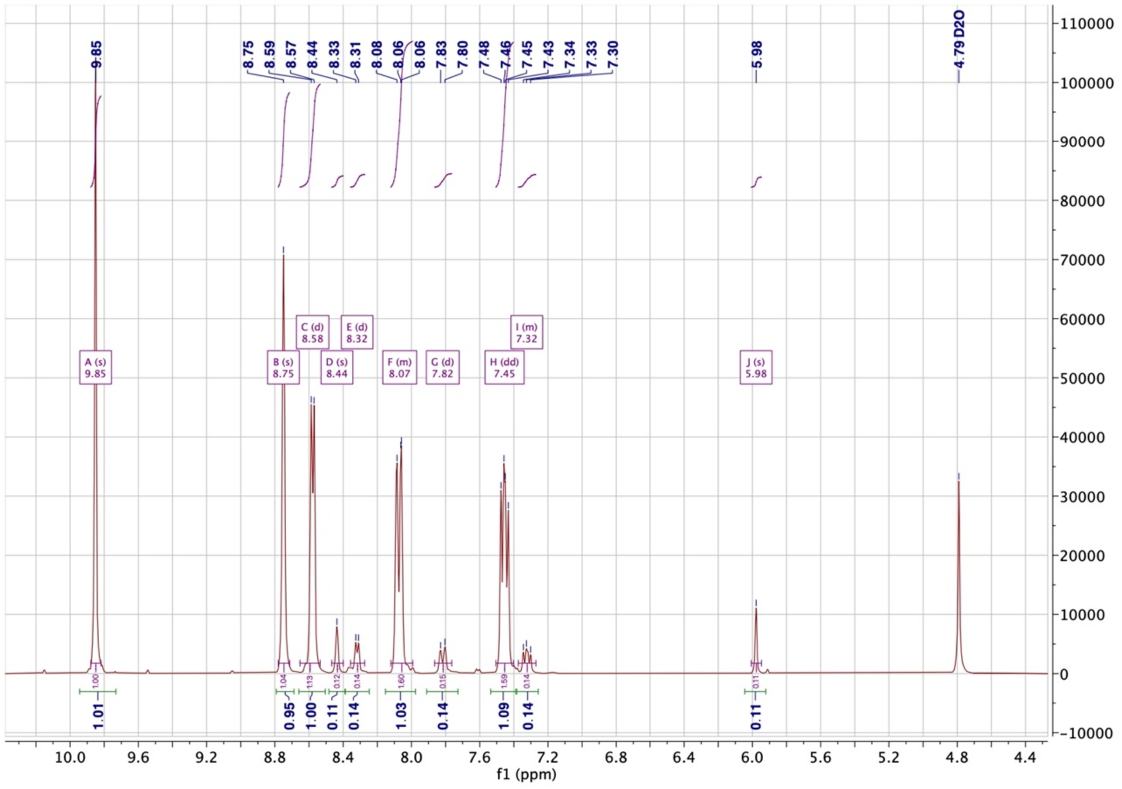

Aldehyde: δ 9.85 (s, -C**H**O), 8.75 (s, $\mathbf{H_2}$), 8.58 (d, J = 4.9 Hz, $\mathbf{H_6}$), 8.15-8.02 (m, $\mathbf{H_4}$), 7.45 (dd, J = 7.5, 5.3 Hz, $\mathbf{H_5}$); *gem*-diol: δ 8.44 (s, $\mathbf{H_2}$), 8.32 (d, J = 4.9 Hz, $\mathbf{H_6}$), 7.82 (d, J = 7.4 Hz, $\mathbf{H_4}$), 7.37-7.27 (m, $\mathbf{H_5}$), 5.98 (s, -C**H**$(OH)_2$).

Figure S25. ^{1}H-NMR spectra of **A_3** in D_2O/TFA: 15% aldehyde and 85% *gem*-diol.

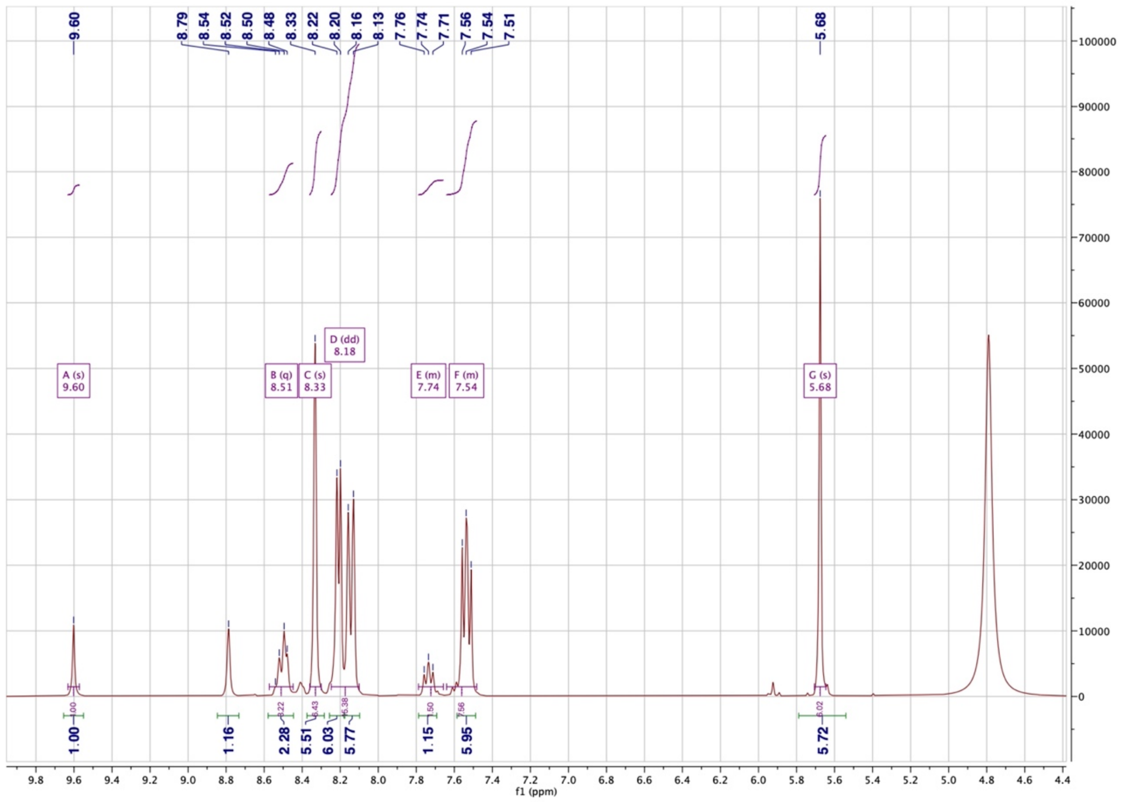

Aldehyde: δ 9.60 (s, -C**H**O), 8.79 (s, **H_2**), 8.56-8.46 (m, **$H_{4\ \&\ 6}$**), 7.74 (t, *J* = 6.9 Hz, **H_5**); *gem*-diol: δ 8.33 (s, **H_2**), 8.21 (d, *J* = 5.6 Hz, **H_6**), 8.14 (d, *J* = 8.1 Hz, **H_4**), 7.59-7.49 (m, **H_5**), 5.68 (s, -C**H**$(OH)_2$). Unassigned peaks correspond to satellite signals and impurities that represent less than 1% in the sample.

Figure S26. ^{1}H-NMR spectra of **A$_3$** in D_2O/NaOH (0.1M): 12% aldehyde, 70% *gem*-diol, 9% Hydroxymethyl and 9% carboxylic acid.

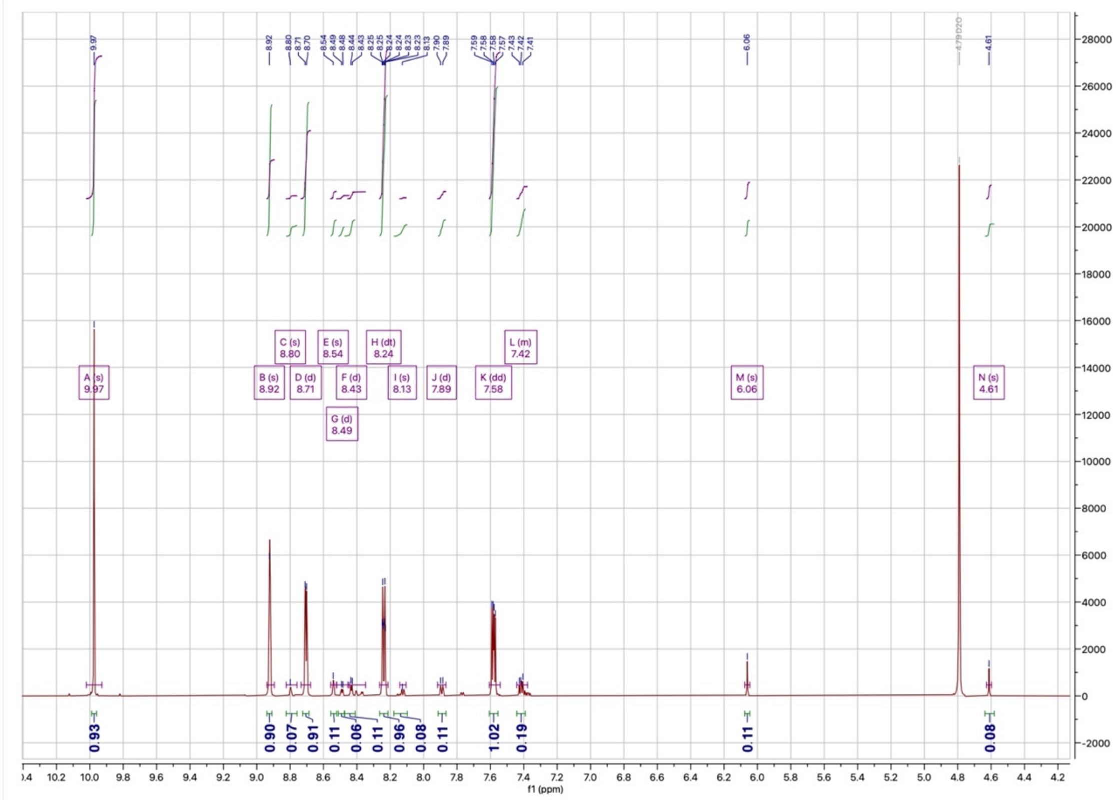

Aldehyde: δ 9.97 (s, -C**H**O), 8.92 (s, **H$_{2al}$**), 8.71 (d, J = 4.9 Hz, **H$_6$**), 8.24 (dt, J = 8.0, 2.0 Hz, **H$_4$**), 7.58 (dd, J = 8.1, 4.8 Hz, **H$_5$**); *gem*-diol: 8.54 (s, **H$_2$**), 7.44 – 7.38 (m, **H$_5$**), 6.06 (s, -C**H**(OH)$_2$); Hydroxymethyl: 8.43 (d, J = 5.0 Hz, **H$_6$**), 8.13 (s, **H$_2$**), 4.61 (s, -C**H**$_2$OH). carboxylic acid: 8.49 (d, J = 5.0 Hz, **H$_6$**), 8.80 (s, **H$_2$**), 7.89 (d, J = 7.9 Hz, **H$_5$**).

Figure S27. ^{13}C-NMR spectra of **A_3** in D_2O/NaOH (0.1M): 12% aldehyde, 70% *gem*-diol, 9% pyridin-3-ylmethanol and 9% carboxylic acid.

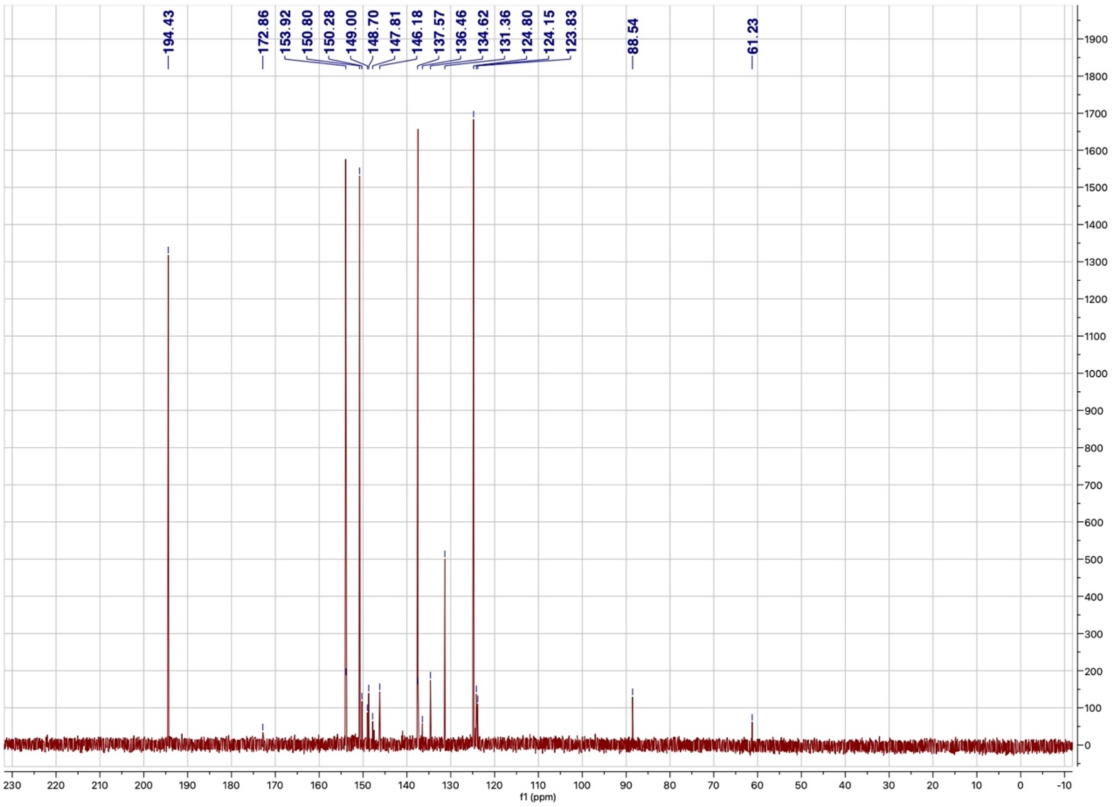

Aldehyde: δ 194.4 (-**C**HO), 153.9 (**C_2**), 150.8 (**C_6**), 137.6 (**C_4**), 131.4 (**C_3**), 124.8 (**C_5**); Carboxylic acid: 172.9 (-**C**OOH); *gem*-diol: 150.3 (**C_2**), 148.7 (**C_6**), 146.2 (**C_4**), 136.5 (**C_3**), 134.6 (**C_5**), 88.5 (-**C**H$(OH)_2$); Hydroxymethyl: 149.0 (**C_2**), 147.8 (**C_4**), 124.2 (**C_3**), 123.8 (**C_5**), 61.2 (-**C**H_2OH).

Figure S28. ^{1}H-NMR spectra of $\mathbf{A_3}$ in CD_3OD: 72% hemiacetal, 27% aldehyde and acetal (<1%).

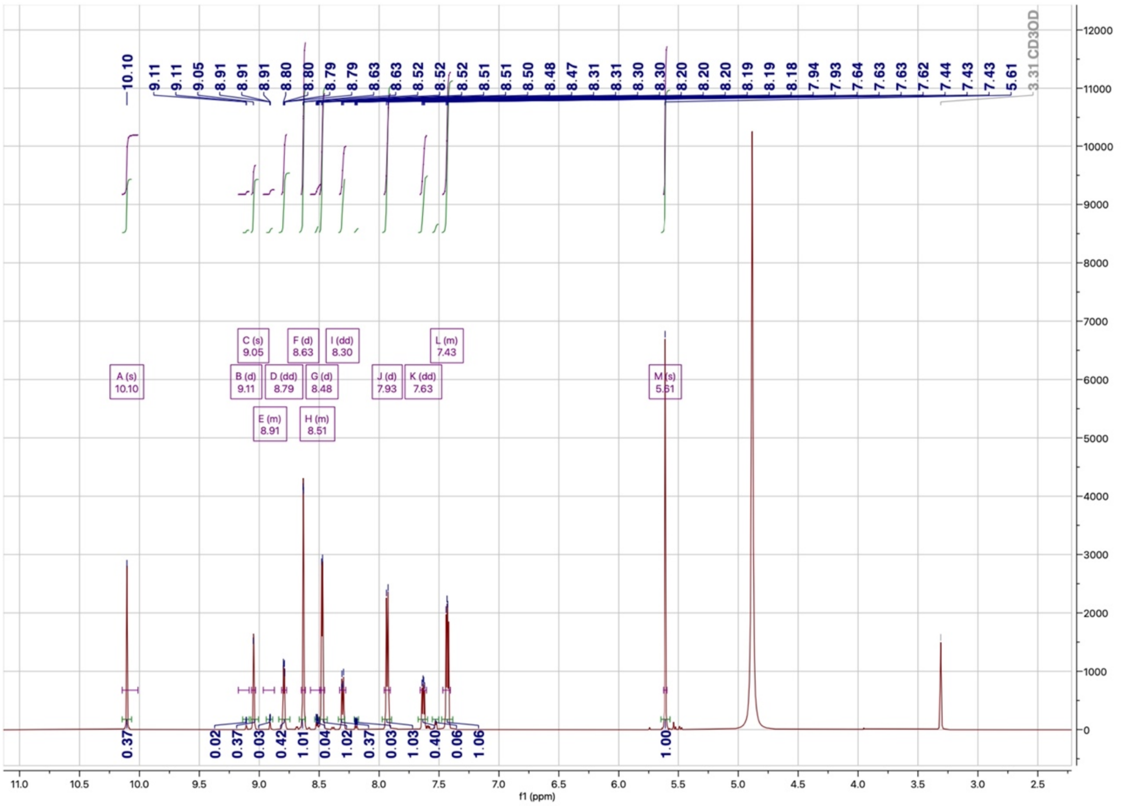

Aldehyde: δ 10.10 (s, -C**H**O), 8.79 (dd, J = 5.0, 1.6 Hz, $\mathbf{H_6}$), 8.57 – 8.48 (m, $\mathbf{H_4}$), 8.48 (d, J = 4.9 Hz, $\mathbf{H_2}$), 7.63 (dd, J = 7.9, 5.0 Hz, $\mathbf{H_5}$); acetal: 9.11 (d, J = 2.1 Hz, $\mathbf{H_2}$), 9.05 (s, $\mathbf{H_6}$), , 7.93 (d, J = 8.0 Hz, H); hemiacetal: 8.97 – 8.87 (m, $\mathbf{H_6}$), 8.63 (d, J = 2.1 Hz, $\mathbf{H_2}$), 8.30 (dd, J = 7.9, 1.7 Hz, $\mathbf{H_4}$), 7.47 – 7.40 (m, $\mathbf{H_5}$), 5.61 (s, -C**H**(OH)(OCD_3)).

Figure S29. ^{13}C-NMR spectra of **A$_3$** in CD_3OD: 72% hemiacetal, 27% aldehyde and 1% acetal.

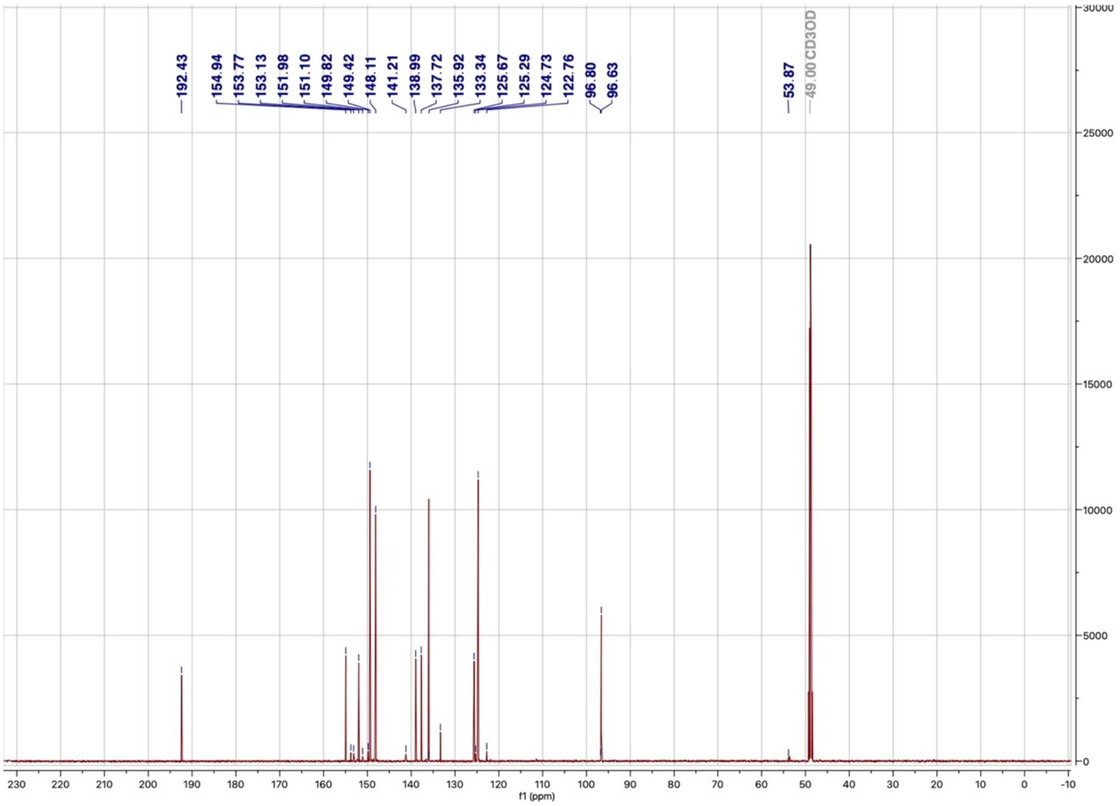

Aldehyde: δ 192.43 (-**C**HO), 154.94 (**C$_2$**), 151.98 (**C$_6$**), 151.10 (**C$_4$**), 133.34 (**C$_3$**), 124.73 (**C$_5$**); hemiacetal: 149.42 (**C$_2$**), 149.82 (**C$_6$**), 149.42 (**C$_4$**), 137.72 (**C$_3$**), 126.67 (**C$_5$**), 96.63 (-**C**H(OH)(OCD_3)), 53.87 (-O**C**D_3); Acetal: 153.77 (**C$_2$**), 153.13 (**C$_4$**), 125.29 (**C$_3$**), 122.76 (**C$_5$**), 96,80 (-**C**H(OCD_3)$_2$).

Figure S30. ^{1}H-NMR spectra of **A_3** in CD_3OD/TFA: 68% hemiacetal, 31% aldehyde, 1% carboxylic acid.

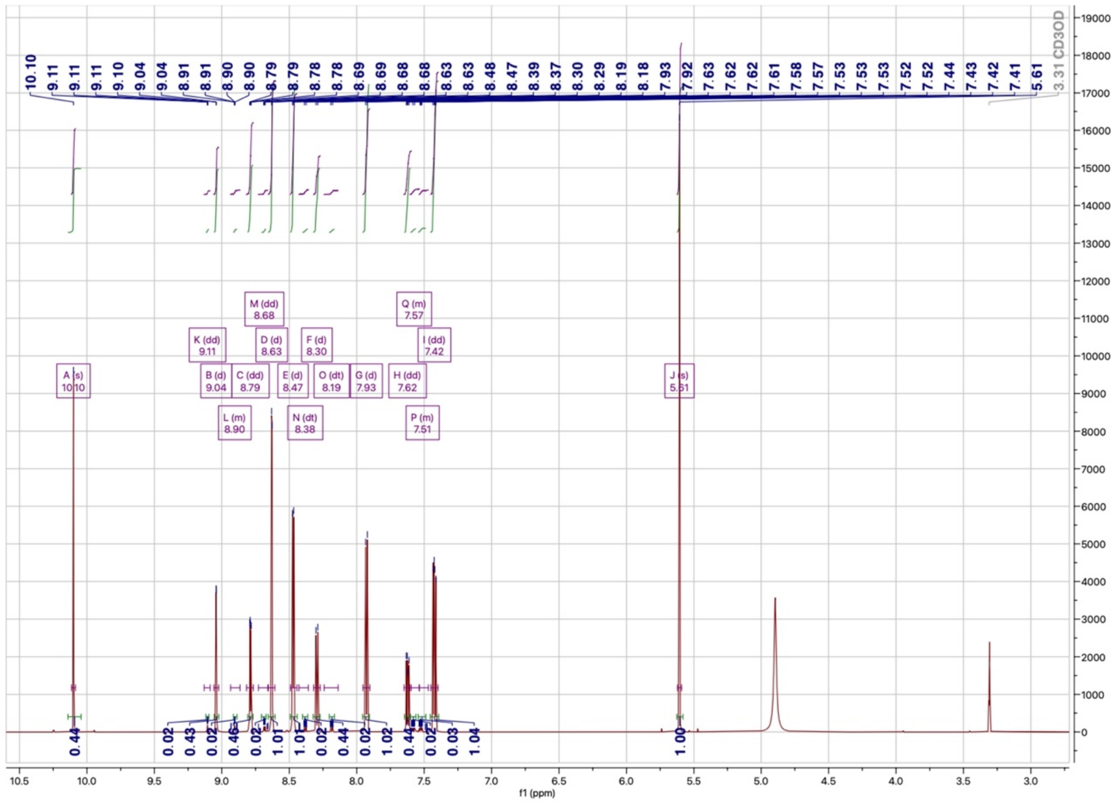

Aldehyde: δ 10.10 (s, -C**H**O), 8.79 (dd, J = 5.0, 1.6 Hz, **H_6**), 8.57 – 8.48 (m, **H_4**), 8.48 (d, J = 4.9 Hz, **H_2**), 7.63 (dd, J = 7.9, 5.0 Hz, **H_5**); Carboxylic acid: 9.11 (d, J = 2.1 Hz, **H_2**), 9.05 (s, **H_6**), 7.93 (d, J = 8.0 Hz, H); hemiacetal: 8.97 – 8.87 (m, **H_6**), 8.63 (d, J = 2.1 Hz, **H_2**), 8.30 (dd, J = 7.9, 1.7 Hz, **H_4**), 7.47 – 7.40 (m, **H_5**), 5.51 (s, -C**H**(OH)(OCD_3)).

Figure S31. ^{1}H-NMR spectra of **A_3** in CD_3OD/NaOH (0.1M): 26% Aldehyde, 74% hemiacetal.

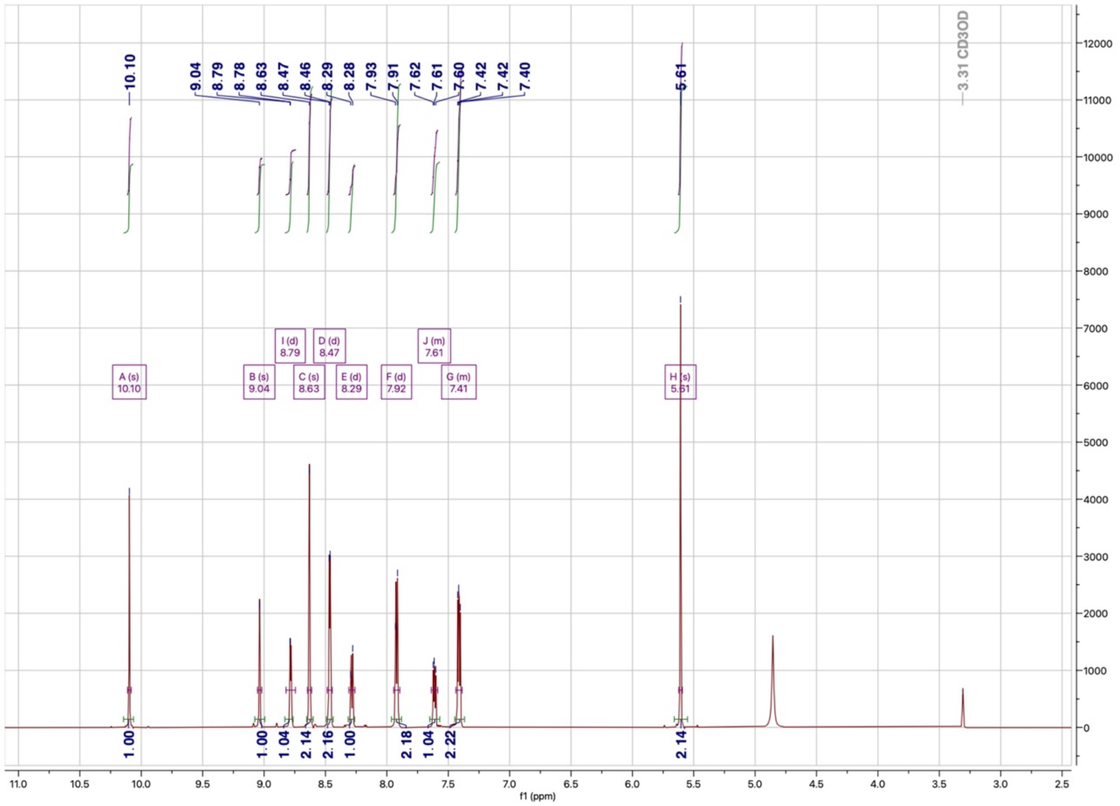

Aldehyde: δ 10.10 (s, -C**H**O), 9.04 (s, **H_2**), 8.79 (d, J = 1.7 Hz, **H_6**), 8.29 (d, J = 9.8 Hz, **H_4**), 7.64 – 7.59 (m, **H_5**); hemiacetal: 8.63 (s, **H_2**), 8.47 (d, J = 4.9 Hz, **H_6**), 7.92 (d, J = 10.0 Hz, **H_4**), 7.44 – 7.39 (m, **H_5**), 5.61 (s, -C**H**(OH)(OCD_3)).

Figure S32. ^{13}C-NMR spectra of **A$_3$** in CD_3OD/NaOH (0.1M): 26% Aldehyde, 74% hemiacetal.

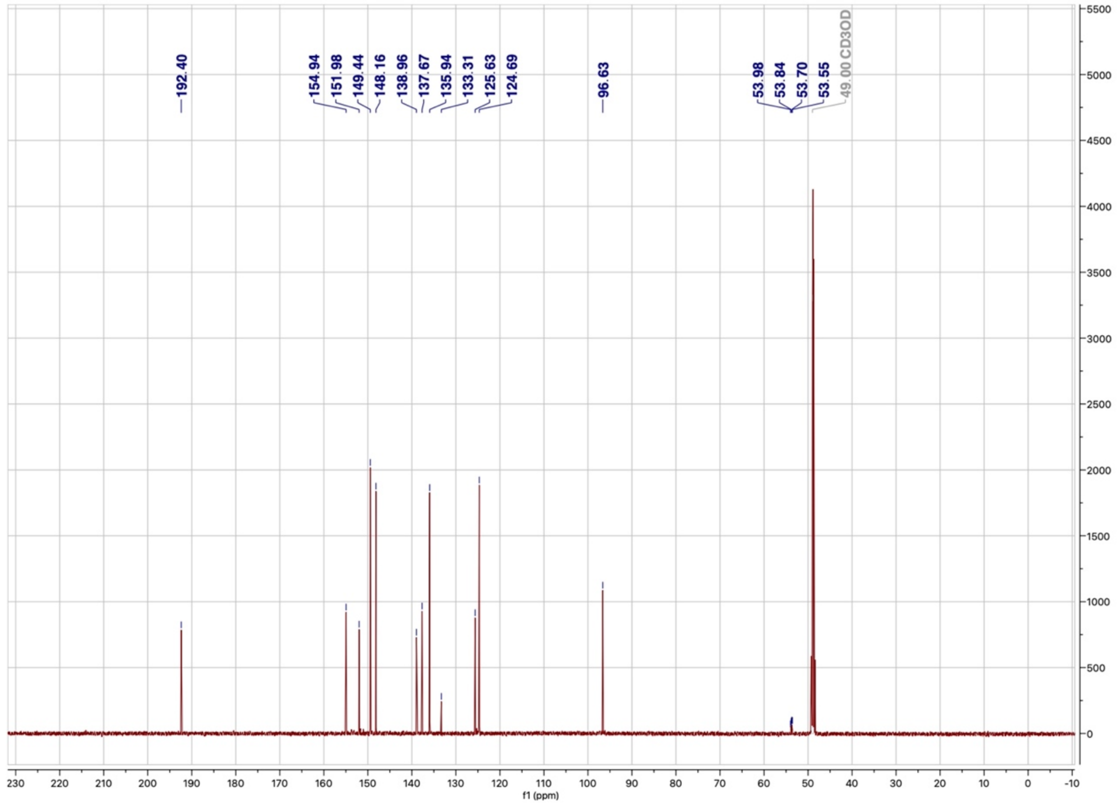

Aldehyde: δ 192.40 (-**C**HO), 154.94 (**C$_2$**), 151.98 (**C$_6$**), 138.96 (**C$_4$**), 133.31 (**C$_3$**), 125.63 (**C$_5$**); hemiacetal: 149.44 (**C$_2$**), 148.16 (**C$_6$**), 137.67 (**C$_3$**), 135.94 (**C$_4$**), 124.69 (**C$_5$**), 96.63 (-**C**H(OH)(OCD_3)), 53.77 (-CH(OH)(O**C**D_3)).

Figure S33. ^{1}H-NMR spectra of **A$_3$** in DMSO-d_6: 100% aldehyde.

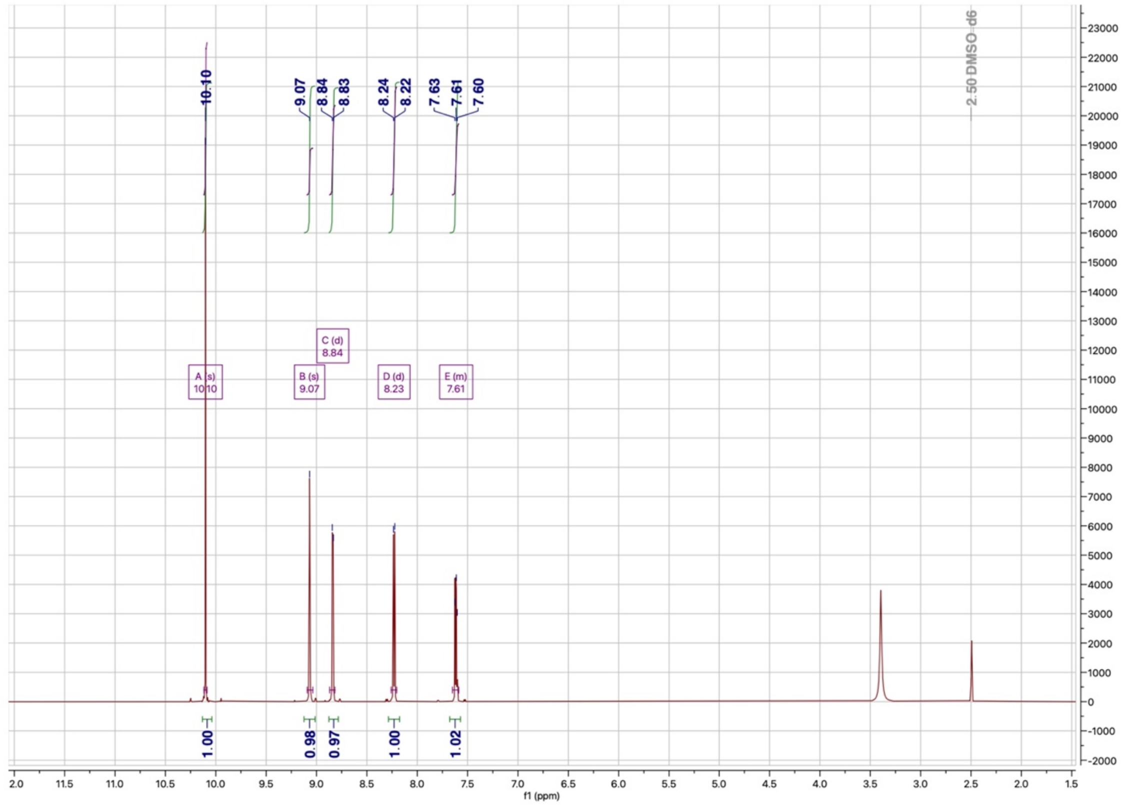

Aldehyde: δ 10.10 (s, -C**H**O), 9.07 (s, **H$_2$**), 8.84 (d, J = 6.7 Hz, **H$_6$**), 8.23 (d, J = 7.9 Hz, **H$_4$**), 7.65 – 7.59 (m, **H$_5$**).

Figure S34. ^{1}H-NMR spectra of **A$_3$** in DMSO-d_6/TFA: 97% Aldehyde, 3% *gem*-diol.

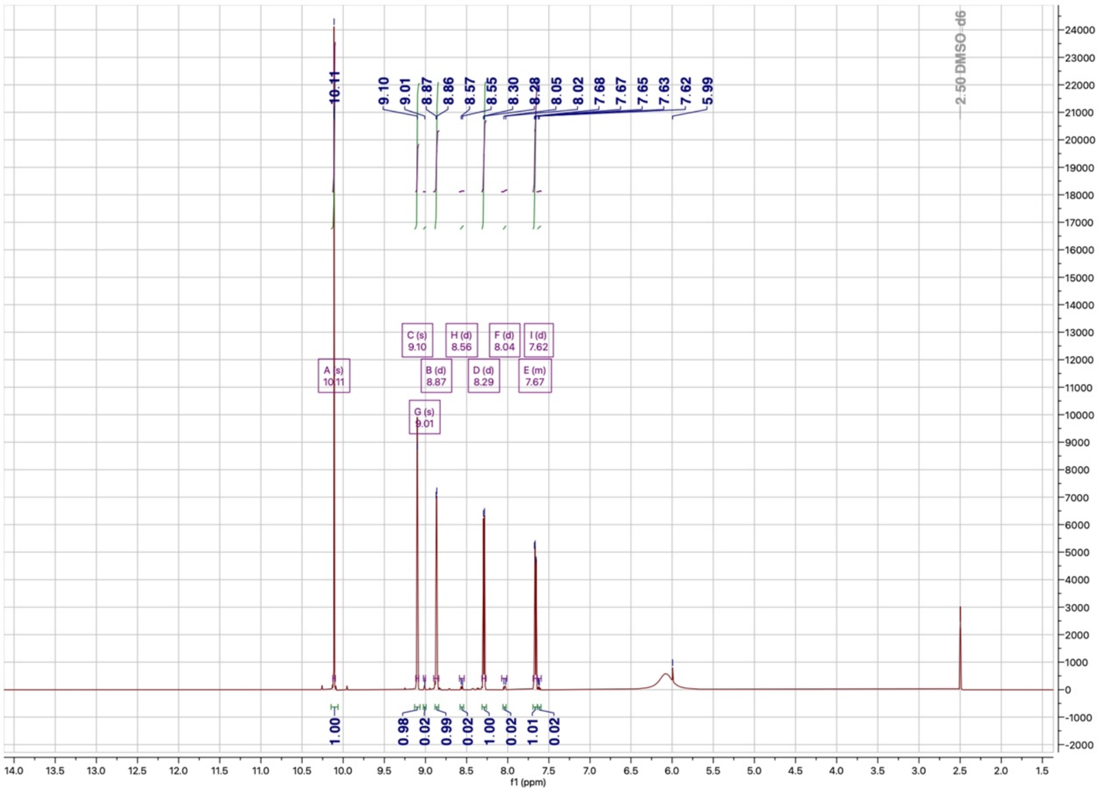

Aldehyde: δ 10.11 (s, -C**H**O), 9.10 (s, **H**$_2$), 8.87 (d, J = 4.8 Hz, **H**$_6$), 8.29 (d, J = 7.8 Hz, **H**$_4$**)**, 7.69 – 7.64 (m, **H**$_5$**)**, *gem*-diol: 9.01 (s, **H**$_2$), 8.56 (d, J = 10.1 Hz, **H**$_6$), 8.04 (d, J = 13.7 Hz, **H**$_4$), 7.62 (d, J = 7.1 Hz, **H**$_5$), 5.59 (s, -C**H**(OH)$_2$).

Figure S35. ^{1}H-NMR spectra of **A**$_3$ in DMSO-*d*$_6$/NaOH (0.1M): 100% Aldehyde

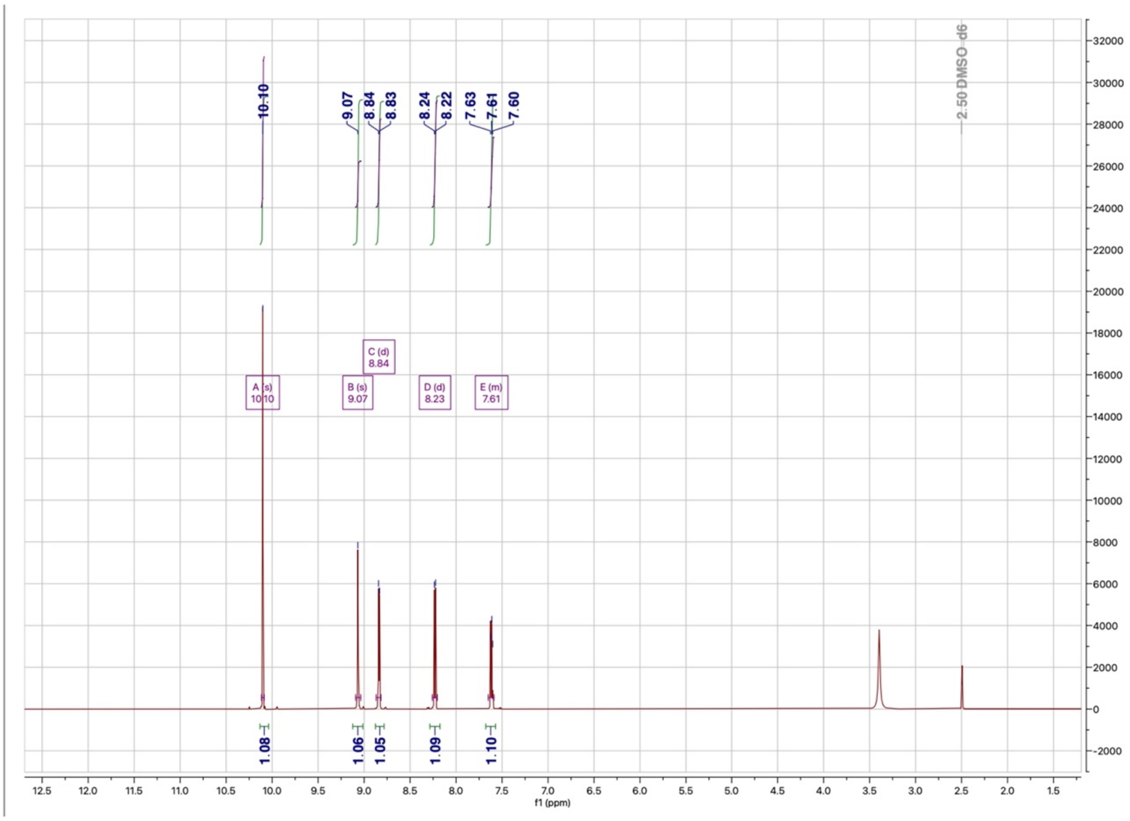

Aldehyde: δ 10.10 (s, -C**H**O), 9.07 (s, **H**$_2$), 8.84 (d, *J* = 6.7 Hz, **H**$_6$), 8.23 (d, *J* = 7.9 Hz, **H**$_4$), 7.65 – 7.59 (m, **H**$_5$).

Figure S36. ^{1}H-NMR spectra of $\mathbf{B_1}$ in CD_3OD: 55% Aldehyde, 45% hemiacetal.

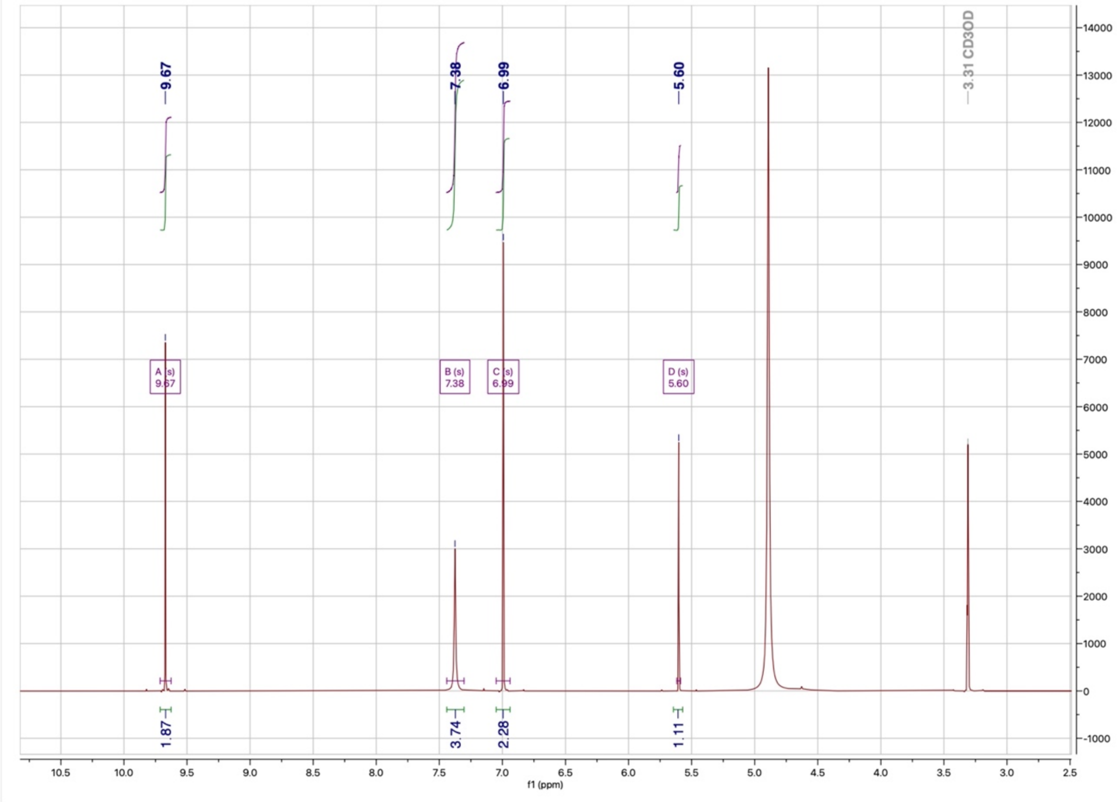

Aldehyde: δ 9.67 (s, -C**H**O), 7.38 (s, $\mathbf{H_{4,5}}$); hemiacetal: 6.99 (s, $\mathbf{H_{4,5}}$), 5.60 (s, -C**H**(OD)(OCD_3)).

Figure S37. ^{1}H-NMR spectra of $\mathbf{B_1}$ in CD_3OD/TFA: 100% hemiacetal

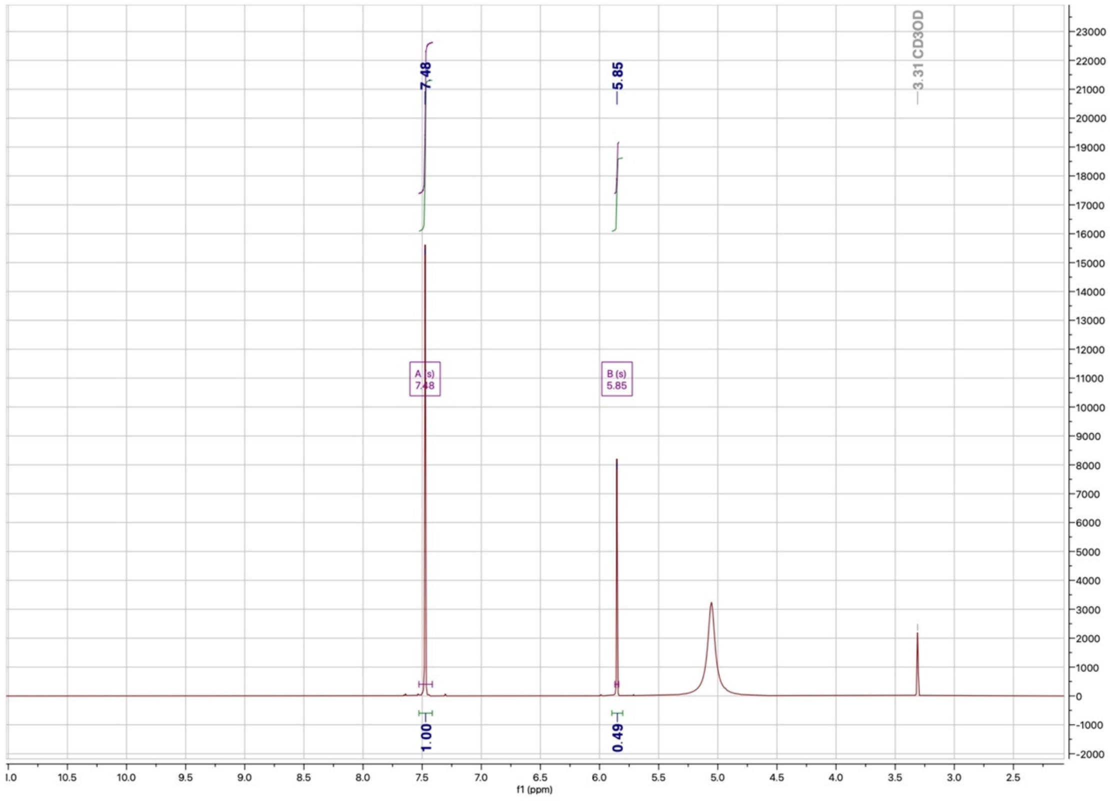

Hemiacetal: 7.48 (s, $\mathbf{H_{4,5}}$), 5.85 (s, -C**H**(OD)(OCD_3)).

Figure S38. ^{1}H-NMR spectra of **B$_1$** in CD_3OD/NaOH (0.1M): 100% Aldehyde

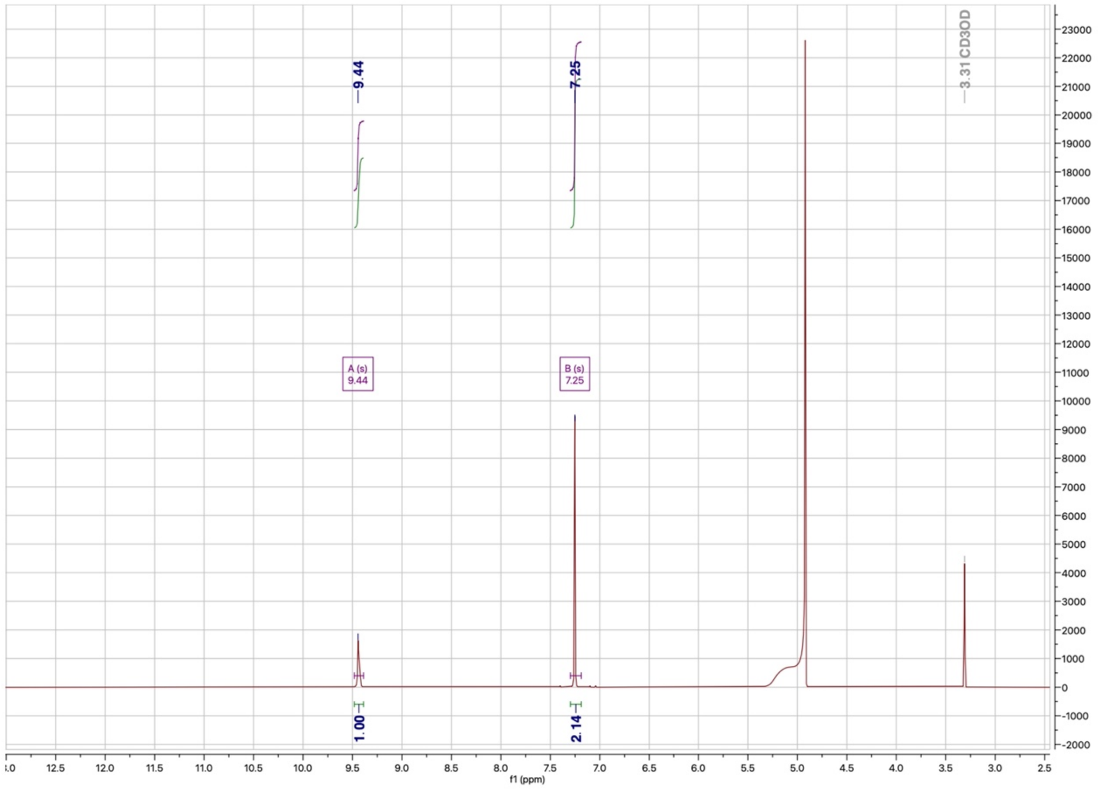

Aldehyde: δ 9.44 (s, -C**H**O), 7.25 (s, **H$_{4,5}$**).

Figure S39. ^{1}H-NMR spectra of **B$_1$** in DMSO-*d*$_6$: 100% Aldehyde

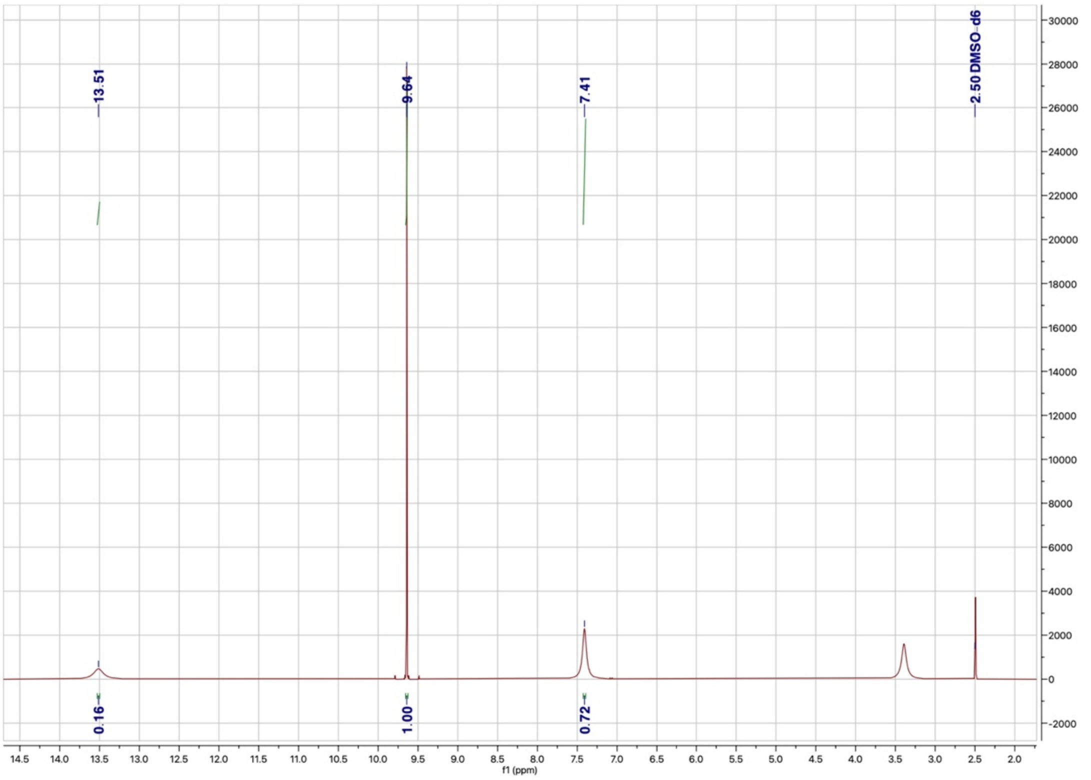

Aldehyde: δ 13.52 (s, -N**H**), 9.64 (s, -C**H**O), 7.41 (s, **H$_{4,5}$**).

Figure S40. ^{1}H-NMR spectra of **B$_1$** in DMSO-d_6/TFA: 55% *gem*-diol, 45% aldehyde

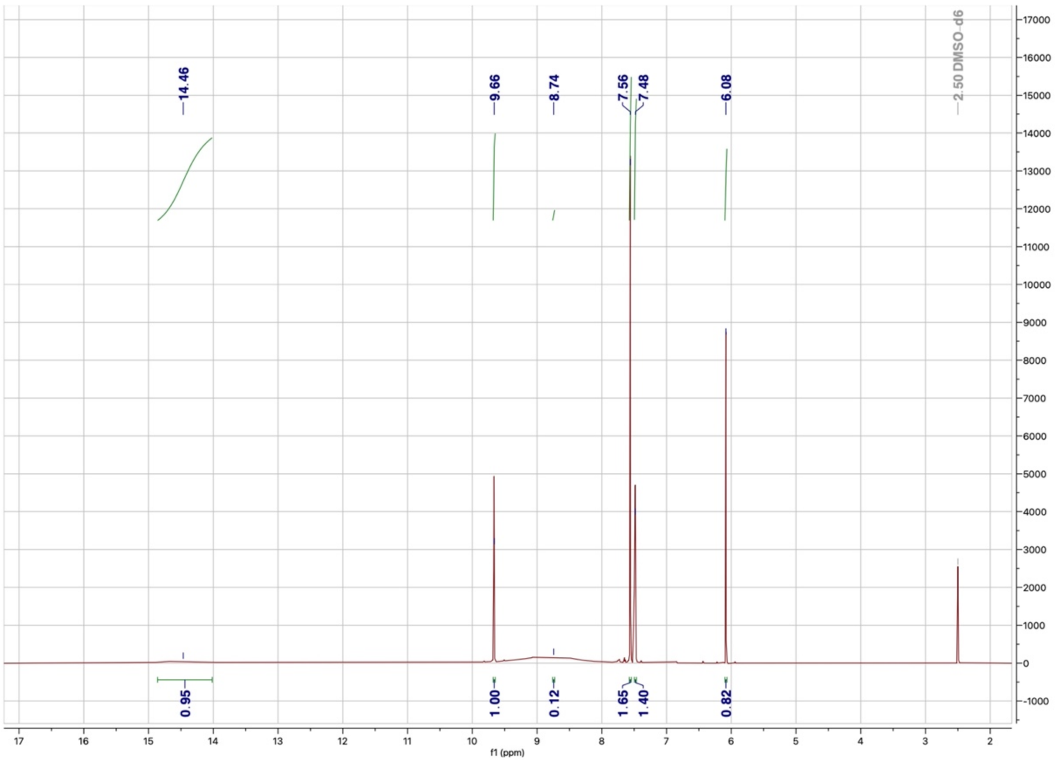

Aldehyde: δ 14.46 (s, -N**H**), 9.66 (s, -C**H**O), 7.56 (s, **H$_{4,5}$**); *gem*-diol: 8.74 (s, OH),7.48 (s, **H$_{4,5}$**), 6.08 (s, -C**H**(OH)$_2$).

Figure S41. ^{1}H-NMR spectra of **B$_1$** DMSO-d_6/NaOH (0.1M): 100% Aldehyde.

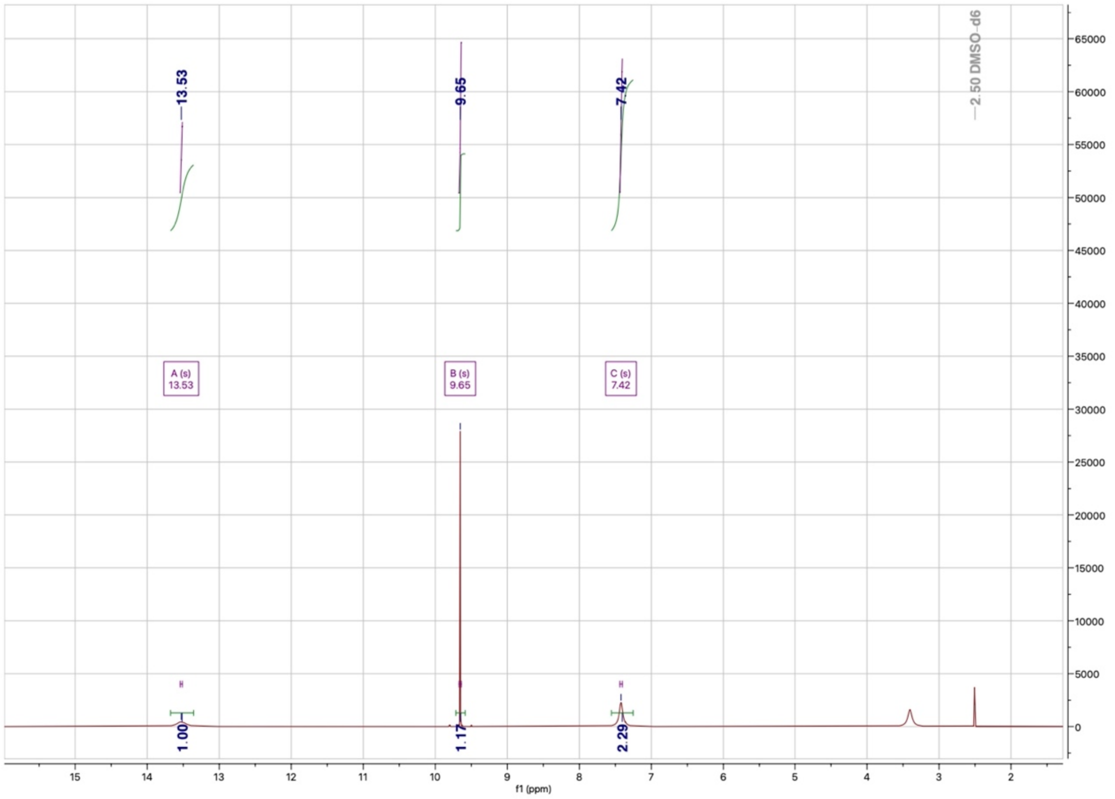

Aldehyde: δ 13.53 (s, -N**H**), 9.65 (s, -C**H**O), 7.42 (s, **H$_{4,5}$**).

Figure S42. ^{1}H-NMR spectra of **B$_2$** in D_2O/NaOH (0.1M): 100% Aldehyde

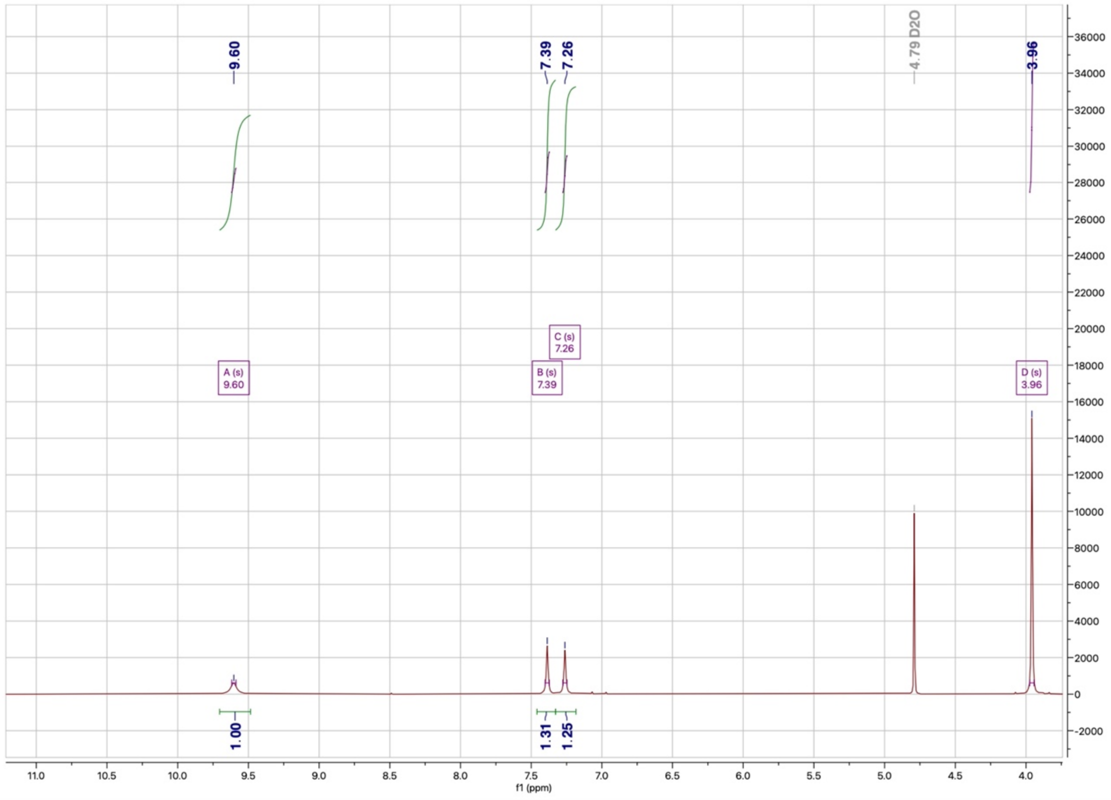

Aldehyde: δ 9.60 (s, -C**H**O), 7.39 (s, **H$_4$**), 7.26 (s, **H$_5$**), 3.96 (s, -C**H$_3$**).

Figure S43. ^{1}H-NMR spectra of **B$_2$** in CD_3OD: 64% Aldehyde, 36% hemiacetal.

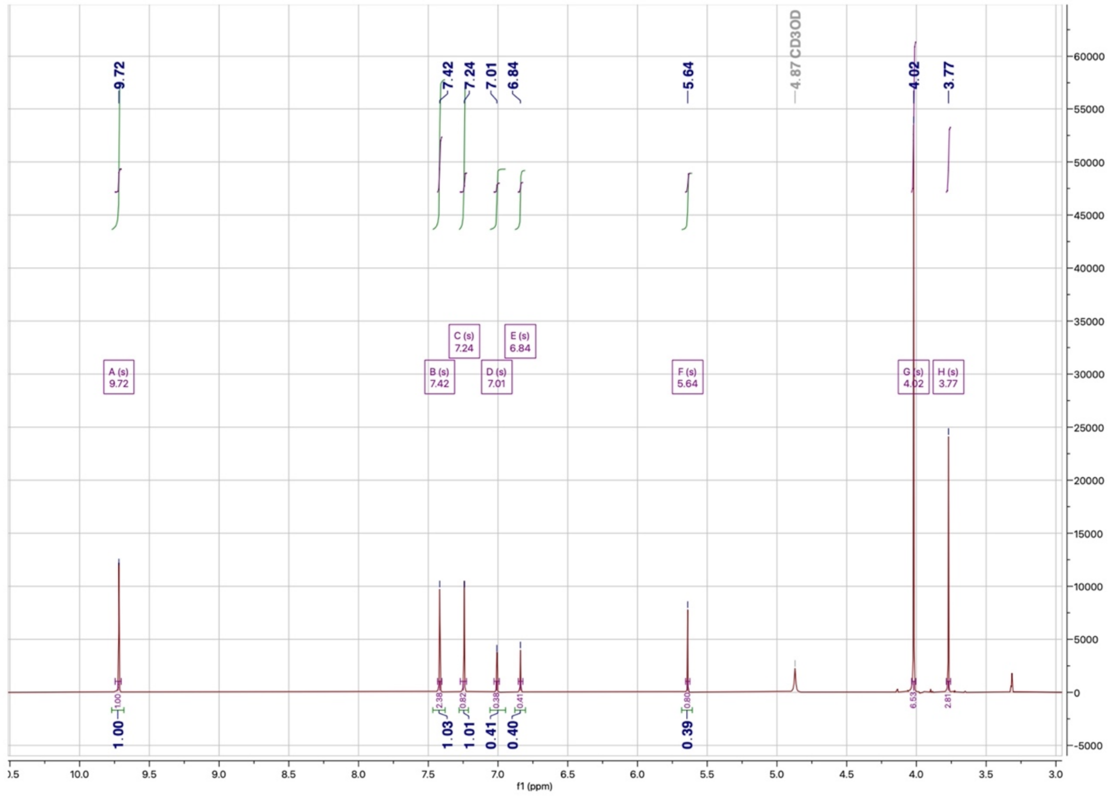

Hemiacetal: δ 7.42 (s, **H$_5$**), 7.24 (s, **H$_4$**), 5.64 (s, -C**H**(OH)(OCD_3)), 4.02 (s, -C**H$_3$**); Aldehyde: 9.72 (s, -C**H**O), 7.01 (s, **H$_5$**), 6.84 (s, **H$_4$**), 3.77 (s, -C**H$_3$**).

Figure S44. ^{13}C-NMR spectra of **B_2** in CD_3OD: 64% Aldehyde, 36% Hemiacetal

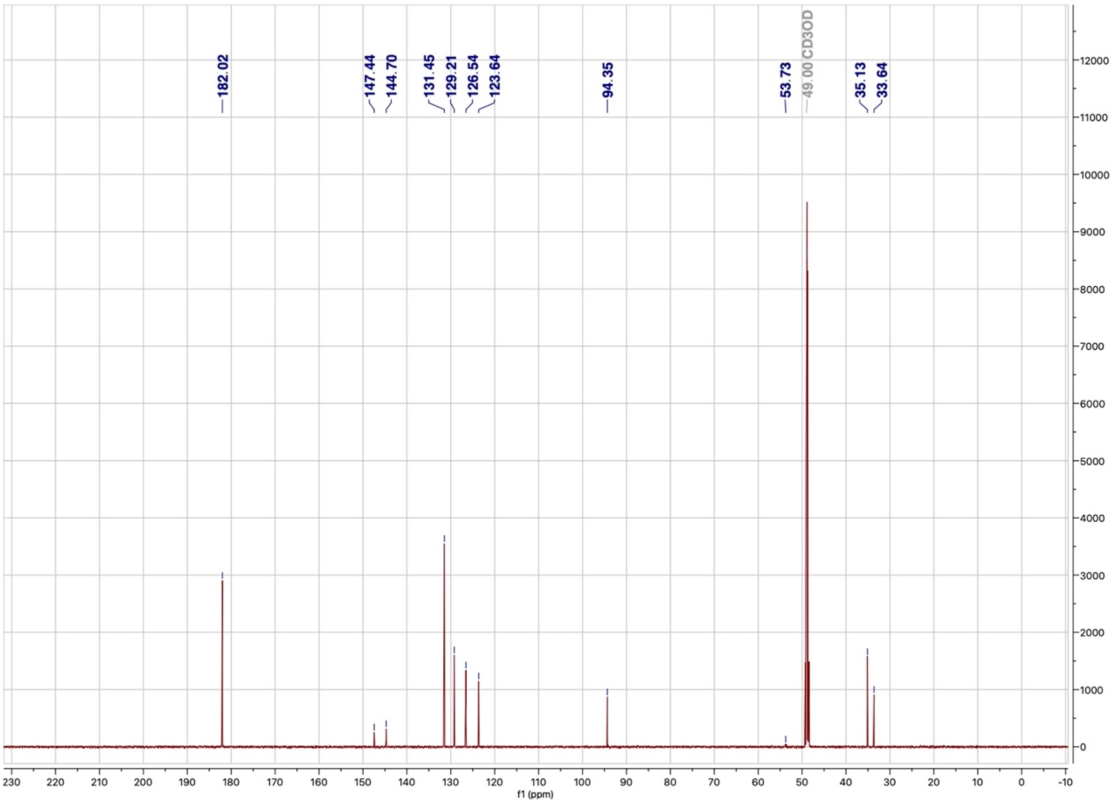

Hemiacetal: δ 147.44 (**C_2**), 131.45 (**C_5**), 123.64 (**C_4**), 94.35 (-**C**H(OH)(OCD_3)), 53.73 (-O**C**D_3), 35.13 (-**C**H_3); Aldehyde: 182.02 (-**C**HO), 144.70 (**C_2**), 129.21 (**C_5**), 126.54 (**C_4**), 33.64 (-**C**H_3).

Figure S45. ^{1}H-NMR spectra of **B_2** in CD_3OD/TFA: 34% Aldehyde, 66% hemiacetal

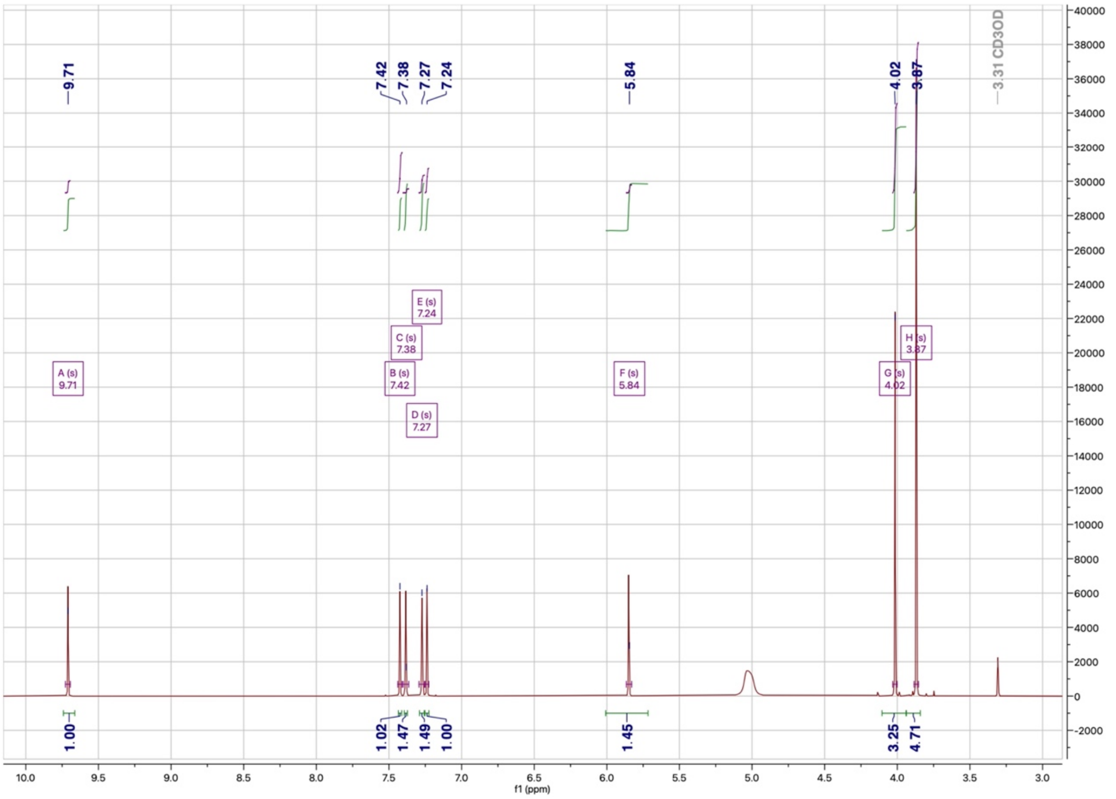

Aldehyde: δ 9.71 (s, -C**H**O), 7.42 (s, **H_4**), 7.24 (s, **H_5**), 4.02 (s, -C**H_3**); Hemiacetal: 7.38 (s, **H_4**), 7.27 (s, **H_5**), 5.84 (s, -C**H**(OH)(OCD_3)) 3.87 (s, -C**H_3**).

Figure S46. ^{1}H-NMR spectra of **B₂** in CD_3OD/NaOH (0.1M): 4% Aldehyde, 36% hydroxymethyl, 60% carboxylic acid.

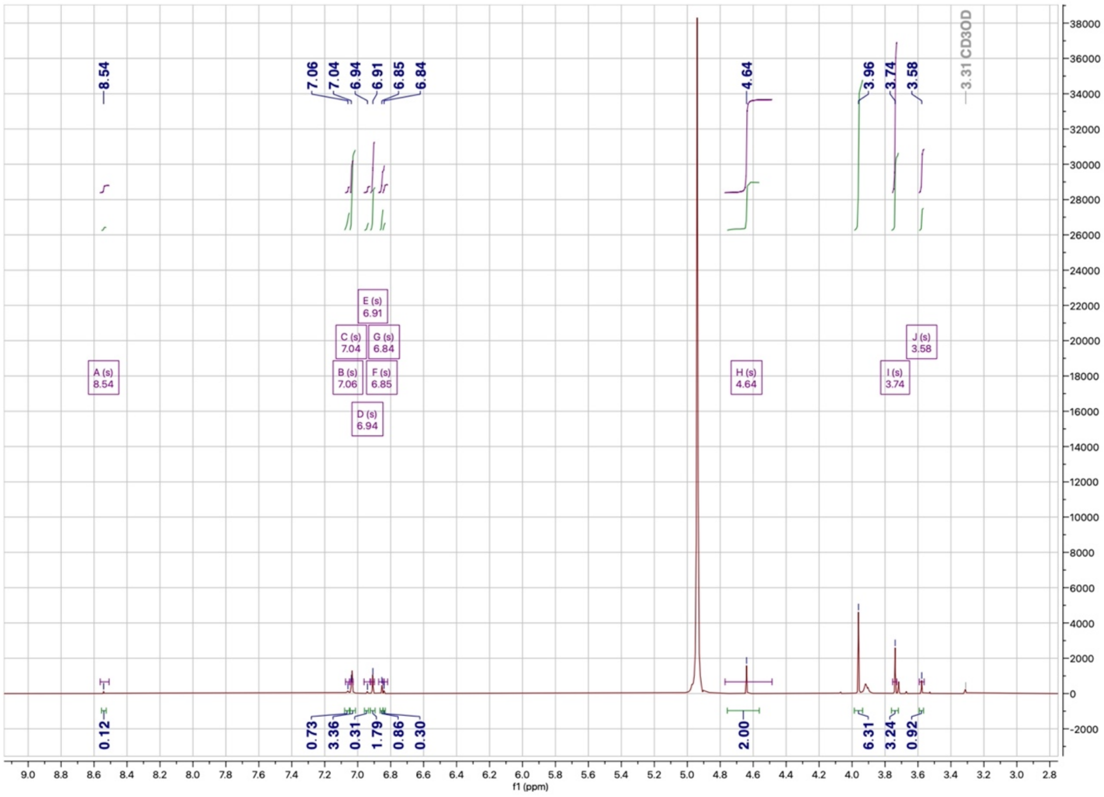

Aldehyde: δ 8.54 (s, -C**H**O), 7.06 (s, **H_5**), 6.94 (s, **H_4**), 3.58 (s, -C**H_3**). Carboxylic acid: 7.04 (s, **H_4**), 6.91 (s, **H_5**), 3.96 (s, -C**H_3**); Hydroxymethyl: 6.85 (s, **H_4**), 6.84 (s, **H_5**), 4.64 (s, -C**H_2**OH), 3.74 (s, -C**H_3**),

Figure S47. ^{13}C-NMR spectra of **B_2** in CD_3OD/NaOH (0.1M): 4% Aldehyde, 36% hydrohymethyl, 60% sodium carboxylate.

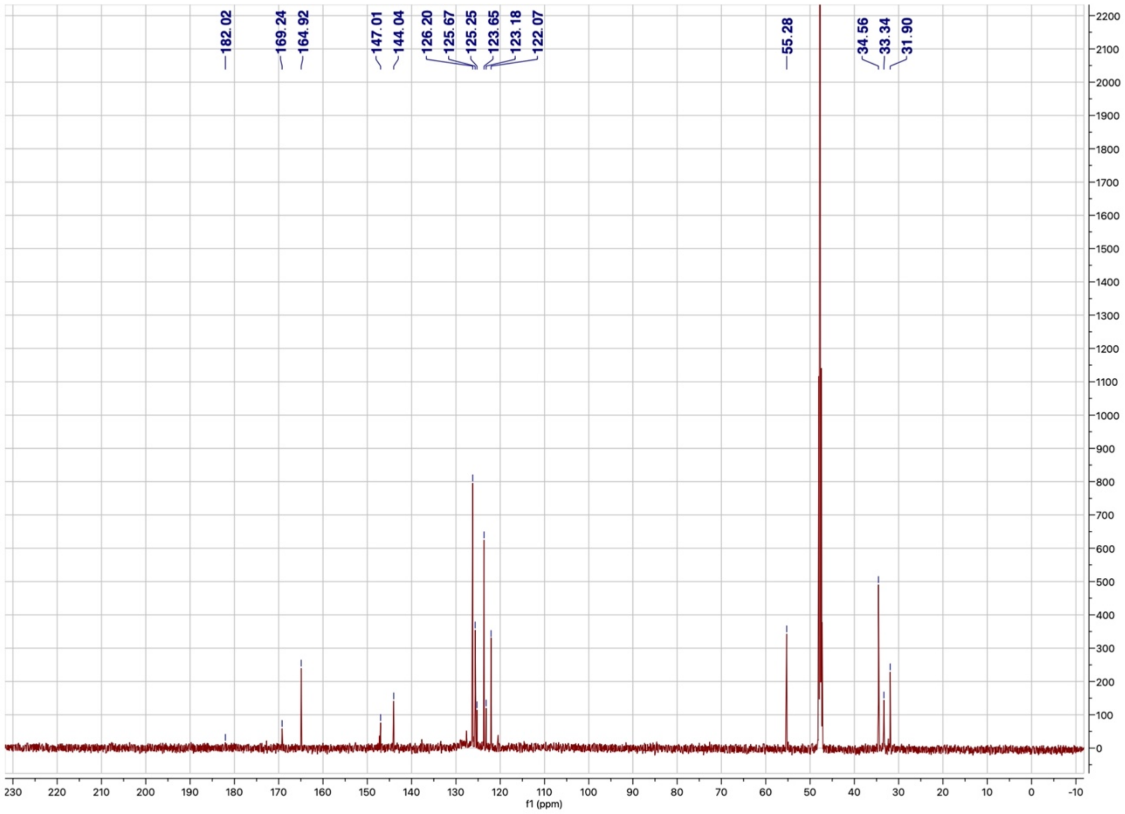

Aldehyde: δ 182.02 (CHO), 147.01 (C_4), 144.04 (C_2) ,125.25 (C_5), 33.34 ($-CH_3$); sodium carboxylate: 169.24 (COONa), 164.92 (C_2), 125.67 (C_4), 123.65 (C_5), 34.56 (CH_3); Hydroxymethyl: 126.20 (C_4), 125.67 (C_2), 123.65 (C_5), 55.28 (CH_2OH), 31.90 (CH_3). The unassigned signals belong to the hemiacetal moiety that represents less than 0.1%.

Figure S48. ^{1}H-NMR spectra of **B$_2$** in DMSO-d_6: 100% Aldehyde.

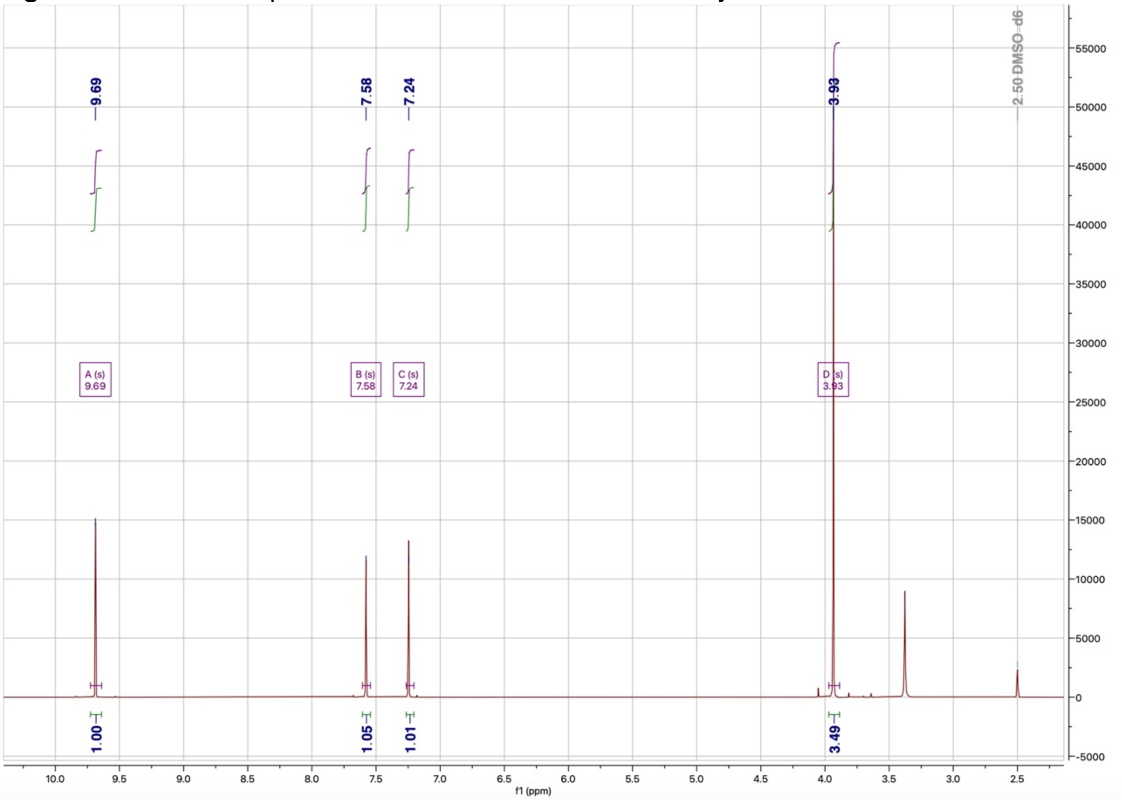

Aldehyde: δ 9.69 (s, -C**H**O), 7.58 (s, **H$_4$**), 7.24 (s, **H$_5$**), 3.93 (s, -C**H$_3$**).

Figure S49. ^{1}H-NMR spectra of **B$_2$** in DMSO-d_6/TFA: 67% Aldehyde, 33% *gem*-diol

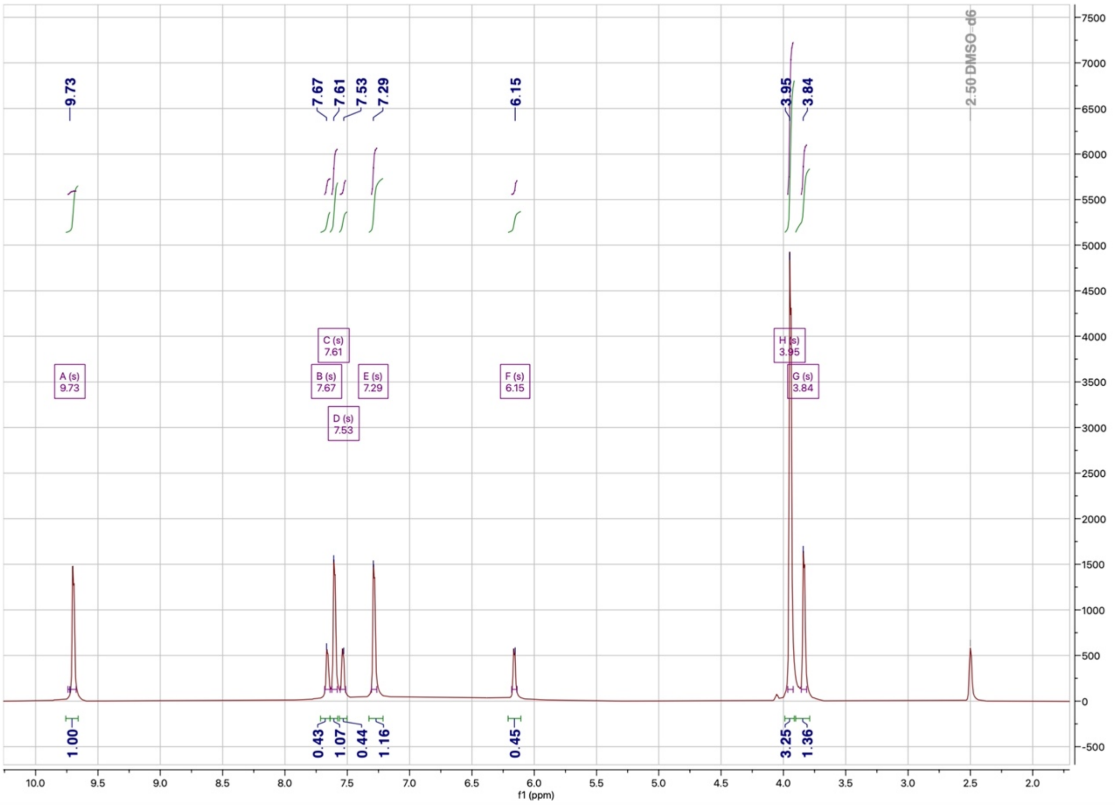

Aldehyde: δ 9.73 (s, -C**H**O), 7.61 (s, **H$_4$**), 7.29 (s, **H$_5$**), 3.95 (s, -C**H$_3$**); *gem*-diol: 7.67 (s, **H$_4$**), 7.53 (s, **H$_5$**), 6.15 (s, -C**H**(OH)$_2$), 3.84 (s, -C**H$_3$**).

Figure S50. ^{1}H-NMR spectra of **B_2** in DMSO-d_6/NaOH (0.1M): 65% Aldehyde, 19% hydroxymethyl and 16% carboxylic acid.

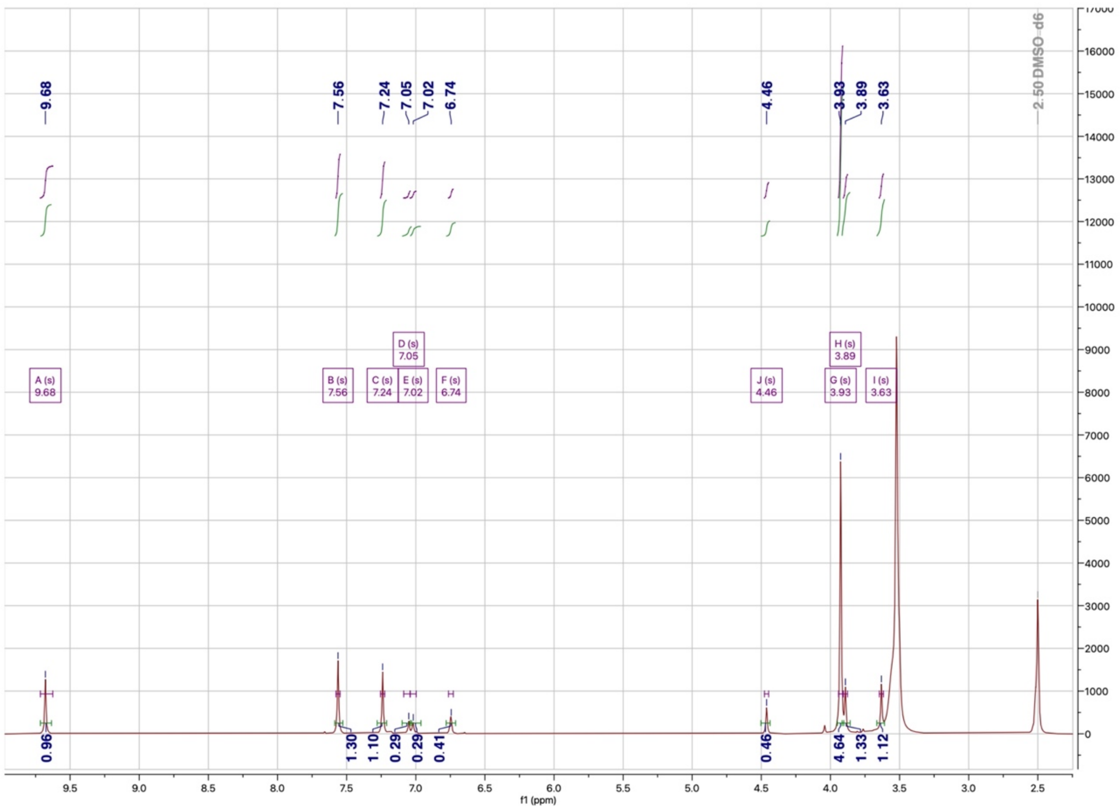

Aldehyde: δ 9.68 (s, -C**H**O), 7.56 (s, **H_4**), 7.24 (s, **H_5**), 3.93 (s, -C**H_3**); hydroxymethyl: 7.02 (s, **H_4**), 6.74 (s, **H_5**), 4.46 (s, -C**H_2**OH), 3.89 (s, -CH_3), carboxylic acid: 7.05 (s**, H_4**), 3.63 (s, -CH_3).

Figure S51. ^{13}C-NMR spectra of **B₂** in DMSO-d_6/NaOH (0.1M): 65% Aldehyde, 19% hydroxymethyl and 16% carboxylic acid.

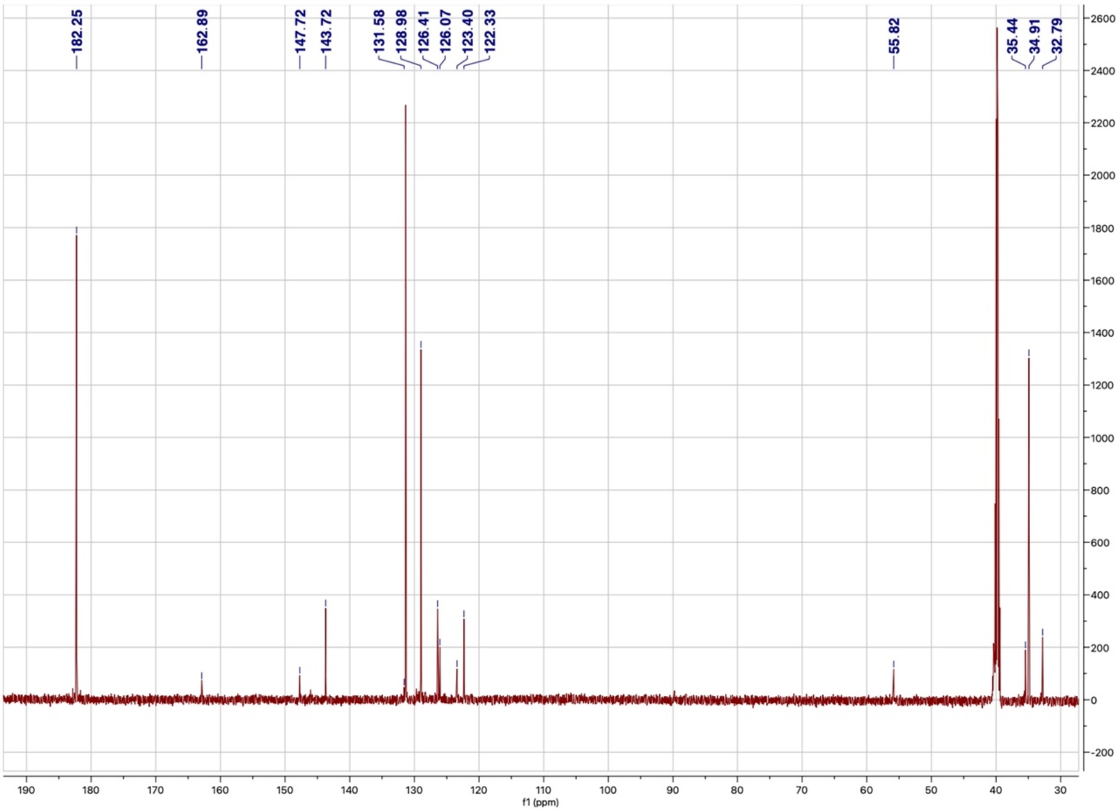

Aldehyde: 182.25 (CHO); sodium carboxylate: 162.89 (COONa); hydroxymethyl: 55.82 (CH_2OH)

Figure S52. ^{1}H-NMR spectra of **B_4** in D_2O/NaOH (0.1M): 100% Aldehyde

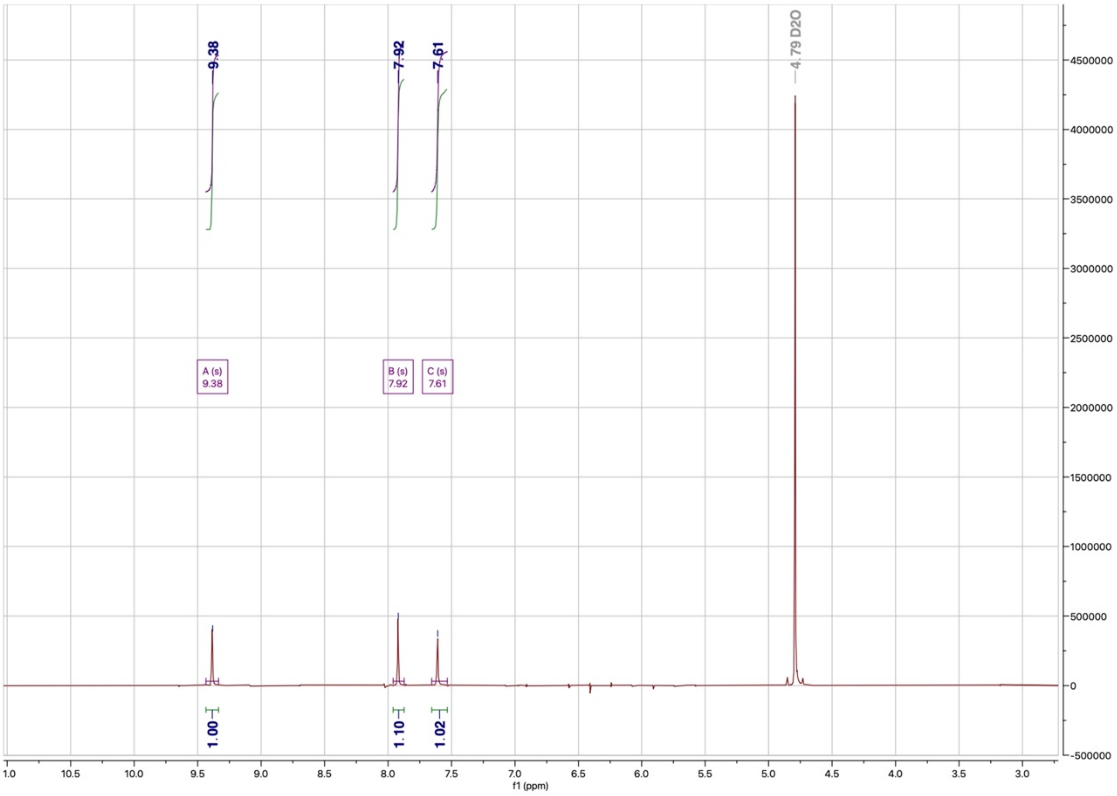

Aldehyde: δ 9.38 (s, -C**H**O), 7.92 (s, **H_2**), 7.61 (s, **H_5**).

Figure S53. ^{1}H-NMR spectra of **B$_4$** in CD_3OD: 96% Aldehyde, 4% hemiacetal

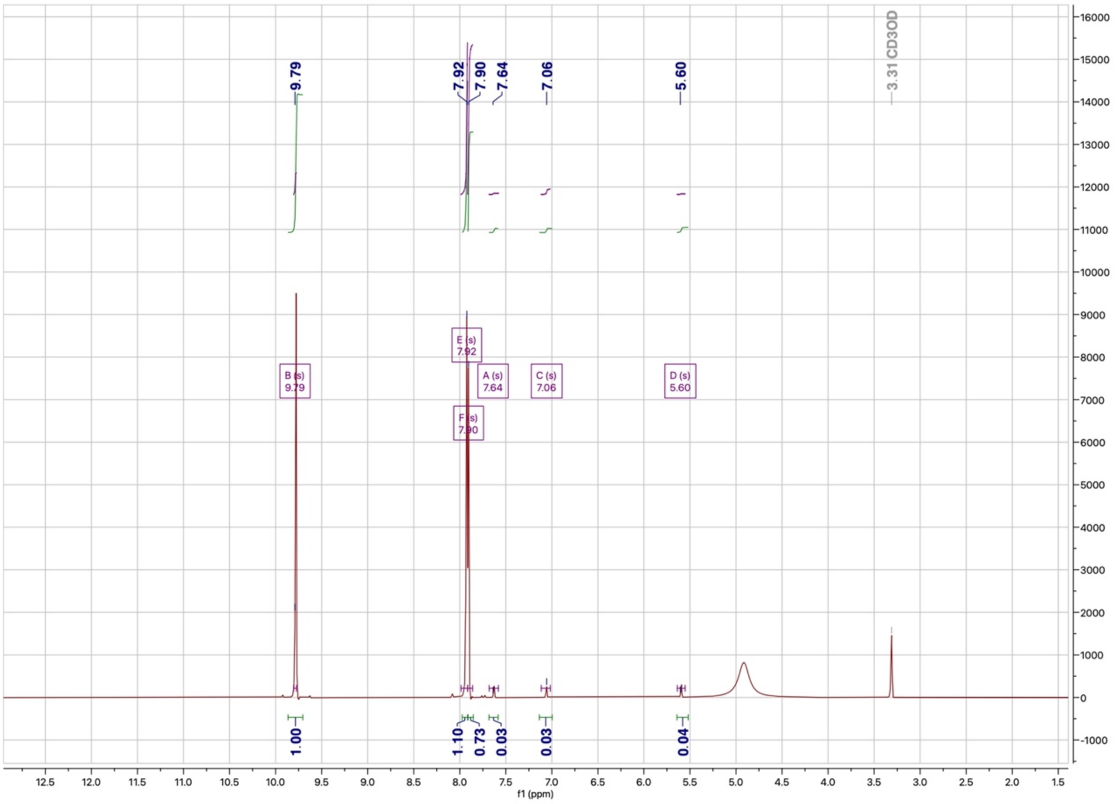

Aldehyde: δ 9.79 (s, -C**H**O), 7.92 (s, **H$_2$**), 7.90 (s, **H$_5$**); hemiacetal: 7.64 (s, **H$_2$**), 7.06 (s, **H$_5$**) 5.60 (s, -C**H**(OH)(OCD_3)).

Figure S54. ^{1}H-NMR spectra of **B$_4$** in CD_3OD/TFA: 15% Aldehyde, 85% hemiacetal

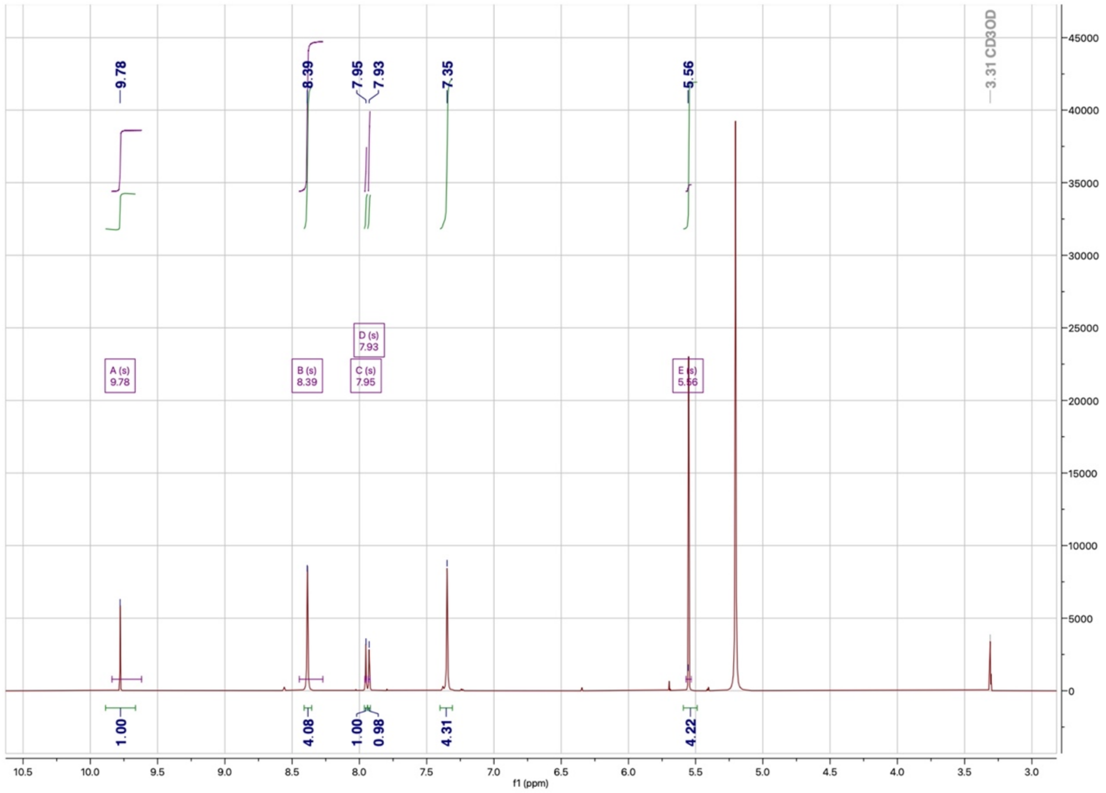

Aldehyde: δ 9.78 (s, -C**H**O), 7.95 (s, **H**$_2$), 7.93 (s, **H**$_5$); hemiacetal: 8.39 (s, **H**$_2$), 7.35 (s, **H**$_5$) 5.56 (s, -C**H**(OH)(OCD_3)).

Figure S55. ^{13}C-NMR spectra of **B**$_4$ in CD_3OD/TFA: 15% Aldehyde, 85% hemiacetal

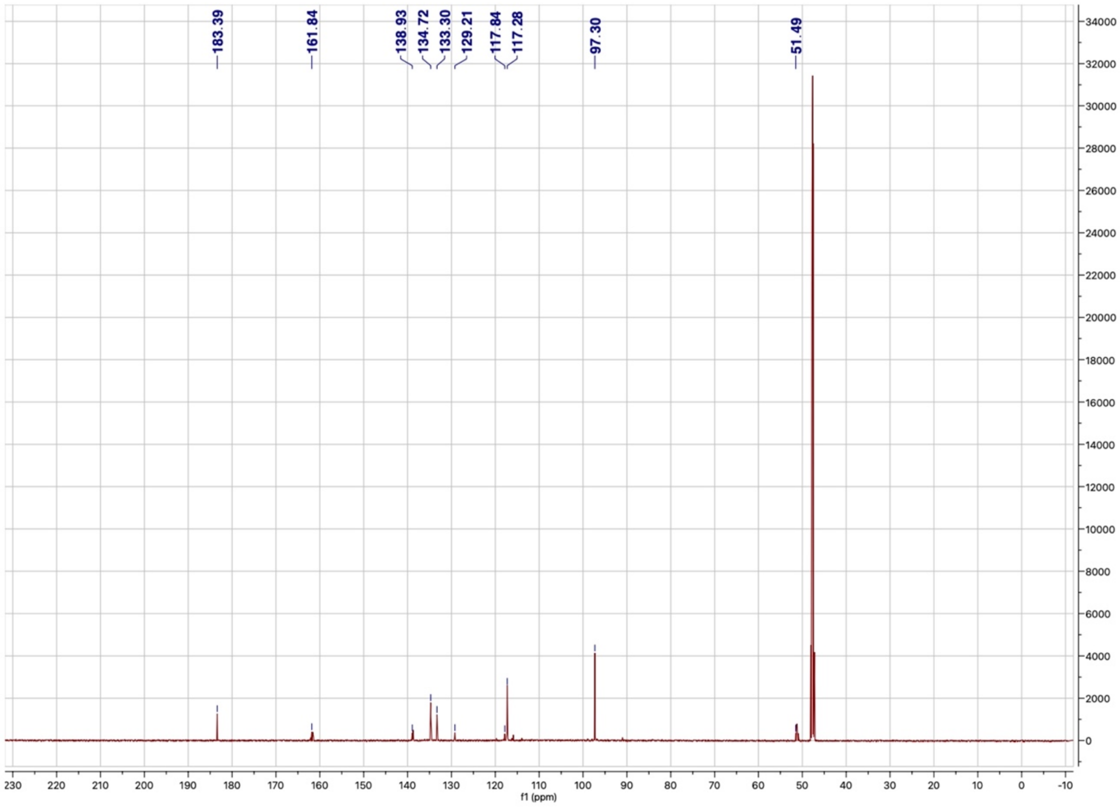

Aldehyde: δ 183.39 (-**C**HO), 138.93 (**C**$_2$), 129.21(**C**$_3$), 117.84 (**C**$_5$); hemiacetal: 134.72 (**C**$_2$), 133.30 (**C**$_3$), 117.28 (**C**$_5$), 97.30 (-**C**H(OH)(OCD_3)), 51.49 (-CH(OH)(O**C**D_3)). 161.84 (TFA).

Figure S56. ^{1}H-NMR spectra of B_4 in CD_3OD/NaOH (0.1M): 96% Aldehyde, 4% hemiacetal

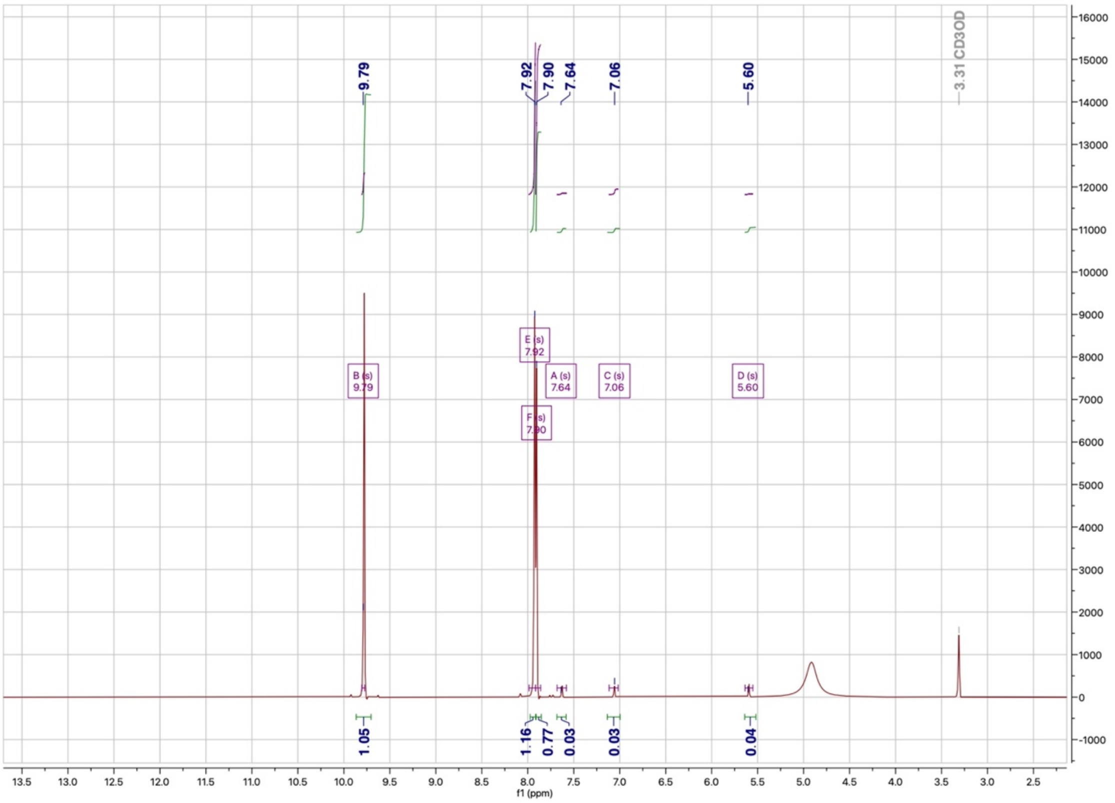

Aldehyde: δ 9.79 (s, -C**H**O), 7.92 (s, $\mathbf{H_2}$), 7.90 (s, $\mathbf{H_5}$); hemiacetal: 7.64 (s, $\mathbf{H_2}$), 7.06 (s, $\mathbf{H_5}$) 5.60 (s, -C**H**(OH)(OCD_3)).

Figure S57. ^{1}H-NMR spectra of B_4 in DMSO-*d*$_6$: 100% Aldehyde

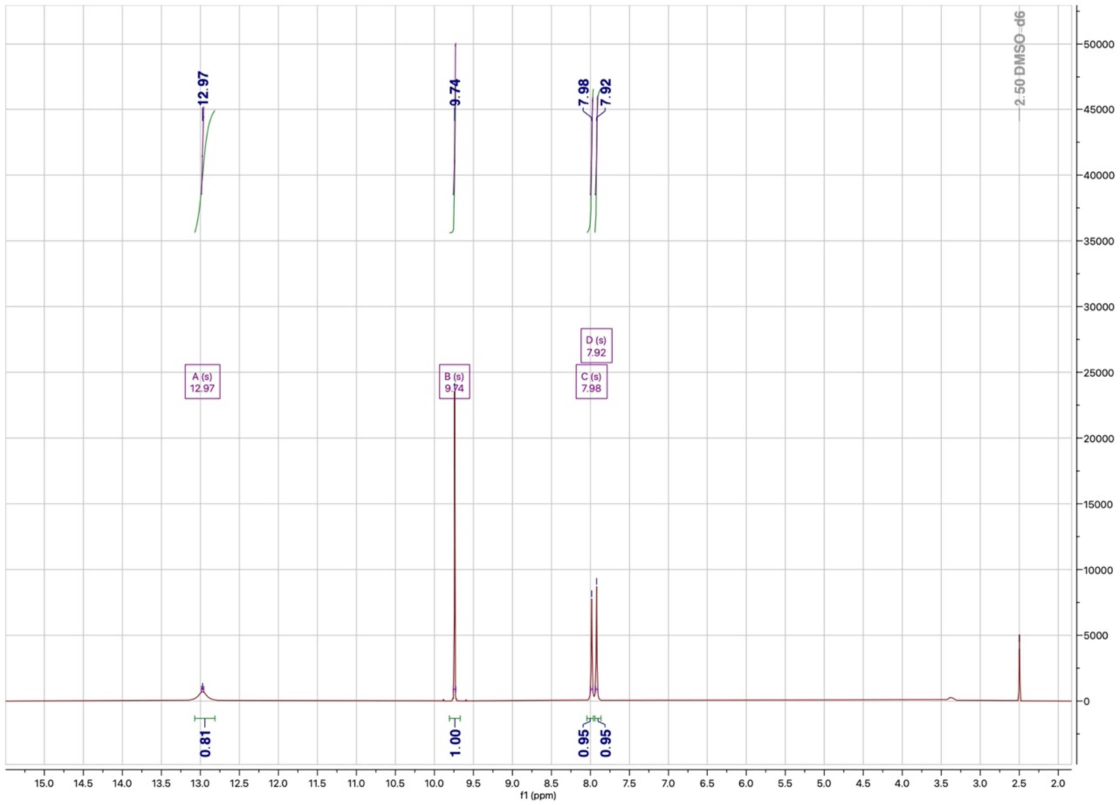

Aldehyde: δ 12.97 (-N**H**), 9.74 (s, -C**H**O), 7.98 (s, $\mathbf{H_2}$), 7.92 (s, $\mathbf{H_5}$).

Figure 58. ^{1}H-NMR spectra of **B₄** in DMSO-*d₆*/TFA: 100% Aldehyde.

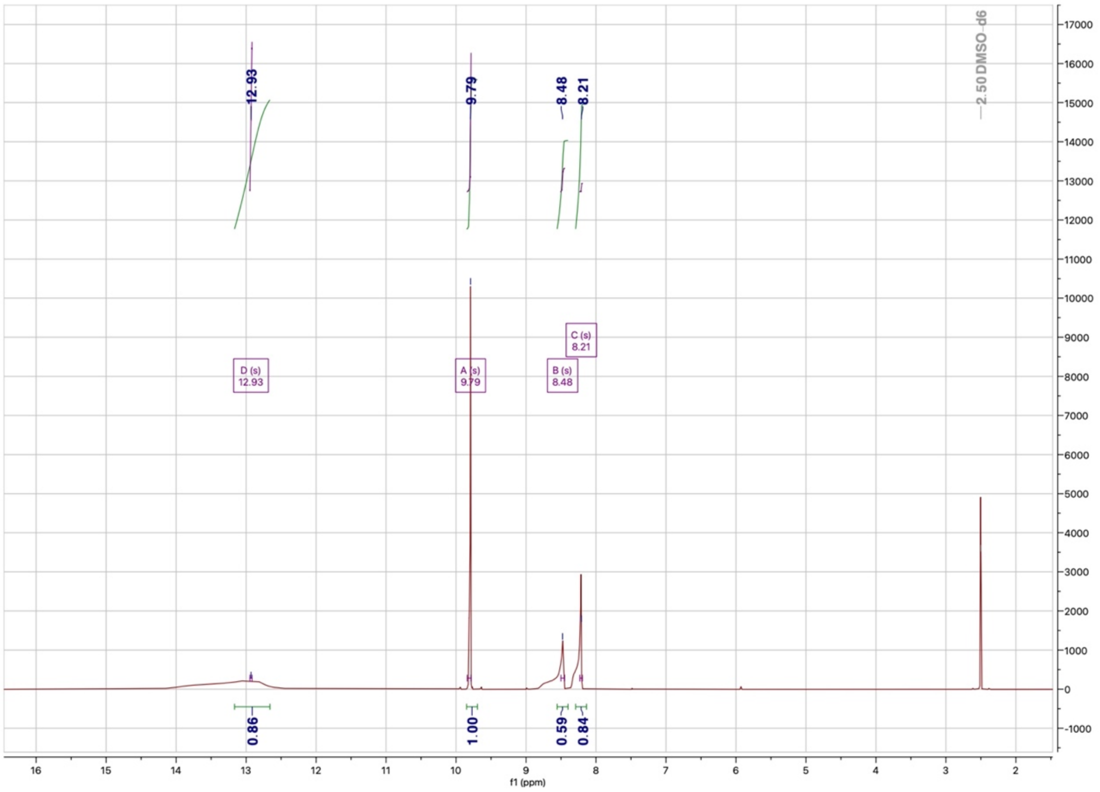

Aldehyde: δ 12.97 (-N**H**), 9.79 (s, -C**H**O), 8.48 (s, $\mathbf{H_2}$), 8.21 (s, $\mathbf{H_5}$). Unassigned peaks correspond to satellite signals and impurities that represent less than 1% in the sample.

Figure 59. ^{1}H-NMR spectra of $\mathbf{B_4}$ in DMSO-d_6/NaOH (0.1M): 100% Aldehyde

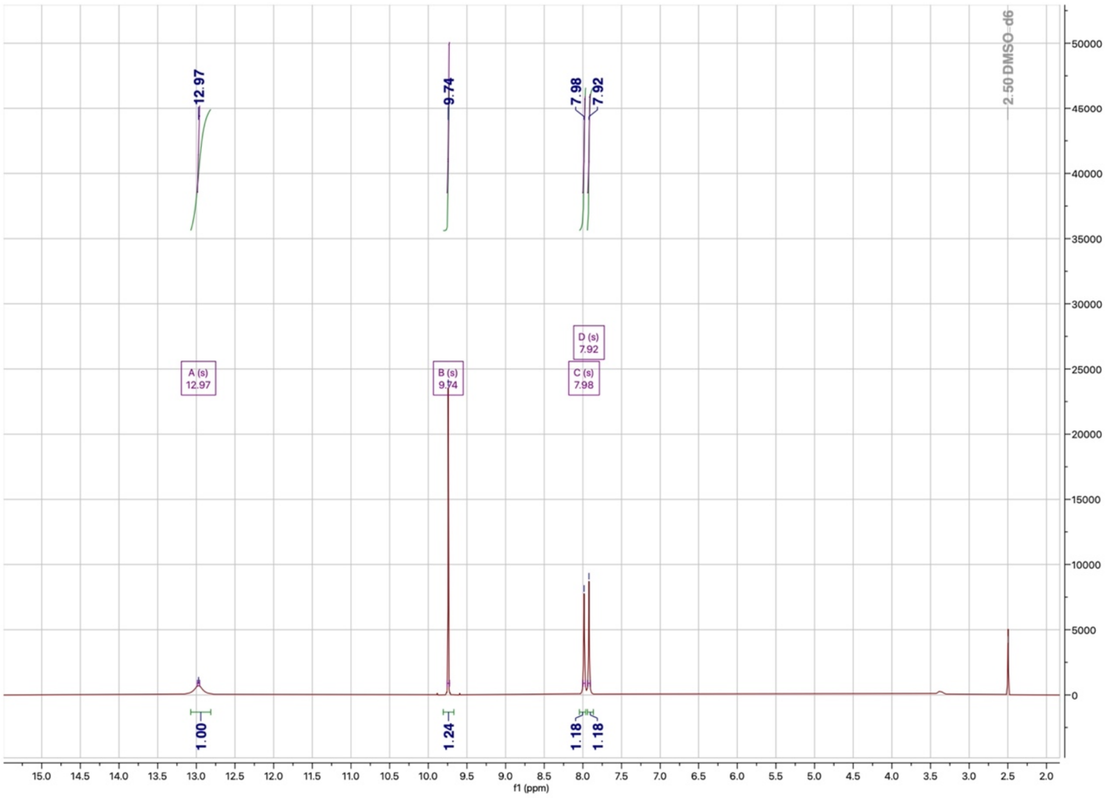

Aldehyde: δ 12.97 (-N**H**), 9.74 (s, -C**H**O), 7.98 (s, $\mathbf{H_2}$), 7.92 (s, $\mathbf{H_5}$).

Figure 60. ^{1}H-NMR spectra of **A_4** in CD_3OD/NaOH (0.4M): 81% hemiacetal, 5% hydroxymethyl and 14% carboxylic acid.

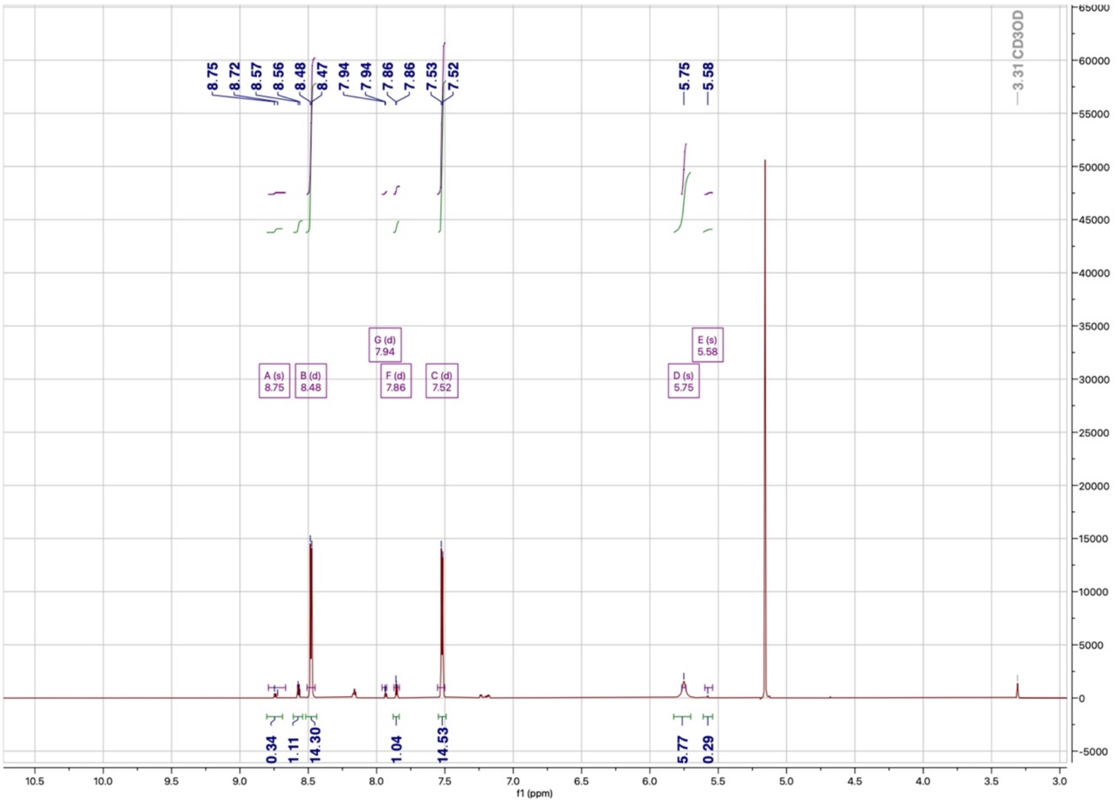

Hemiacetal: δ 7.94 (d, J = 1.6 Hz, **$H_{2,6}$**), 7.52 (d, J = 6.3 Hz, **$H_{3,5}$**), 5.75 (s, -CH(OH)(OCD_3)); carboxylic acid: 8.75 (s, **$H_{2,6}$**), 7.86 (d, J = 1.6 Hz, **$H_{3,5}$**); hydroxymethyl: 8.48 (d, J = 6.2 Hz, **$H_{2,6}$**), 7.94 (d, J = 1.5Hz, **$H_{3,5}$**) 5.58 (s, -C**H**($OH)_2$).

Figure 61. ^{1}H-NMR spectra of **A_4** in DMSO-*d_6*/NaOH (0.1M): 40% *gem*-diol, 60% aldehyde.

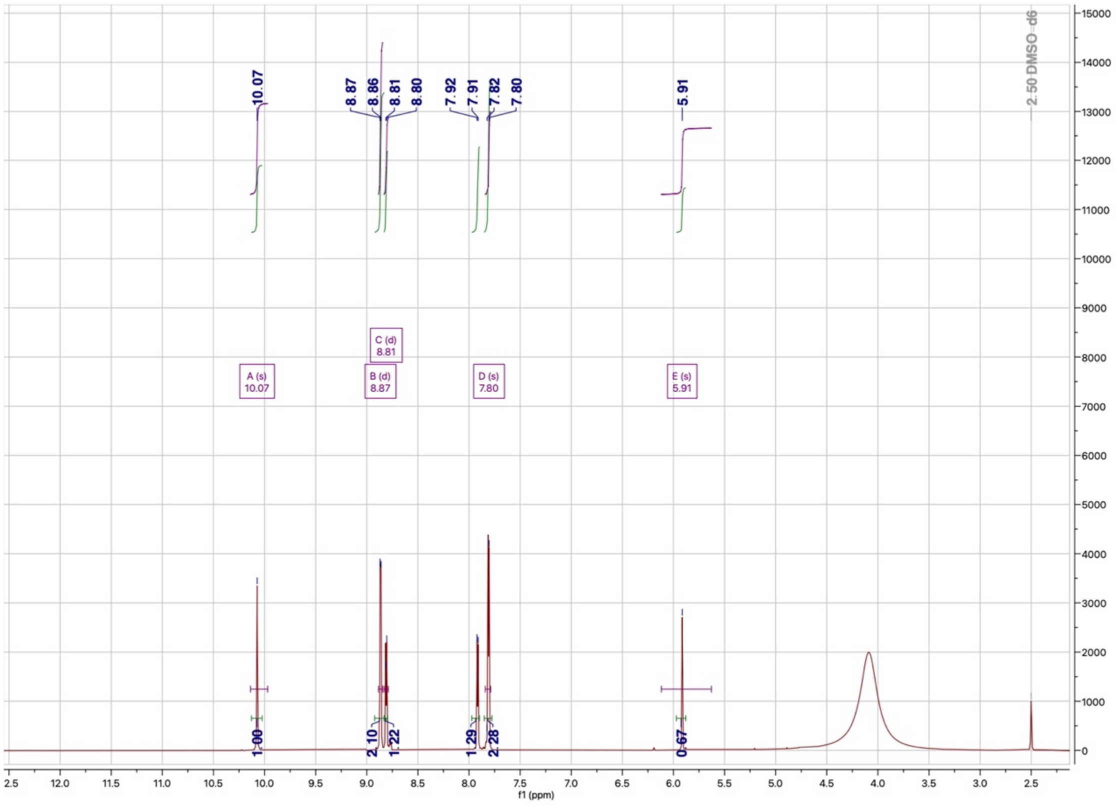

Aldehyde: δ 10.07 (s, -C**H**O), 8.86 (d, J = 4.9 Hz, **$H_{2,6}$**), 7.82 (d, J = 5.4Hz, **$H_{3,5}$**); *gem*-diol: 8.81 (d, J = 4.6 Hz, **$H_{2,6}$**), 8.00 (d, J = 5.7 Hz, **$H_{3,5}$**), 5.95 (s, -C**H**$(OH)_2$).

Figure 62. ^{1}H-NMR spectra of **A_4** in D_2O/NaOH (0.4M): 7% Aldehyde, 7% *gem*-diol, 43% hydroxymethyl and 43% sodium carboxylate.

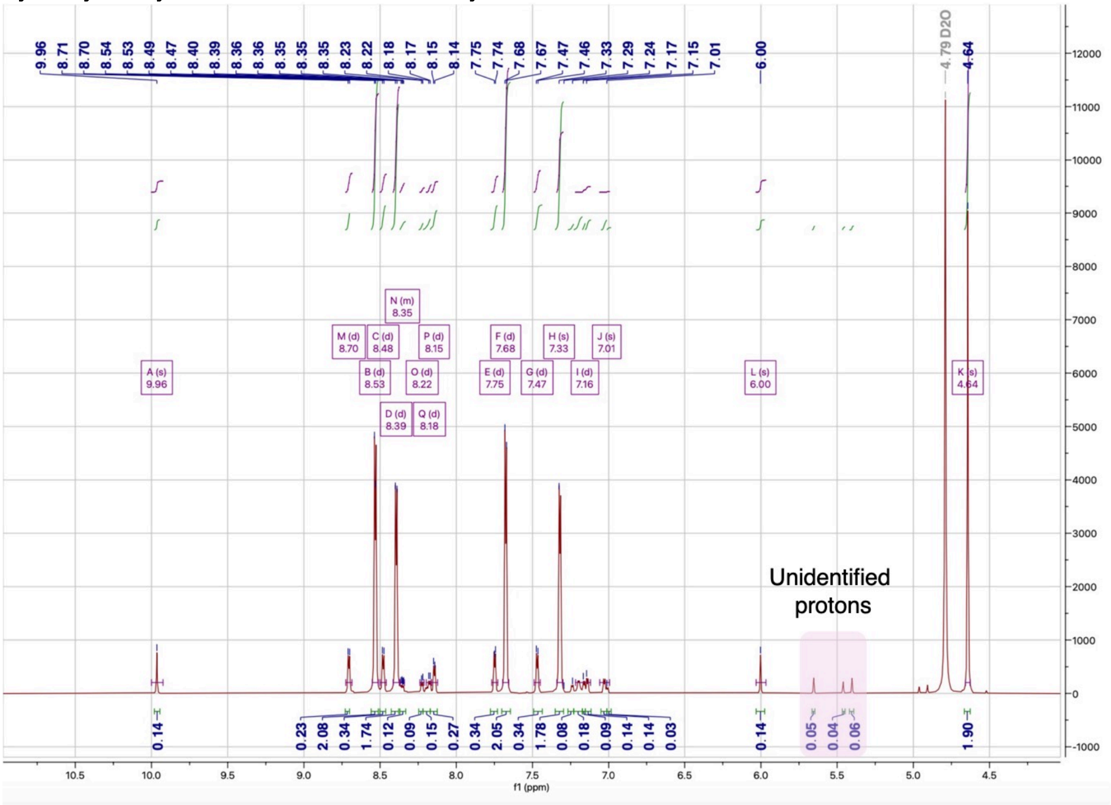

Aldehyde: δ 9.96 (s, -C**H**O), 8.48 (d, J = 6.3 Hz, **$H_{2,6}$**), 7.47 (d, J = 6.3 Hz, $H_{3,5}$); hydroxymethyl: 8.53 (d, J = 4.4 Hz, **$H_{2,6}$**), 7.75 (d, J = 4.4 Hz, $H_{3,5}$), 4.64 (s, -C**H_2**OH). *gem*-diol: 8.70 (d, J = 6.2 Hz, **$H_{2,6}$**), 7.01 (d, J = 6.2Hz, $H_{3,5}$), 6.00 (s, -C**H**$(OH)_2$),; 8.37 – 8.34 (m, 1H), 8.22 (d, J = 4.6 Hz, H2,6), 7.16 (d, J = 12.9 Hz, $H_{3,5}$); sodium carboxylate: 8.39 (d, J = 6.4 Hz, $H_{2,6}$), 7.33 (d, J = 6.2 Hz, $H_{3,5}$). The percentages reported were taken considering the four species identified. Signals marked "unassigned/unidentified" represent 1.5% of the sample among all species.

Figure S63. ^{1}H-NMR spectra of **A$_3$** in D_2O/NaOH (0.4M): 51% carboxylic acid and 49% hydroxymethyl

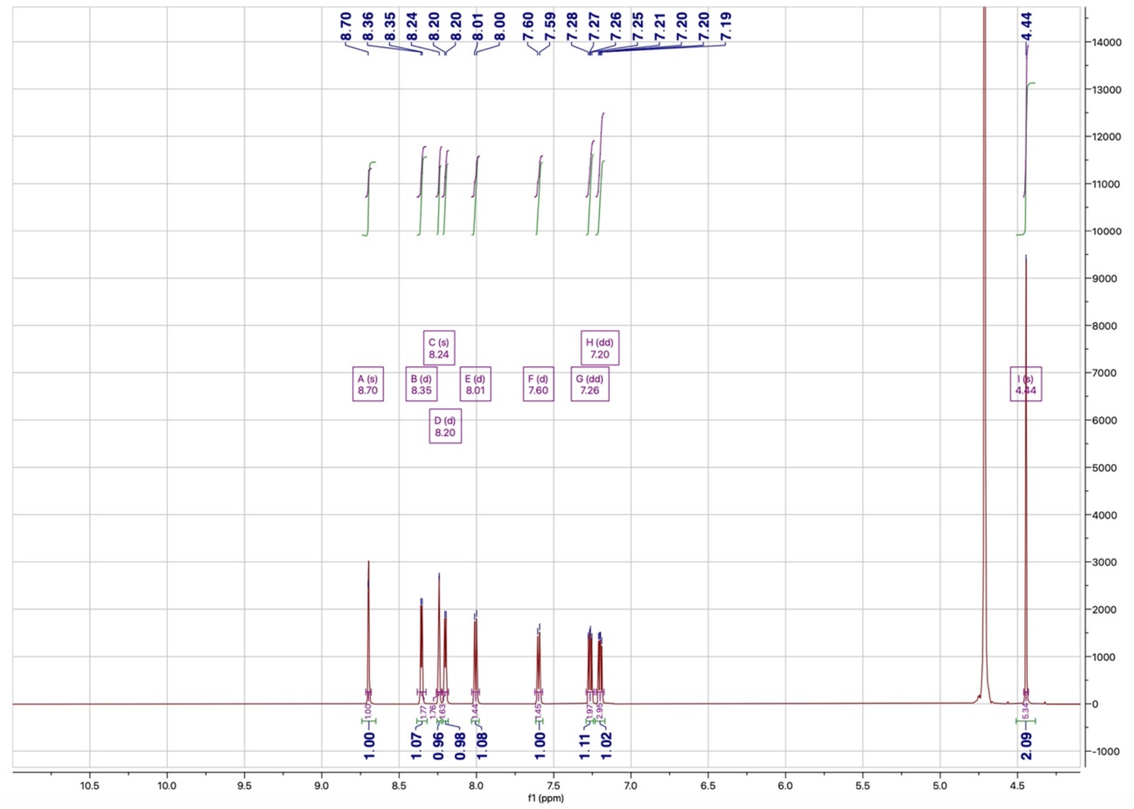

Carboxylic acid: 8.70 (s, **H$_2$**), 8.36 (**H$_6$**), 8.01 (d, J = 6.3 Hz, H$_4$), 7.26 (dd, J = 6.1Hz, J =0.5Hz, H$_5$); hydroxymethyl: 8.24 (s, H$_2$), 8.20 (d, J= 6.1Hz, H$_6$), 7.60 (d, J = 6.2Hz, H$_4$), 7.19 (dd, J = 6.1Hz, J =0.8Hz, H$_5$)

Figure S64: ^{13}C-NMR spectra of **A_3** in D_2O/NaOH (0.4M): 51% carboxylic acid and 49% hydroxymethyl

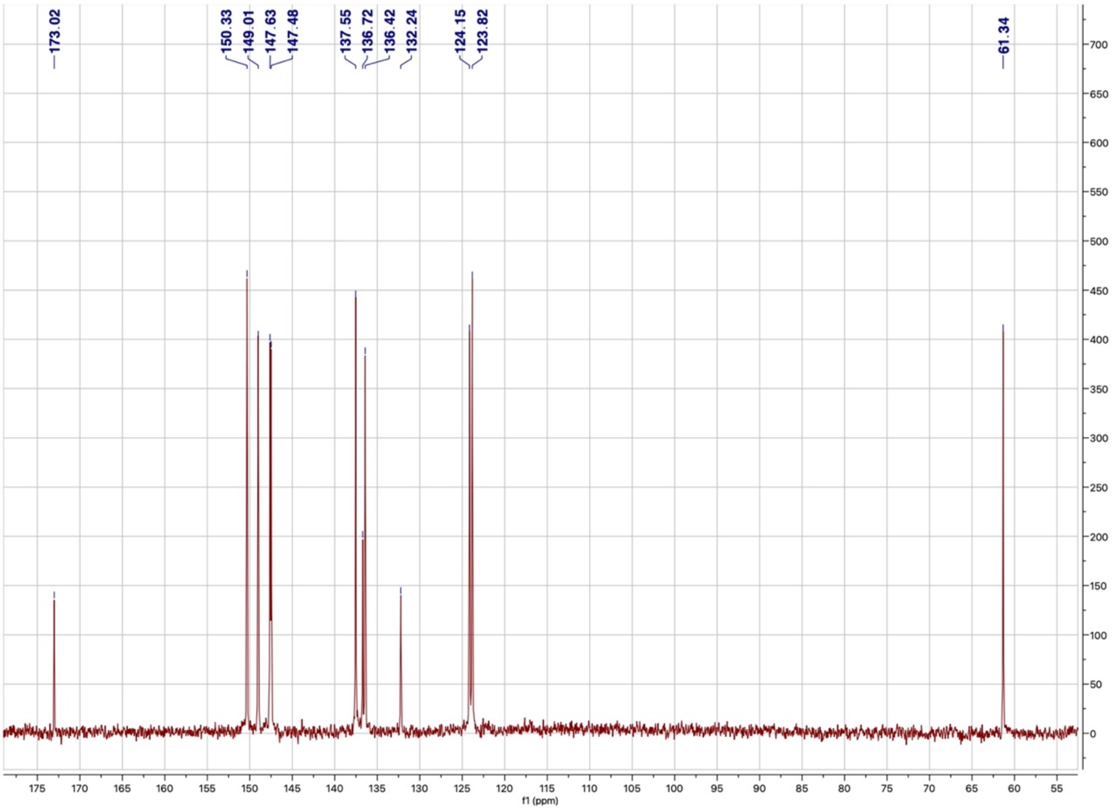

Carboxylic acid: 173.02 (-COOH), 149.01 (C_2), 147.63 (-C_6), 147.48 (-C_4), 132.24 (C_3); hydroxymethyl: 150.33 (C_2), 137.55 (C_6), 136.72 (C_3), 123.82 (C_5), 61.34 (CH_2OH).

Single-crystal X-ray Diffraction Results

CCDC 2362091 (Cif file: exp_3001)

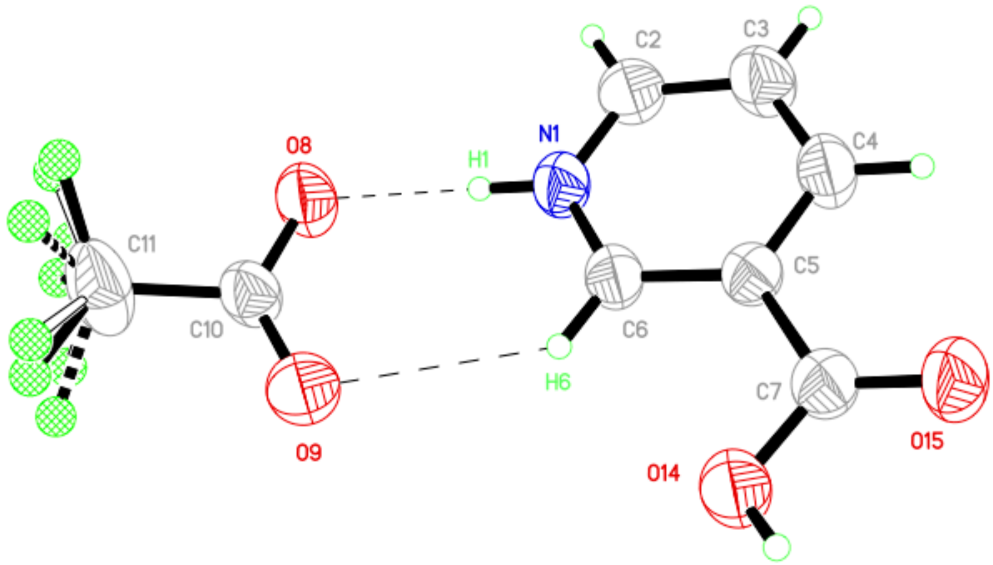

Figure S65. Crystal structure and numbering scheme for the **2362091** compound (**CA$_3$**). The displacement ellipsoids for the non-H atoms in the figure were drawn at the 50% probability level.

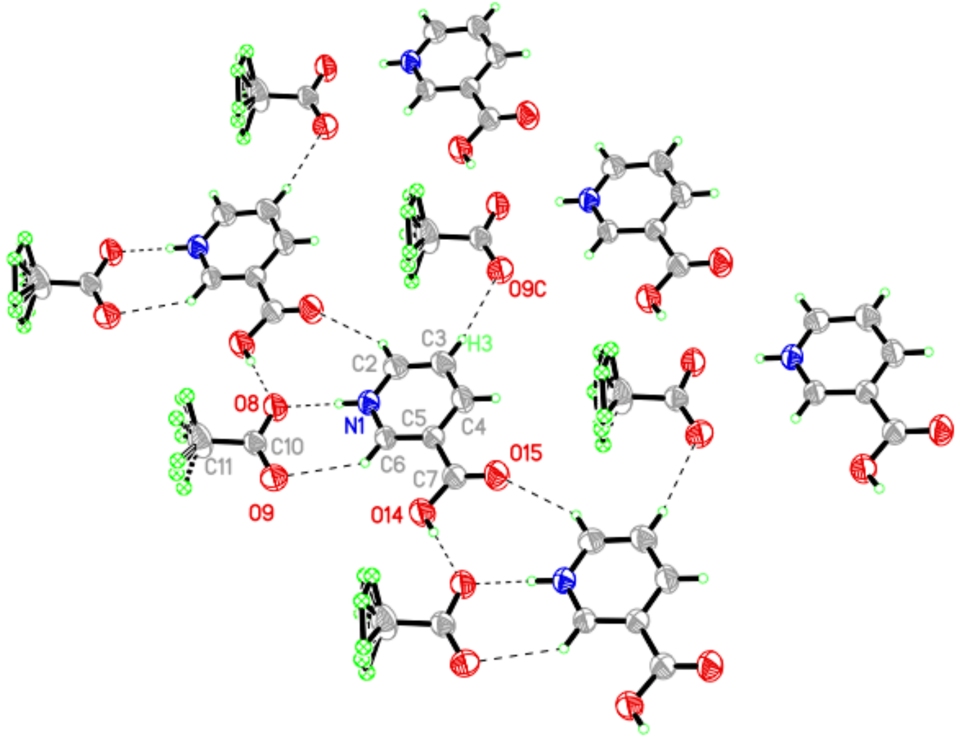

Figure S66. Crystal layer for the **CCDC 2362091** compound (**CA$_3$**). The displacement ellipsoids for the non-H atoms in the figure were drawn at the 50% probability level.

Table S1. Crystal data and structure refinement for **CCDC 2362091**.

Identification code	exp_3001 (CCDC 2362091)	
Empirical formula	C8 H6 F3 N O4	
Formula weight	237.14	
Temperature	293(2) K	
Wavelength	0.71073 Å	
Crystal system	Triclinic	
Space group	P -1	
Unit cell dimensions	a = 8.1826(14) Å	α= 114.477(17)°.
	b = 8.6086(15) Å	β= 93.359(14)°.
	c = 8.8937(16) Å	γ = 118.234(18)°.
Volume	476.34(17) Å^3	
Z	2	
Density (calculated)	1.653 Mg/m^3	
Absorption coefficient	0.168 mm^{-1}	
F(000)	240	
Crystal size	0.4 x 0.3 x 0.3 mm^3	
Theta range for data collection	4.327 to 29.099°.	
Index ranges	-10<=h<=11, -10<=k<=11, -11<=l<=11	
Reflections collected	3058	
Independent reflections	2102 [R(int) = 0.0207]	
Completeness to theta = 25.242°	98.8 %	
Absorption correction	Semi-empirical from equivalents	
Max. and min. transmission	1.00000 and 0.80154	
Refinement method	Full-matrix least-squares on F^2	
Data / restraints / parameters	2102 / 106 / 203	
Goodness-of-fit on F^2	1.047	
Final R indices [I>2sigma(I)]	R1 = 0.0595, wR2 = 0.1348	
R indices (all data)	R1 = 0.1057, wR2 = 0.1773	
Largest diff. peak and hole	0.442 and -0.323 e.Å^{-3}	

Table S2. Bond lengths [Å] and angles [°] for **CCDC 2362091**.

C(2)-N(1)	1.333(4)
C(2)-C(3)	1.367(4)
C(2)-H(2)	0.9300
C(3)-C(4)	1.378(4)
C(3)-H(3)	0.9300
C(4)-C(5)	1.392(4)
C(4)-H(4)	0.9300
C(5)-C(6)	1.365(4)
C(5)-C(7)	1.492(4)
C(6)-N(1)	1.332(3)
C(6)-H(6)	0.9300
C(7)-O(15)	1.208(3)
C(7)-O(14)	1.317(4)
C(10)-O(9)	1.218(3)
C(10)-O(8)	1.263(3)
C(10)-C(11)	1.520(4)
C(11)-F(12C)	1.257(8)
C(11)-F(13A)	1.285(6)
C(11)-F(13B)	1.293(8)
C(11)-F(14A)	1.307(6)
C(11)-F(12B)	1.332(7)
C(11)-F(13C)	1.367(7)
C(11)-F(14B)	1.381(8)
C(11)-F(14C)	1.386(8)
C(11)-F(12A)	1.441(6)
N(1)-H(1)	0.8600
O(14)-H(14)	0.8200
N(1)-C(2)-C(3)	120.0(3)
N(1)-C(2)-H(2)	120.0
C(3)-C(2)-H(2)	120.0
C(2)-C(3)-C(4)	118.7(3)
C(2)-C(3)-H(3)	120.7
C(4)-C(3)-H(3)	120.7
C(3)-C(4)-C(5)	120.2(3)
C(3)-C(4)-H(4)	119.9
C(5)-C(4)-H(4)	119.9

C(6)-C(5)-C(4)	118.5(3)
C(6)-C(5)-C(7)	121.3(3)
C(4)-C(5)-C(7)	120.2(2)
N(1)-C(6)-C(5)	120.0(3)
N(1)-C(6)-H(6)	120.0
C(5)-C(6)-H(6)	120.0
O(15)-C(7)-O(14)	124.8(3)
O(15)-C(7)-C(5)	123.0(3)
O(14)-C(7)-C(5)	112.2(2)
O(9)-C(10)-O(8)	127.8(3)
O(9)-C(10)-C(11)	118.9(3)
O(8)-C(10)-C(11)	113.3(3)
F(13A)-C(11)-F(14A)	113.2(5)
F(13B)-C(11)-F(12B)	109.4(8)
F(12C)-C(11)-F(13C)	108.1(7)
F(13B)-C(11)-F(14B)	107.8(7)
F(12B)-C(11)-F(14B)	103.9(7)
F(12C)-C(11)-F(14C)	111.7(7)
F(13C)-C(11)-F(14C)	100.9(7)
F(13A)-C(11)-F(12A)	103.8(5)
F(14A)-C(11)-F(12A)	101.5(4)
F(12C)-C(11)-C(10)	119.2(7)
F(13A)-C(11)-C(10)	116.5(5)
F(13B)-C(11)-C(10)	115.1(7)
F(14A)-C(11)-C(10)	114.5(4)
F(12B)-C(11)-C(10)	112.6(7)
F(13C)-C(11)-C(10)	109.2(6)
F(14B)-C(11)-C(10)	107.3(6)
F(14C)-C(11)-C(10)	106.2(5)
F(12A)-C(11)-C(10)	105.2(3)
C(6)-N(1)-C(2)	122.6(3)
C(6)-N(1)-H(1)	118.7
C(2)-N(1)-H(1)	118.7
C(7)-O(14)-H(14)	109.5

Symmetry transformations used to generate equivalent atoms:

Table S3. Torsion angles [°] for **CCDC 2362091**.

N(1)-C(2)-C(3)-C(4)	0.2(5)
C(2)-C(3)-C(4)-C(5)	0.0(5)
C(3)-C(4)-C(5)-C(6)	0.0(4)
C(3)-C(4)-C(5)-C(7)	-179.0(3)
C(4)-C(5)-C(6)-N(1)	-0.1(4)
C(7)-C(5)-C(6)-N(1)	178.9(3)
C(6)-C(5)-C(7)-O(15)	-173.7(3)
C(4)-C(5)-C(7)-O(15)	5.2(5)
C(6)-C(5)-C(7)-O(14)	6.2(4)
C(4)-C(5)-C(7)-O(14)	-174.9(3)
O(9)-C(10)-C(11)-F(12C)	92.4(11)
O(8)-C(10)-C(11)-F(12C)	-86.9(11)
O(9)-C(10)-C(11)-F(13A)	-125.0(6)
O(8)-C(10)-C(11)-F(13A)	55.7(6)
O(9)-C(10)-C(11)-F(13B)	159.3(14)
O(8)-C(10)-C(11)-F(13B)	-20.0(14)
O(9)-C(10)-C(11)-F(14A)	10.2(6)
O(8)-C(10)-C(11)-F(14A)	-169.1(4)
O(9)-C(10)-C(11)-F(12B)	33.1(8)
O(8)-C(10)-C(11)-F(12B)	-146.2(7)
O(9)-C(10)-C(11)-F(13C)	-142.7(7)
O(8)-C(10)-C(11)-F(13C)	38.0(7)
O(9)-C(10)-C(11)-F(14B)	-80.6(11)
O(8)-C(10)-C(11)-F(14B)	100.1(10)
O(9)-C(10)-C(11)-F(14C)	-34.6(9)
O(8)-C(10)-C(11)-F(14C)	146.1(9)
O(9)-C(10)-C(11)-F(12A)	120.8(4)
O(8)-C(10)-C(11)-F(12A)	-58.5(4)
C(5)-C(6)-N(1)-C(2)	0.3(4)
C(3)-C(2)-N(1)-C(6)	-0.3(5)

Symmetry transformations used to generate equivalent atoms:

Table S4. Hydrogen bonds for **CCDC 2362091** [Å and °].

D-H...A	d(D-H)	d(H...A)	d(D...A)	<(DHA)
C(2)-H(2)...O(15)#1	0.93	2.46	3.270(4)	146.2
C(3)-H(3)...O(9)#2	0.93	2.59	3.508(4)	168.8
C(6)-H(6)...O(9)	0.93	2.58	3.266(4)	130.6
N(1)-H(1)...O(8)	0.86	1.81	2.664(3)	172.3
O(14)-H(14)...O(8)#3	0.82	1.81	2.617(3)	167.3

Symmetry transformations used to generate equivalent atoms:

#1 x-1,y,z #2 x,y,z+1 #3 x+1,y,z
